# Comparative efficacy and safety of non-pharmacological interventions as adjunctive treatment for vascular dementia: a systematic review and network meta-analysis

**DOI:** 10.3389/fneur.2024.1397088

**Published:** 2024-07-12

**Authors:** Yunhao Yi, Yiwei Qu, Shimeng Lv, Guangheng Zhang, Yuanhang Rong, Ming Li

**Affiliations:** ^1^First Clinical Medical College, Shandong University of Traditional Chinese Medicine, Jinan, China; ^2^Office of Academic Affairs, Shandong University of Traditional Chinese Medicine, Jinan, China

**Keywords:** vascular dementia, non-pharmacological interventions, complementary and alternative therapies, cognitive function, network meta-analysis

## Abstract

**Objectives:**

The incidence of vascular dementia (VaD) is steadily rising annually, significantly impacting the mental well-being and overall quality of life of the elderly, and imposing substantial economic burdens on families and society. In recent years, non-pharmacological therapies as supplementary treatments for VaD have garnered significant attention and have been extensively utilized in clinical settings. Consequently, a network meta-analysis (NMA) was conducted by us to assess the effectiveness of various non-pharmacological therapies in the management of VaD.

**Design:**

We systematically searched seven databases from their inception up to January 2024 to identify randomized controlled trials focusing on non-pharmacological interventions for the treatment of VaD. The methodological quality and risk of bias were rigorously assessed utilizing the RoB 2.0 evaluation tool. The NMA was performed using R software and STATA 14 software, adhering to frequentist theory principles. Additionally, sensitivity analysis, meta-regression analysis, and funnel plot were conducted to assess the stability, heterogeneity, and publication bias, respectively.

**Results:**

The NMA included 91 eligible studies involving 7,657 patients. The NMA results indicated that in terms of improving Mini-Mental State Examination (MMSE), the following non-pharmacological interventions ranked higher based on *p*-value: acupuncture_moxibustion_ conventional treatment (ACUP_MB_CT) [P-score = 0.95; pooled mean difference (95% CI): 5.09 (3.82; 6.36)], fastigial nucleus stimulation_CT (FNS_CT) [0.87; 4.51 (2.59; 6.43)], ACUP_rehabilitation training_CT (ACUP_RT_CT) [0.84; 4.19 (2.77; 5.61)], repetitive transcranial magnetic stimulation_CT (rTMS_CT) [0.82; 3.98 (3.08; 4.88)], and aerobic exercise_CT (AE_CT) [0.82; 4.25 (1.86; 6.64)]. Regarding improvement in Activities of Daily Living Scale (ADL), the following non-pharmacological interventions ranked higher based on P-score: ACUP_MB_CT [0.98; 17.21 (13.19; 21.23)], ACUP_RT_CT [0.87; 14.32 (8.43; 20.22)], rTMS_CT [0.78; 11.83 (9.92; 13.75)], and ACUP_CT [0.73; 11.23 (9.26; 13.19)]. No significant adverse reactions were reported in the included studies.

**Conclusion:**

ACUP_MB_CT may be considered the most efficacious intervention for enhancing cognitive function and daily living skills in individuals diagnosed with VaD. Furthermore, ACUP_RT_CT, rTMS_CT, FNS_CT, ACUP_CT, and AE_CT also demonstrate significant clinical utility. Non-pharmacological interventions are unlikely to significantly increase adverse reactions and has a certain degree of safety.

**Systematic review registration:** https://www.crd.york.ac.uk/prospero/, identifier [CRD42024498902].

## 1 Introduction

Vascular dementia (VaD) is a syndrome of severe cognitive dysfunction caused by ischemic stroke, hemorrhagic stroke, and cerebrovascular disease causing hypoperfusion in brain regions such as memory, cognition, and behavior (1). Patients with VaD also have severe impairment of financial capacity (2). Notably, vascular risk factors (3) or comorbidities such as depressive symptoms (2) also accelerate the decline in cognitive function and financial capacity, severely affecting patients’ ability to perform daily life and quality of life. VaD, being the second most prevalent form of dementia following Alzheimer’s disease, comprises 15–20% of cases in Western nations (4) and as much as 40% in Asian countries and regions (5). Owing to the escalating occurrence of cerebrovascular ailments and improved post-stroke survival rates, the prevalence of VaD continues to increase (6). Therefore, Effective interventions are critical to the healthcare enterprise, healthcare professionals, caregivers, and patients themselves.

The pathogenesis of VaD is commonly believed to involve brain vascular disease that damages the frontal, temporal, and limbic systems, ultimately leading to cognitive impairment (7, 8). Research has found that degeneration, damage, and inflammation of the central nervous system caused by cerebrovascular disease can disrupt the blood–brain barrier (9, 10), whose permeability is closely associated with cognitive function (11). Through additional research, various cellular biological mechanisms and hypotheses such as excitotoxicity, oxidative stress, neuroinflammation, and neuronal apoptosis have been progressively uncovered (12–15). The interplay among diverse complex mechanisms (16) has somewhat contributed to the challenge of managing VaD in clinical settings. Presently, there are no specialized pharmacological agents available for VaD treatment. The treatment of VaD primarily focuses on treating primary brain vascular diseases and promoting brain function recovery to delay disease progression and extend life. Numerous drugs have been subjected to randomized controlled trials to test their efficacy, including acetylcholinesterase inhibitors such as donepezil and galantamine, N-methyl-D-aspartate receptor (NMDAR) antagonists like memantine, and drugs that improve brain function. Nonetheless, a network meta-analysis (NMA) has revealed that though these medications can partially ameliorate clinical symptoms, their efficacy is largely comparable, yielding unsatisfactory long-term outcomes (17). The fact that their efficacy often entails gastrointestinal, hepatic, and renal adverse reactions poses a significant challenge (18). In recent years, non-pharmacological therapies have been widely used in the treatment of VaD due to their advantages such as simplicity, affordability, and minimal adverse effects. Therefore, the exploration of non-pharmacological therapies holds significant value.

In the past, traditional meta-analyses have indicated that non-pharmacological therapies are effective in enhancing cognitive function and activities of daily living in patients with VaD (19–21). The study conducted by You and colleagues (19) reported the beneficial effects of hyperbaric oxygen therapy for VaD; however, the limited sample size in their study might have led to an overestimation of the therapy’s efficacy. Chen et al. (20) demonstrated that acupuncture could be advantageous for VaD; however, their control group encompassed both conventional treatments and non-conventional interventions like proprietary Chinese medicines and Chinese herbal tonics. Among these studies, only the research conducted by Jiang et al. (21) incorporated comparisons of non-pharmacological interventions in subgroup analyses, albeit with only two studies included. Hence, these meta-analyses failed to provide robust evidence, primarily comparing against conventional treatments. NMAs are considered the highest level of evidence in treatment guidelines (22). However, existing network meta-analyses of non-pharmacological interventions have mainly focused on mild cognitive impairment (23) or Alzheimer’s disease (24). While there is a NMA for VaD, it primarily focuses on the aspect of acupuncture (25). Their research found that combined acupuncture therapy is superior to single intervention in improving cognitive function and activities of daily living. However, clinicians face challenges in selecting the most suitable interventions from a range of non-pharmacological therapies. Therefore, this study utilizes a NMA to comprehensively and systematically compare the impacts of different non-pharmacological therapies on enhancing cognitive function and activities of daily living in patients with VaD. This research also provides evidence-based support for clinicians in choosing treatment strategies.

## 2 Materials and methods

We performed a systematic review and NMA according to the Preferred Reporting Items for Systematic Reviews and Meta-analyses (PRISMA) statement (26). In addition, this study has been registered with PROSPERO, under the number 42024498902.

### 2.1 Search strategies

We searched the data in PubMed, Embase, Cochrane Library, China National Knowledge Infrastructure (CNKI), Wanfang Database (Wanfang), China Science and Technology Journal Database (VIP) and Chinese Biomedical Literature Database (SinoMed) from the database’s inception through January 2024 using Medical Subject Headings (MeSH) for “vascular dementia” and “complementary therapies” search terms in Supplementary Appendix 1. In order to ensure the comprehensiveness of the study, we conducted additional searches by reviewing the reference lists of previously published systematic reviews that were identified through the Cochrane Database of Systematic Reviews (search terms: vascular dementia, complementary therapies; limits: none) and PubMed (search terms: vascular dementia, complementary therapies; limits: systematic reviews or meta-analysis). We also searched the Chinese Clinical Trial Registry and Clinicaltrials.gov for some unpublished clinical trials.

### 2.2 Eligibility criteria

The inclusion criteria were based on the PICOS (participants, interventions, comparators, outcomes, and study design) approach (26). Studies included in this meta-analysis must meet the following criteria and report specific experimental characteristics: (a) Participants had to meet the diagnostic criteria for VaD, including the Chinese Guidelines for the Diagnosis and Treatment of Dementia and Cognitive Impairment and the Diagnostic and Statistical Manual of Mental Disorders (DSM-V). Dementia within 3 months of stroke, sudden onset of cognitive decline or fluctuating or step-like progressive cognitive impairment. Neuropsychological, magnetic resonance imaging, and electron computed tomography scans are required for the diagnosis of VaD. Participants’ eligibility is not limited by age, gender, race, geographic region, ethnicity, or duration of illness. (b) The intervention in the study must incorporate a minimum of one non-pharmacological therapy. Detailed information about these therapies is provided in Supplementary Appendix 2. Only non-pharmacological therapies can be used as the experimental group for comparison with the control group. (c) The control group received conventional anti-dementia drug treatment and symptomatic supportive treatment. Anti-dementia drugs such as donepezil, galantamine, and memantine were used. For supportive treatment, antiplatelet agents like aspirin and clopidogrel, as well as conventional lipid-lowering drugs, hypoglycemic agents, and antihypertensive medications, were administered. In head-to-head studies, any single or combination of non-pharmacological therapies may be employed as the treatment modality. (d) The study must incorporate at least one outcome measure, such as MMSE and ADL. (e) The study design of the included articles must follow a randomized controlled trial methodology.

Exclusion criteria for this study were: (a) patients with Alzheimer’s disease or dementia caused by other factors, as well as those with various mild cognitive impairments and non-dementia vascular cognitive impairments; (b) patients who meet the diagnosis of depression or other psychiatric disorders or who have severe neurological impairments that interfere with neuropsychological assessment; (c) studies with duplicate publications or duplicate data; (d) non-RCT studies, such as meta-analyses, reviews, theoretical discussions, clinical experiences, animal experiments, etc.; (e) Unable to access the original text or extract the mean and standard deviation of the study, or unable to obtain the research data from the authors; (f) studies that did not have one primary endpoint or secondary endpoint indicator as a primary endpoint indicator.

### 2.3 Outcome indicators

The Mini-Mental State Examination (MMSE) is primarily used to provide a comprehensive, accurate, and rapid assessment of the intellectual status and degree of cognitive impairment in patients with VaD. Additionally, the Barthel Index is utilized as the activities of daily living scale (ADL) to evaluate the patient’s ability to perform daily activities, assessing self-care and functional independence. Adverse reactions from various randomized controlled trials (RCTs), including symptoms like dizziness, headache, syncope, and hematoma, will be recorded to assess the safety of different treatments. Thus, the primary outcome measure in our study is the MMSE, with ADL as the secondary outcome measure (Supplementary Appendix 3).

### 2.4 Data collection

Two independent researchers (YYH and ZGH) screened potentially eligible papers by reading the titles, abstracts, and full texts of their respective articles based on the inclusion and exclusion criteria. Two researchers (YYH and ZGH) independently retrieved publication details, patient characteristics (such as the number of patients, gender distribution, and disease duration), pertinent intervention specifics (including treatment period, frequency, and time), as well as the mean and standard deviation of outcome measures. If the standard deviation (SD) was not explicitly provided, we derived it by utilizing standard errors (SE), 95% confidence intervals, quartiles, upper and lower range limits of variability, and disparities in baseline values. For image type data, GetData software was used to perform the extraction. If data remained unavailable, we would then reach out to the respective authors of the publications. If discrepancies arise, consultation with a third researcher (QYW) would be sought to reach a resolution.

### 2.5 Quality assessment and CINeMA

Two investigators (YYH and ZGH) referred to the Cochrane Collaboration’s recommendation of the latest Risk of Bias assessment tool 2.0 (ROB 2.0) for risk of bias assessment (27). ROB 2.0 comprises of five modules: randomization process, deviations from intended interventions, missing outcome data, measurement of the outcome, selection of the reported result. The results of each module were assessed using the modular decision pathway diagrams. Ultimately, these results were summarized to determine the overall assessment of bias, which was categorized as “Low risk,” “Some concerns,” or “High risk” based on the contents of the literature. We used the online application Confidence in Network Meta-Analysis (CINeMA) to assess the certainty of evidence for each outcome, categorizing the evidence into four levels: high, moderate, low, and very low (28). It is worth noting that interactions between different domains may influence each other. Therefore, we analyzed all six CINeMA domains collectively to prevent duplicative situations that could jeopardize the overall quality of evidence due to interconnected issues.

### 2.6 Data synthesis and analysis

We conducted statistical analysis using R software (version 4.3.2) and Stata software (14.0) (29, 30). Within a frequentist framework, we employed the “meta” and “netmeta” packages in R for NMA. Continuous variables were represented by mean difference (MD), and their 95% confidence intervals (CI) were calculated. We utilized the “network map” command in Stata to create a network diagram. Here, node size indicated the sample size of interventions, while the thickness of edges represented the number of studies comparing two direct interventions. Furthermore, our forest plot presented MD summary values and their 95%CI for all comparisons. Additionally, the P-score in the forest plot assessed the efficacy of different non-pharmacological therapies, with higher scores denoting superior efficacy. Simultaneously, we conducted cluster analysis on two distinct outcome indicators to identify interventions with superior combined efficacy. Global heterogeneity and inconsistency were assessed utilizing the “decomp.design” function in R software. The global I^2^ statistic was employed to evaluate heterogeneity, where I^2^ values exceeding 50% signify notable heterogeneity, prompting the application of a random-effects model. Furthermore, global consistency and the Separated Indirect From Direct Evidence (SIDE) test were utilized to evaluate overall and local inconsistency (31). The R package “gemtc” was used to pinpoint sources of heterogeneity in the study, including variables like publication year, sample size, gender, age, illness duration, treatment duration, treatment frequency, and treatment timing. The stability of treatment effects across different outcome indicators in network meta-regression was evaluated by computing the mean values of covariates from the models. Studies with treatment durations outside the 4–16 weeks range and those exhibiting high bias risk were excluded, followed by a sensitivity analysis. To identify publication bias and small study effects within the included studies, comparison-adjusted funnel plots were employed.

## 3 Results

### 3.1 Literature screening process and basic characteristics

**Figure 1:**
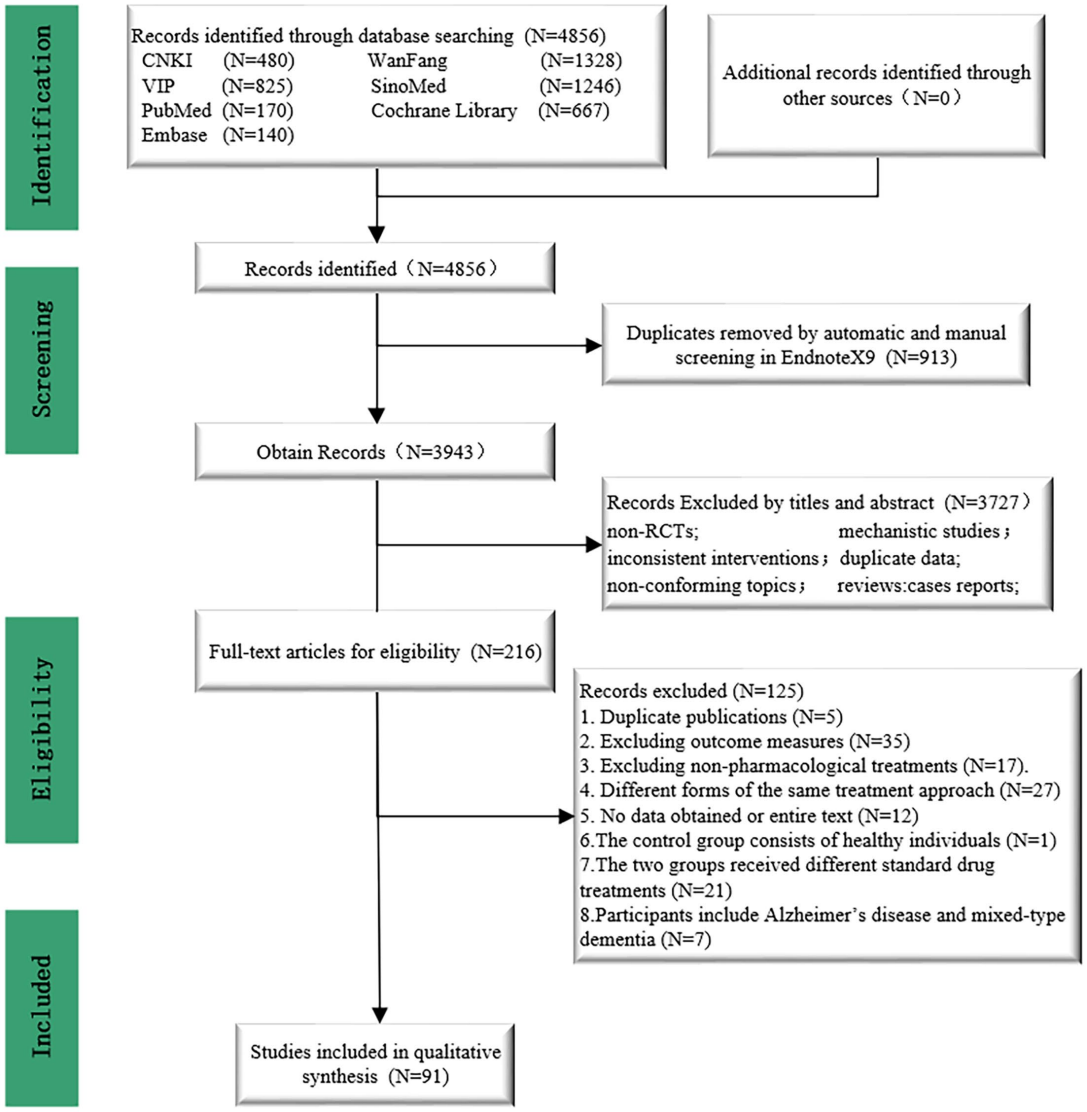
PRISMA flow diagram for the entire review.

Figure 1 illustrates the specific details of the literature screening process. After searching relevant literature databases, a total of 4,856 articles were obtained. Following the removal of 913 duplicates using Endnote X9 software, 3,943 articles were excluded based on abstracts and titles, leaving 216 full-text articles. Subsequently, two researchers finalized the inclusion of 91 studies based on the established inclusion and exclusion criteria (32–122). The specific details of the literature screening process can be seen in Figure 1. Table 1 contains information about the 91 studies of RCTs published between 2005 and 2023 that met the criteria for natriuresis. The 91 studies included a total of 7,657 participants, with 4,235 (55.31%) males and 3,422 (44.69%) females, predominantly elderly individuals, with sample sizes ranging from 33 to 234 and an average disease duration of 19.07 months (SD 13.42). Among the 91 studies, 21 different treatment modalities were included (Supplementary Appendix 2), with an average treatment duration of 8.16 weeks (SD 4.53), treatment frequencies ranging from 1 to 14 times per week (average 6.4 times, SD 1.79), and treatment durations per session ranging from 16 to 80 min (average 45.1 min, SD 15.41) (Supplementary Appendix 4). Basic characteristics of the included studies such as authors, publication year, participant information (average age, gender), interventions, duration, and outcome indicators were summarized in Table 1. The detailed interventions for each study are in Supplementary Appendix 4.

**Table 1:** Characteristics of the included studies.

Included studies	Sample (E/C)	Sex(M/F)		Age		Interventions		Treatment course	Outcomes
		E	C	E	C	E	C		
Hao et al. (32)	60/60	32/28	29/31	68.78 ± 4.15	69.49 ± 4.77	rTMS_CT	CT	21d*3	MMSE
Ren et al. (33)	39/39	24/15	23/16	69.12 ± 9.89	68.91 ± 9.02	rTMS_CT	CT	2 m	MMSE
Yang (34)	45/45	21/24	23/22	66.75 ± 9.36	67.37 ± 8.91	rTMS_CT	CT	6w	MMSE ADL ARs
Li and Zhang (35)	42/38	46/34		62.4 ± 5.1		rTMS_CT	CT	3 m	MMSE ADL
Li et al. (36)	48/48	25/23	26/22	65.22 ± 7.03	65.31 ± 7.26	rTMS_CT	CT	4w	MMSE ADL
Guo et al. (37)	20/20/20	rTMS_CT: 12/8 EA: 10/10	13/7	rTMS_CT: 76.3 ± 3.5 EA: 75.9 ± 4.3	76.3 ± 3.9	rTMS_CT	CT	6 m	MMSE ADL ARs
						EA			
Pan et al. (38)	40/40	24/16	26/14	69.14 ± 6.89	68.51 ± 6.78	rTMS_ACUP_MB_CT	CT	12w	MMSE
Wu et al. (39)	20/20	13/7	11/9	67.6 ± 6.8	66.9 ± 7.3	rTMS_ACUP_MB_CT	CT	12w	MMSE
Cheng and Tan (40)	40/40	22/18	21/19	71.54 ± 7.28	70.23 ± 7.12	ACUP_CT	CT	30d	MMSE ADL
Meng and Han (41)	30/30	NA		NA		ACUP_CT	CT	6w	MMSE
Han et al. (42)	59/59	30/29	29/30	64 ± 9	66 ± 8	ACUP_CT	CT	8w	MMSE ADL
Qiao and Hu (43)	40/40	25/15	25/15	69.13 ± 6.31	68.21 ± 6.11	ACUP_CT	CT	20d	MMSE
Chen (44)	40/20	24/16	16/7	64.38 ± 4.76	65.29 ± 4.87	ACUP_CT	CT	30d	MMSE
Wang et al. (45)	45/45	34/11	32/13	71.32 ± 5.06	71.44 ± 5.13	ACUP_CT	CT	8w	MMSE ADL
Hu et al. (46)	44/44	24/20	23/21	68 ± 9	67 ± 8	ACUP_CT	CT	8w	MMSE ADL ARs
Feng et al. (47)	47/47	31/16	32/15	64.29 ± 9.13	63.97 ± 9.15	ACUP_CT	CT	8w	MMSE ARs
Ye et al. (48)	30/30	17/13	15/15	58.23 ± 5.83	58.01 ± 5.94	ACUP_CT	CT	4w	MMSE
Zhang and Qu (49)	45/45	25/20	27/18	66.4 ± 7.6	65.9 ± 8.2	ACUP_CT	CT	12w	MMSE ADL
Cui et al. (50)	30/30	14/16	18/12	67 ± 5	68 ± 5	ACUP_CT	CT	4w	MMSE
Yu et al. (51)	32/31	16/14	15/15	71.15 ± 336	70.68 ± 3.17	ACUP	CT	12w	MMSE
Hu et al. (52)	34/34	22/12	21/13	64.48 ± 1	66.1 ± 1	ACUP	CT	90d	MMSE ARs
Tan et al. (53)	30/30	17/13	19/11	66.73 ± 3.12	66.30 ± 3.27	ACUP	CT	30d	MMSE
Zhang et al. (54)	30/30	19/11	18/12	65.38 ± 5.76	66.29 ± 6.87	ACUP	CT	8w	MMSE
Zheng et al. (55)	38/37	21/17	22/15	67.73 ± 4.91	68.08 ± 5.11	EA_CT	CT	8w	MMSE
Gao et al. (56)	30/30	14/16	13/17	72.27 ± 4.08	71.57 ± 5.04	EA_CT	CT	8w	MMSE
Yao (57)	30/30	18/12	16/14	68.17 ± 5.86	67.37 ± 5.24	EA_CT	CT	30d	MMSE
Xu (58)	30/40/20	EA_CT: 21/9 EA: 25/15	12/8	EA_CT: 63.7 ± 7.67 EA: 62.6 ± 8.44	63.3 ± 8.06	EA_CT	CT	3 m	MMSE
						EA			
Liu et al. (59)	30/30	16/14	17/13	72.46 ± 8.12	72.05 ± 8.06	RT_CT	CT	12w	MMSE
Yang (60)	30/30/30	EA_CT: 9/21 EA: 14/16	18/12	EA_CT: 62.9 ± 4.89 EA: 61.8 ± 5.18	63.3 ± 4.27	EA_CT	CT	2 m	MMSE
						EA			
Peng et al. (61)	24/27/26	EA_C: 15/9 EA: 19/8	17/9	EA_CT: 63.71 ± 9.32 EA: 65.78 ± 6.417	66.00 ± 9.11	EA_CT	CT	6w	MMSE
						EA			
Zhao et al. (62)	26/23/24	NA	NA	45–80		EA_CT	CT	6w	MMSE
						EA			
Huiming (63)	32/32	20/12	17/15	72.91 ± 6.12	69.22 ± 5.71	EA	CT	3 m	MMSE ADL
Yin et al. (64)	30/30	18/12	17/13	62.67 ± 5.1	62.82 ± 5.4	EA	CT	12w	MMSE
Li et al. (65)	28/28	15/13	16/12	65.1 ± 11.3	66.4 ± 13.6	EA	CT	4w	MMSE ADL
Wang et al. (66)	35/35	19/16	18/17	72.9 ± 4.9	72.9 ± 5.1	MB_CT	CT	8w	MMSE ARs
Sheng and Cai (67)	30/30	16/14	18/12	51–75	53–75	MB_CT	CT	4w	MMSE
Gao et al. (68)	40/40	49/31		N/A		MB_CT	CT	8w	MMSE
Luo et al. (69)	36/36/36	MB: 13/17 ACUP: 15/15	12/18	MB: 72.06 ± 3. 36 ACUP: 71.75 ± 3. 87	71.75 ± 3. 87	MB	CT	8w	MMSE
						ACUP			
Fan (70)	50/50	31/19	32/18	67.46 ± 2.78	67.11 ± 2.41	ACUP_MB_CT	CT	12w	MMSE ARs
Zhao and Chen (71)	38/38	21/17	20/18	54.2	52.2	ACUP_MB_CT	CT	100d	MMSE
Ma et al. (72)	30/30	17/13	16/14	67.35 ± 5.62	67.82 ± 5.56	ACUP_MB_CT	ACUP_CT	8w	MMSE
Wang et al. (73)	50/50	30/20	29/21	72.13 ± 4.15	71.21 ± 4.21	ACUP_MB_CT	ACUP_CT	6w	MMSE ADL
Wang et al. (74)	33/33	31/35		69.7 ± 3.1		RT_CT	CT	3 m	MMSE ADL
Wang et al. (75)	32/31	25/7	24/7	67.07 ± 7.91	67.48 ± 7.22	RT_CT	CT	12w	MMSE
Sun and Gao (76)	32/32	19/13	20/12	63 ± 9	65 ± 9	RT_CT	CT	12w	MMSE ADL
Zhai et al. (77)	28/28	16/12	13/15	68.4 ± 6.6	71.6 ± 6.9	RT_CT	CT	6 m	MMSE ADL
Wu et al. (78)	43/43	28/15	26/17	72.5 ± 10.5	70.0 ± 10.0	CFT_CT	CT	2 m	MMSE ADL
Ji et al. (79)	35/37	NA		51.7 ± 13.9		CFT_CT	CT	2 m	ADL
Zhu et al. (80)	40/40	23/17	24/16	70.11 ± 4.10	69.76 ± 3.64	CFT_CT	CT	3 m	MMSE ADL
Qu et al. (81)	30/30	21/9	19/11	68.3 ± 7.5	67.5 ± 6.8	CFT_CT	CT	8w	MMSE
Zhao (82)	83/83	81/85		68.5 ± 3.5		HBO_CT	CT	3 m	MMSE ADL ARs
Liu and Gao (83)	48/48	28/20	27/21	65.3 ± 4.6	65.8 ± 4.1	HBO_CT	CT	10d*(4–5)	MMSE
Chen (84)	41/41	22/19	21/20	64.2 ± 7.2	63.7 ± 9.1	HBO_CT	CT	10d*5	MMSE
Liu (85)	32/32	19/13	18/14	62.8 ± 7.1	62.2 ± 7.5	HBO_CT	CT	10d*5	MMSE
Lei (86)	30/30	16/14	17/13	66.8 ± 3.7	66.7 ± 3.9	HBO_CT	CT	3w	MMSE
Sun et al. (87)	30/30	16/14	18/12	67.0 ± 4.9	68.0 ± 5.6	HBO_CT	CT	24d	MMSE ARs
Wang et al. (88)	40/40	22/18	20/20	66.2 ± 9.6	67.0 ± 8.9	HBO_CT	CT	60d	MMSE ADL
Wang et al. (89)	32/32	20/12	21/11	70.4 ± 8.5	70.8 ± 8.1	HBO_CT	CT	12w	MMSE
Tang et al. (90)	100/100	54/46	55/45	65.1 ± 5.9	66.2 ± 6.5	HBO_CT	CT	NA	MMSE
Li (91)	36/36	20/16	21/15	65.2 ± 6.9	65.3 ± 6.9	HBO_CT	CT	10d*5	MMSE
Feng (92)	39/39	24/15	25/14	67.81 ± 6.02	67.22 ± 5.76	HBO_CT	CT	1 m	MMSE ADL
Hu (93)	60/60	26/34	31/29	72.12 ± 5.10	73.47 ± 5.29	HBO_CT	CT	1 m	MMSE ARs
Wang et al. (94)	35/35	18/17	17/18	67.5 ± 9.8	68 ± 9.3	HBO_CT	CT	8w	MMSE ADL
Yang et al. (95)	49/49	47/51		73.1 ± 11.2		HBO_CT	CT	4w	MMSE ARs
Li (96)	45/45	25/20	24/21	68.59 ± 4.07	68.73 ± 4.18	HBO_CT	CT	14d	MMSE ADL
Wang and Zhai (97)	40/40	22/18	20/20	64.2 ± 7.2	63.7 ± 9.1	HBO_CT	CT	10d*5	MMSE
Xia (98)	30/30	16/14	13/17	55–73	58–76	HBO_CT	CT	4w	MMSE ARs
Bao et al. (99)	46/43	21/25	19/24	72.6 ± 6.8	71.6 ± 8.2.	HBO_CT	CT	10d*4	MMSE
Wu et al. (100)	50/50/50	HBO_CT: 27/23 HBO: 26/24	28/22	HBO_CT: 64.2 ± 1.90 HBO: 62.2 ± 2.91	63.2 ± 2.11	HBO_CT	CT	3w	MMSE ARs
						HBO			
Song (101)	31/31/31	N/A		N/A		HBO_CT	CT	3w	MMSE ARs
						HBO			
Bu (102)	32/32/32	N/A		N/A		HBO_CT	CT	3w	MMSE
						HBO			
Liu (103)	52/52	22/28	27/25	64.69 ± 1.98	64.83 ± 2.27	EMGBFB_CT	CT	4w	MMSE ADL
Ran and Yang (104)	39/39	22/17	25/14	66.81 ± 6.02	67.03 ± 5.89	EMGBFB_CT	CT	4 m	MMSE ADL ARs
Du et al. (105)	42/42	34/8	32/10	71.14 ± 6.88	70.87 ± 7.02	EMGBFB_CT	CT	6 m	MMSE
Liu et al. (106)	57/57	30/27	27/30	68.56 ± 4.27	69.89 ± 4.71	EMGBFB_CT	CT	1 m	MMSE ADL ARs
Cai et al. (107)	47/47	26/21	25/22	64.4 ± 5.4	62.71 ± 5.83	EMGBFB_CT	EMGBFB	3 m	MMSE
Chen et al. (108)	35/33	20/15	19/14	72.91 ± 3.20	71.63 ± 4.57	FNS_CT	CT	10d*2	MMSE
Wu (109)	20/13	24/9		66.8 ± 4.7		FNS_CT	CT	15d	MMSE ADL
Dai et al. (110)	25/21	15/10	12/9	75.3 ± 8.2	76.2 ± 8.3	FNS_CT	CT	4w	MMSE ADL ARs
Li et al. (111)	48/48	28/20	26/22	62.17 ± 8.01	60.86 ± 7.45	ACUP_RT_CT	RT_CT	8w	MMSE
Wu et al. (112)	50/50	30/20	32/18	60.89 ± 3.91	61.02 ± 3.11	ACUP_RT_CT	RT_CT	4w	MMSE
Li et al. (113)	35/35	19/16	17/18	62 ± 6	64 ± 7	ACUP_RT_CT	RT_CT	12w	MMSE ADL
Li et al. (114)	60/60	68/52		55–76		ACUP_RT_CT	RT_CT	12w	MMSE
Wang et al. (115)	34/34	21/13	24/10	61.67 ± 7.76	64.8 ± 7.76	ACUP_RT_CT	CT	8w	MMSE ADL
Shi (116)	42/42/42	AA_MB: 28/14 AA: 27/15	30/12	AA_MB: 68.92 ± 6.11 AA: 67.90 ± 6.20	69.12 ± 5.66	AA_MB	CT	12w	MMSE
						AA			
Chen et al. (117)	84/79	48/36	40/39	70.41 ± 7.32	71.56 ± 6.27	AA	CT	12w	MMSE
Kuang et al. (118)	78/78/78	NA		NA		AA_MB	CT	12w	MMSE
						AA			
Wang et al. (119)	20/20	12/8	13/7	65.1	66.4	ACUP	CT	12w	MMSE
Wang et al. (120)	30/30	17/13	18/12	62. 86 ± 4.51	66. 10 ± 3.84	ACUP	CT	8w	MMSE
Liu (121)	62/62	34/28	30/32	64.6 ± 3.3	66.8 ± 3.9	AE_CT	CT	30d	MMSE ADL
Li (122)	46/46	32/14	34/12	66.25 ± 7.03	65.37 ± 6.79	EMGBFB_CT	CT	8w	MMSE

M, male; F, female; E, experimental group; C, control; m, months; w, weeks; d, days; AA, auricular acupuncture; ACUP, acupuncture; AE, Aerobic exercise; CFT, Cognitive function training; CT, Conventional treatment; EMGBFB, electromyographic biofeedback; EA, electroacupuncture; FNS, Fastigial nucleus stimulation; HBO, hyperbaric oxygen therapy; MB, moxibustion; RT, Rehabilitation training; rTMS, Repetitive Transcranial Magnetic Stimulation; MMSE, Mini-Mental State Examination; ADL, Activities of Daily Living Scale; ARs, Adverse reactions.

There were 21 treatment modalities forming 27 direct comparisons, including auricular acupuncture (AA) vs. AA_moxibustion (AA_MB) (2 comparisons), AA vs. conventional treatment (CT) (3 comparisons), AA_MB vs. CT (2 comparisons), acupuncture (ACUP) vs. CT (7 comparisons), ACUP vs. MB (1 comparison), ACUP_CT vs. ACUP_MB_CT (2 comparisons), ACUP_CT vs. CT (11 comparisons), ACUP_MB_CT vs. CT (2 comparisons), ACUP_rehabilitation training_CT ACUP_RT_CT vs. CT (1 comparison), ACUP_RT_CT vs. RT_CT (4 comparisons), aerobic exercise_CT (AE_CT) vs. CT (1 comparison), cognitive function training_CT (CFT_CT) vs. CT (4 comparisons), electroacupuncture (EA) vs. CT (7 comparisons), EA_CT vs. CT (8 comparisons), electromyographic biofeedback_CT (EMGBFB_CT) vs. CT (5 comparisons), Fastigial nucleus stimulation_CT (FNS_CT) vs. CT (3 comparisons), hyperbaric oxygen therapy (HBO) vs. CT (3 comparisons), HBO_CT vs. CT (21 comparisons), MB vs. CT (1 comparison), MB_CT vs. CT (3 comparisons), RT_CT vs. CT (5 comparisons), repetitive transcranial magnetic stimulation_ACUP_MB_CT (rTMS_ACUP_MB_CT) vs. CT (2 comparisons), rTMS_CT vs. CT (6 comparisons), EA vs. EA_CT (4 comparisons), EA_CT vs. rTMS_CT (1 comparison), EMGBFB vs. EMGBFB_CT (1 comparison), HBO vs. HBO_CT (3 comparisons).

### 3.2 Bias risk assessment of involved literature

**Figure 2:**
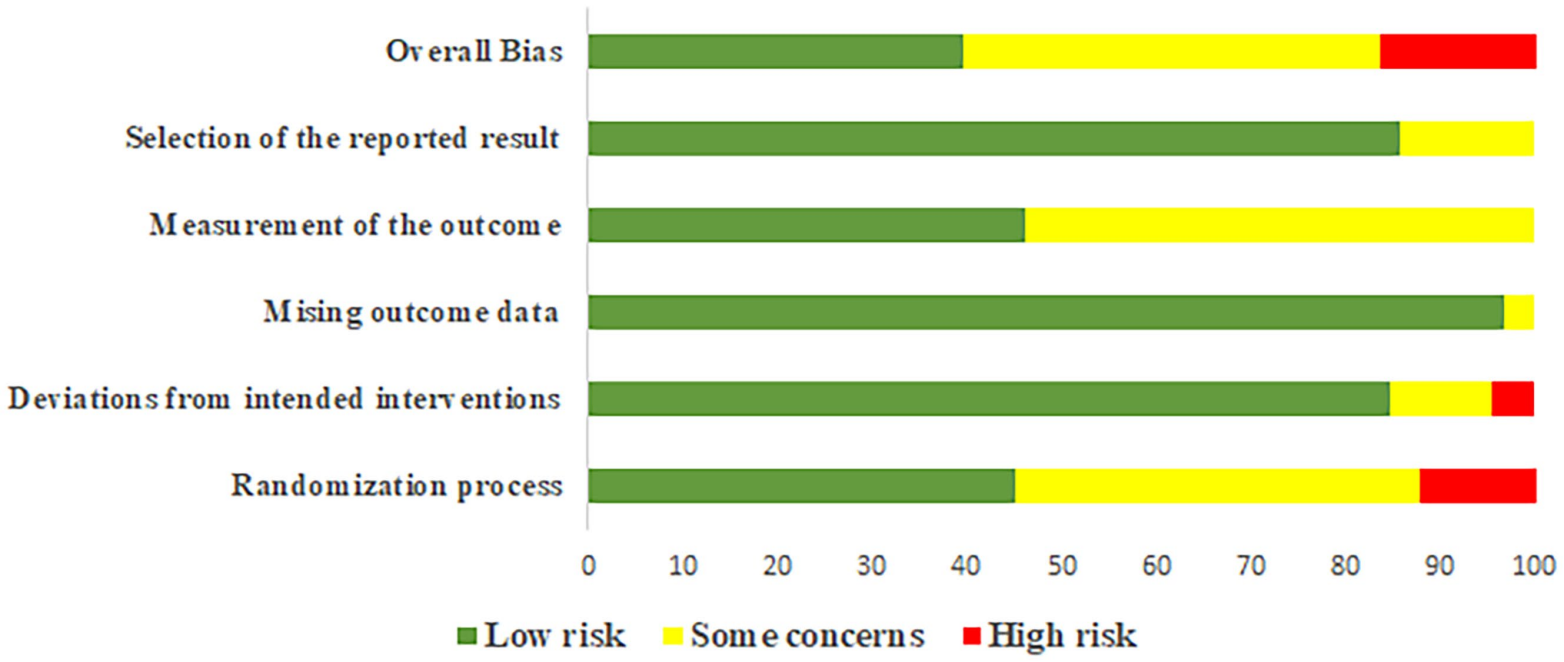
Results of risk of bias evaluation of included studies.

The bias risk of each study can be identified in Supplementary Appendix 5, while the summary of bias risk across all studies is depicted in Figure 2. The proportion of studies with low bias risk during the randomization process was 45.05%, deviations from intended interventions was 84.62%, missing outcome data stands at 96.70%, the measurement of outcomes was 46.15%, and the selection of reported results was 85.71%. Overall, the proportion of studies with high bias risk is 16.48%, medium bias risk was 43.96%, and low bias risk accounts for 39.56%.

### 3.3 Network meta-analysis

#### 3.3.1 MMSE

**Figure 3:**
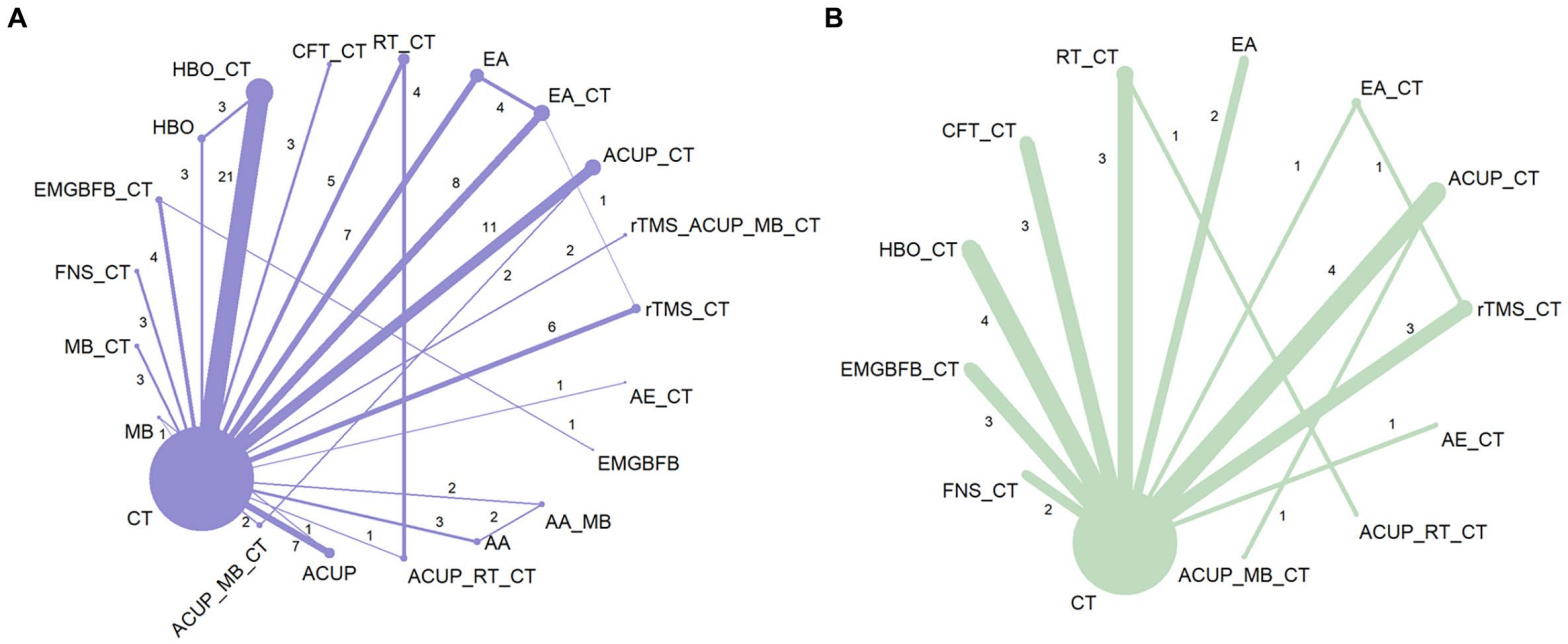
Network graph of network meta-analysis for main outcomes. **(A)** Mini-Mental State Examination (MMSE), **(B)** Activities of Daily Living Scale (ADL). AA, auricular acupuncture; ACUP, acupuncture; AE, Aerobic exercise; CFT, Cognitive function training; CT, Conventional treatment; EMGBFB, electromyographic biofeedback; EA, electroacupuncture; FNS, Fastigial nucleus stimulation; HBO, hyperbaric oxygen therapy; MB, moxibustion; RT, Rehabilitation training; rTMS, Repetitive Transcranial Magnetic Stimulation.

**Figure 4:**
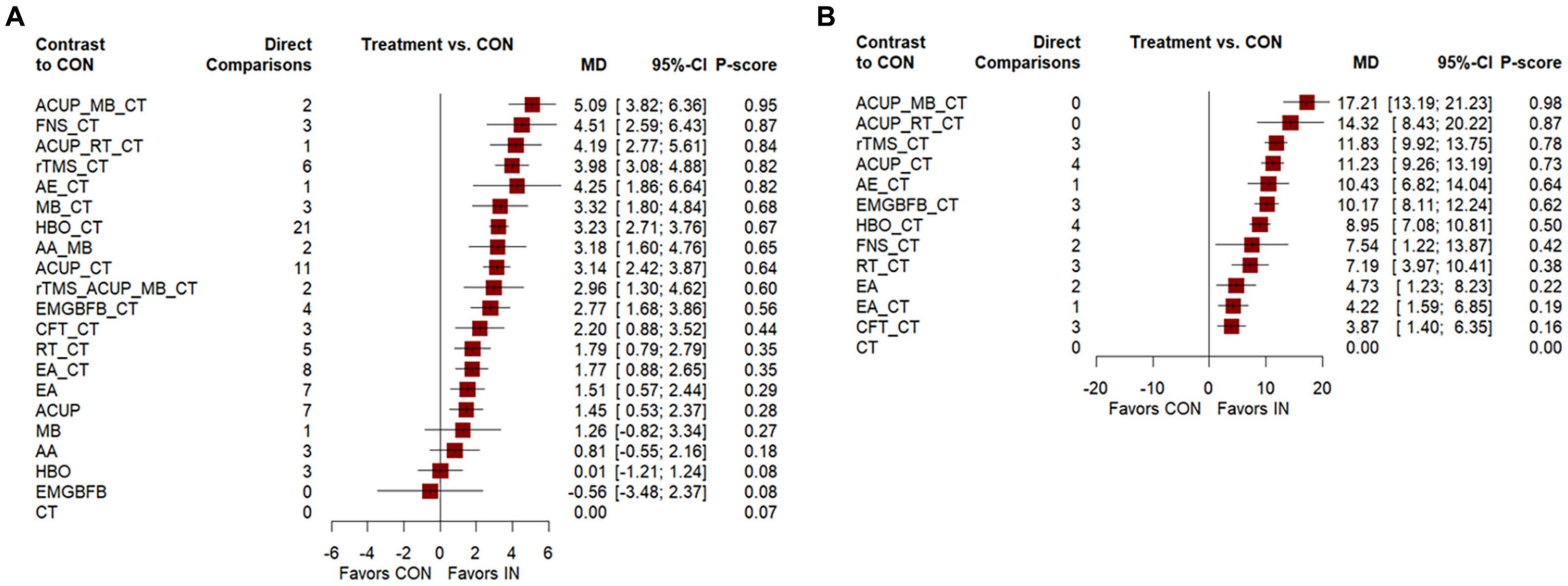
Forest plot of network meta-analysis for main outcomes. **(A)** Mini-Mental State Examination (MMSE), **(B)** Activities of Daily Living Scale (ADL). AA, auricular acupuncture; ACUP, acupuncture; AE, Aerobic exercise; CFT, Cognitive function training; CT, Conventional treatment; EMGBFB, electromyographic biofeedback; EA, electroacupuncture; FNS, Fastigial nucleus stimulation; HBO, hyperbaric oxygen therapy; MB, moxibustion; RT, Rehabilitation training; rTMS, Repetitive Transcranial Magnetic Stimulation.

Figure 3 shows a network graph of different non-pharmacological interventions for VaD. Eighty nine studies (32–78, 80–102, 104–122) (97.80%) involving 7,413 participants (96.81%) evaluated the MMSE in the context of 21 non-pharmacological interventions, forming 7 closed loops, with the largest number of studies concentrating on HBO_CT (21 studies) (Figure 3A). Figure 4 shows the pooled MD values for different nonpharmacological interventions compared to CT and the ranking of different nonpharmacological interventions according to P-score. Sixteen non-pharmacological therapies significantly improved MMSE compared to CT, with MDs (95%CI) ranging from 5.09 (3.82; 6.36) for ACUP_MB_CT to 1.45 (0.53; 2.37) for ACUP (Figure 4A). Ranked by the degree of MMSE improvement, ACUP_MB_CT (P-score = 0.95) was defined as the best, while CT (0.07) was considered the worst (Figure 4A). Table 2 shows the results of the NMA on MMSE. NMA results indicated that ACUP_MB_CT, FNS_CT, ACUP_RT_CT, rTMS_CT, AE_CT, MB_CT, HBO_CT, AA_MB, ACUP_CT, rTMS_ACUP_MB_CT, and EMGBFB_CT showed significant significance compared to many other treatments (more than 2).

**Table 2:** League table of MMSE.

ACUP_MB_CT	.	.	.	.	.	.	.	2.68 (1.06; 4.30)	.	.	.	.	.	.	.	.	.	.	.	4.18 (2.37; 5.99)
0.58 (−1.72; 2.88)	FNS_CT	.	.	.	.	.	.	.	.	.	.	.	.	.	.	.	.	.	.	4.51 (2.59; 6.43)
0.91 (−1.00; 2.81)	0.33 (−2.06; 2.71)	ACUP_RT_CT	.	.	.	.	.	.	.	.	.	2.72 (1.48; 3.96)	.	.	.	.	.	.	.	2.41 (−0.50; 5.32)
1.12 (−0.44; 2.67)	0.54 (−1.58; 2.66)	0.21 (−1.47; 1.89)	rTMS_CT	.	.	.	.	.	.	.	.	.	2.30 (0.33; 4.27)	.	.	.	.	.	.	4.09 (3.17; 5.01)
0.84 (−1.87; 3.55)	0.26 (−2.81; 3.33)	−0.06 (−2.85; 2.72)	−0.27 (−2.83; 2.28)	AE_CT	.	.	.	.	.	.	.	.	.	.	.	.	.	.	.	4.25 (1.86; 6.64)
1.77 (−0.21; 3.75)	1.19 (−1.26; 3.64)	0.86 (−1.22; 2.94)	0.65 (−1.11; 2.42)	0.93 (−1.91; 3.76)	MB_CT	.	.	.	.	.	.	.	.	.	.	.	.	.	.	3.32 (1.80; 4.84)
1.86 (0.48; 3.24)	1.28 (−0.71; 3.27)	0.95 (−0.56; 2.47)	0.74 (−0.30; 1.79)	1.02 (−1.44; 3.47)	0.09 (−1.52; 1.70)	HBO_CT	.	.	.	.	.	.	.	.	.	.	.	2.69 (1.32; 4.06)	.	3.23 (2.71; 3.76)
1.91 (−0.11; 3.94)	1.33 (−1.15; 3.82)	1.01 (−1.12; 3.13)	0.80 (−1.02; 2.61)	1.07 (−1.80; 3.94)	0.14 (−2.05; 2.33)	0.05 (−1.61; 1.72)	AA_MB	.	.	.	.	.	.	.	.	.	2.19 (0.51; 3.87)	.	.	3.37 (1.71; 5.02)
1.95 (0.70; 3.20)	1.37 (−0.68; 3.42)	1.04 (−0.55; 2.64)	0.83 (−0.32; 1.99)	1.11 (−1.40; 3.61)	0.18 (−1.51; 1.86)	0.09 (−0.81; 0.99)	0.04 (−1.70; 1.78)	ACUP_CT	.	.	.	.	.	.	.	.	.	.	.	3.31 (2.54; 4.07)
2.13 (0.04; 4.22)	1.55 (−0.99; 4.09)	1.22 (−0.96; 3.41)	1.01 (−0.87; 2.90)	1.29 (−1.63; 4.20)	0.36 (−1.89; 2.61)	0.27 (−1.47; 2.02)	0.22 (−2.07; 2.51)	0.18 (−1.63; 1.99)	rTMS_ACUP_ MB_CT	.	.	.	.	.	.	.	.	.	.	2.96 (1.30; 4.62)
2.32 (0.64; 4.00)	1.74 (−0.47; 3.95)	1.41 (−0.38; 3.20)	1.20 (−0.21; 2.62)	1.48 (−1.15; 4.11)	0.55 (−1.32; 2.42)	0.46 (−0.75; 1.67)	0.41 (−1.51; 2.33)	0.37 (−0.94; 1.68)	0.19 (−1.80; 2.18)	EMGBFB_CT	.	.	.	.	.	.	.	.	3.33 (0.62; 6.04)	2.77 (1.68; 3.86)
2.90 (1.06; 4.73)	2.32 (−0.01; 4.65)	1.99 (0.05; 3.93)	1.78 (0.18; 3.38)	2.05 (−0.68; 4.79)	1.12 (−0.89; 3.14)	1.04 (−0.39; 2.46)	0.98 (−1.08; 3.04)	0.95 (−0.56; 2.46)	0.77 (−1.36; 2.89)	0.58 (−1.14; 2.29)	CFT_CT	.	.	.	.	.	.	.	.	2.20 (0.88; 3.52)
3.31 (1.69; 4.92)	2.73 (0.56; 4.89)	2.40 (1.25; 3.55)	2.19 (0.85; 3.53)	2.46 (−0.13; 5.06)	1.54 (−0.28; 3.35)	1.45 (0.32; 2.58)	1.39 (−0.48; 3.26)	1.36 (0.12; 2.59)	1.18 (−0.76; 3.11)	0.99 (−0.49; 2.47)	0.41 (−1.25; 2.07)	RT_CT	.	.	.	.	.	.	.	2.02 (0.97; 3.07)
3.33 (1.78; 4.88)	2.75 (0.63; 4.86)	2.42 (0.74; 4.09)	2.21 (1.03; 3.39)	2.48 (−0.07; 5.04)	1.56 (−0.20; 3.32)	1.47 (0.43; 2.50)	1.41 (−0.40; 3.23)	1.38 (0.23; 2.53)	1.20 (−0.69; 3.08)	1.01 (−0.40; 2.41)	0.43 (−1.16; 2.02)	0.02 (−1.32; 1.36)	EA_CT	0.02 (−1.34; 1.38)	.	.	.	.	.	1.88 (0.93; 2.83)
3.59 (2.01; 5.16)	3.01 (0.87; 5.14)	2.68 (0.98; 4.38)	2.47 (1.20; 3.74)	2.74 (0.17; 5.31)	1.81 (0.03; 3.60)	1.73 (0.65; 2.80)	1.67 (−0.16; 3.51)	1.64 (0.45; 2.82)	1.45 (−0.45; 3.36)	1.27 (−0.17; 2.70)	0.69 (−0.93; 2.31)	0.28 (−1.09; 1.65)	0.26 (−0.82; 1.34)	EA	.	.	.	.	.	1.37 (0.38; 2.35)
3.64 (2.07; 5.21)	3.06 (0.93; 5.19)	2.73 (1.04; 4.42)	2.52 (1.24; 3.81)	2.80 (0.23; 5.36)	1.87 (0.09; 3.64)	1.78 (0.72; 2.84)	1.73 (−0.10; 3.55)	1.69 (0.52; 2.86)	1.51 (−0.39; 3.41)	1.32 (−0.11; 2.75)	0.74 (−0.86; 2.35)	0.33 (−1.02; 1.69)	0.31 (−0.96; 1.59)	0.06 (−1.25; 1.36)	ACUP	−0.29 (−2.66; 2.08)	.	.	.	1.45 (0.53; 2.37)
3.83 (1.39; 6.27)	3.25 (0.42; 6.08)	2.92 (0.40; 5.44)	2.71 (0.45; 4.98)	2.99 (−0.18; 6.16)	2.06 (−0.52; 4.63)	1.97 (−0.17; 4.12)	1.92 (−0.69; 4.53)	1.88 (−0.32; 4.08)	1.70 (−0.96; 4.36)	1.51 (−0.84; 3.86)	0.93 (−1.53; 3.40)	0.52 (−1.78; 2.83)	0.50 (−1.76; 2.76)	0.24 (−2.04; 2.52)	0.19 (−1.90; 2.28)	MB	.	.	.	0.84 (−1.46; 3.14)
4.29 (2.43; 6.14)	3.71 (1.36; 6.05)	3.38 (1.42; 5.34)	3.17 (1.54; 4.79)	3.44 (0.69; 6.19)	2.51 (0.48; 4.55)	2.43 (0.97; 3.88)	2.37 (0.78; 3.97)	2.34 (0.80; 3.87)	2.15 (0.01; 4.30)	1.97 (0.23; 3.70)	1.39 (−0.50; 3.28)	0.98 (−0.70; 2.66)	0.96 (−0.66; 2.58)	0.70 (−0.94; 2.34)	0.64 (−0.99; 2.28)	0.46 (−2.03; 2.94)	AA	.	.	0.84 (−0.52; 2.19)
5.08 (3.31; 6.84)	4.50 (2.22; 6.78)	4.17 (2.30; 6.05)	3.96 (2.44; 5.48)	4.24 (1.55; 6.93)	3.31 (1.36; 5.26)	3.22 (2.00; 4.44)	3.17 (1.17; 5.16)	3.13 (1.71; 4.55)	2.95 (0.89; 5.01)	2.76 (1.12; 4.40)	2.18 (0.38; 3.99)	1.77 (0.19; 3.35)	1.75 (0.24; 3.27)	1.49 (−0.05; 3.03)	1.44 (−0.09; 2.97)	1.25 (−1.16; 3.66)	0.79 (−1.03; 2.62)	HBO	.	−0.53 (−1.91; 0.84)
5.65 (2.46; 8.84)	5.07 (1.57; 8.57)	4.74 (1.49; 7.99)	4.53 (1.47; 7.59)	4.81 (1.03; 8.59)	3.88 (0.58; 7.17)	3.79 (0.82; 6.76)	3.74 (0.41; 7.06)	3.70 (0.69; 6.71)	3.52 (0.16; 6.88)	3.33 (0.62; 6.04)	2.75 (−0.45; 5.96)	2.34 (−0.75; 5.43)	2.32 (−0.73; 5.38)	2.06 (−1.00; 5.13)	2.01 (−1.06; 5.07)	1.82 (−1.77; 5.41)	1.36 (−1.86; 4.59)	0.57 (−2.60; 3.74)	EMGBFB	.
5.09 (3.82; 6.36)	4.51 (2.59; 6.43)	4.19 (2.77; 5.61)	3.98 (3.08; 4.88)	4.25 (1.86; 6.64)	3.32 (1.80; 4.84)	3.23 (2.71; 3.76)	3.18 (1.60; 4.76)	3.14 (2.42; 3.87)	2.96 (1.30; 4.62)	2.77 (1.68; 3.86)	2.20 (0.88; 3.52)	1.79 (0.79; 2.79)	1.77 (0.88; 2.65)	1.51 (0.57; 2.44)	1.45 (0.53; 2.37)	1.26 (−0.82; 3.34)	0.81 (−0.55; 2.16)	0.01 (−1.21; 1.24)	−0.56 (−3.48; 2.37)	CT

All results are presented in the form of MD (95% CI). Non-pharmacological interventions are ranked according to the *P*-value from left to right, starting with the best. The results of the network meta-analysis are showed in the lower left part, and results from pairwise comparisons in the upper right half (if available). MMSE, Mini-Mental State Examination; MD, Mean Difference; CI, Credible Interval; ACUP, acupuncture; MB, moxibustion; FNS, Fastigial nucleus stimulation; RT, Rehabilitation training; rTMS, Repetitive Transcranial Magnetic Stimulation; AE, Aerobic exercise; HBO, hyperbaric oxygen therapy; AA, auricular acupuncture; EMGBFB, electromyographic biofeedback; CFT, Cognitive function training; EA, electroacupuncture; CT, Conventional treatment.

#### 3.3.2 ADL

Twenty seven studies (34, 36, 40, 45, 46, 49, 63, 65, 71, 74, 76–80, 88, 92, 94, 97, 103, 104, 109, 110, 113, 121) (29.67%) involving 2,105 participants (27.49%) evaluated the ADL in the context of 12 non-pharmacological therapies, forming a closed loop, with ACUP_CT and HBO_CT vs. CT (10 studies) being the most studied interventions (Figure 3B). Compared to CT, all 12 non-pharmacological therapies significantly improved ADL, with MDs (95%CI) ranging from 17.21 (13.19; 21.23) for ACUP_MB_CT to 3.87 (1.40; 6.35) for CFT_CT (Figure 4B). Ranked by the degree of ADL improvement, ACUP_MB_CT (0.98) was defined as the best, while CT was considered the worst (Figure 4B). Table 3 shows the results of the NMA on ADL. The NMA results indicated that ACUP_MB_CT, ACUP_RT_CT, rTMS_CT, ACUP_CT, AE_CT, EMGBFB_CT, and HBO_CT showed significant significance compared to many other treatments (more than 2).

**Table 3:** League table of ADL.

ACUP_MB_CT	.	.	5.98 (2.48; 9.48)	.	.	.	.	.	.	.	.	.
2.88 (−4.25; 10.02)	ACUP_RT_CT	.	.	.	.	.	.	7.13 (2.19; 12.07)	.	.	.	.
5.38 (0.92; 9.83)	2.49 (−3.71; 8.69)	rTMS_CT	.	.	.	.	.	.	.	8.20 (5.37; 11.03)	.	11.83 (9.92; 13.75)
5.98 (2.48; 9.48)	3.10 (−3.12; 9.31)	0.60 (−2.14; 3.35)	ACUP_CT	.	.	.	.	.	.	.	.	11.23 (9.26; 13.19)
6.78 (1.38; 12.18)	3.89 (−3.02; 10.81)	1.40 (−2.68; 5.49)	0.80 (−3.31; 4.91)	AE_CT	.	.	.	.	.	.	.	10.43 (6.82; 14.04)
7.03 (2.52; 11.55)	4.15 (−2.10; 10.40)	1.66 (−1.16; 4.48)	1.05 (−1.79; 3.90)	0.26 (−3.90; 4.41)	EMGBFB_CT	.	.	.	.	.	.	10.17 (8.11; 12.24)
8.26 (3.83; 12.69)	5.38 (−0.81; 11.56)	2.89 (0.21; 5.56)	2.28 (−0.43; 4.99)	1.48 (−2.58; 5.54)	1.23 (−1.55; 4.01)	HBO_CT	.	.	.	.	.	8.95 (7.08; 10.81)
9.66 (2.17; 17.16)	6.78 (−1.87; 15.43)	4.29 (−2.32; 10.90)	3.68 (−2.94; 10.31)	2.89 (−4.40; 10.17)	2.63 (−4.02; 9.28)	1.40 (−5.19; 8.00)	FNS_CT	.	.	.	.	7.54 (1.22; 13.87)
10.01 (4.87; 15.16)	7.13 (2.19; 12.07)	4.64 (0.89; 8.39)	4.03 (0.26; 7.81)	3.24 (−1.60; 8.07)	2.98 (−0.84; 6.80)	1.75 (−1.97; 5.47)	0.35 (−6.75; 7.45)	RT_CT	.	.	.	7.19 (3.97; 10.41)
12.47 (7.15; 17.80)	9.59 (2.73; 16.45)	7.10 (3.11; 11.09)	6.49 (2.48; 10.51)	5.70 (0.67; 10.72)	5.44 (1.38; 9.50)	4.21 (0.24; 8.18)	2.81 (−4.42; 10.04)	2.46 (−2.30; 7.22)	EA	.	.	4.73 (1.23; 8.23)
12.99 (8.19; 17.79)	10.11 (3.65; 16.56)	7.61 (4.98; 10.25)	7.01 (3.73; 10.29)	6.21 (1.75; 10.67)	5.96 (2.61; 9.30)	4.73 (1.50; 7.95)	3.32 (−3.52; 10.17)	2.98 (−1.18; 7.13)	0.52 (−3.86; 4.89)	EA_CT	.	4.80 (1.97; 7.63)
13.33 (8.62; 18.05)	10.45 (4.06; 16.84)	7.96 (4.83; 11.09)	7.35 (4.20; 10.51)	6.56 (2.18; 10.93)	6.30 (3.08; 9.52)	5.07 (1.98; 8.17)	3.67 (−3.12; 10.46)	3.32 (−0.74; 7.38)	0.86 (−3.42; 5.14)	0.34 (−3.26; 3.95)	CFT_CT	3.87 (1.40; 6.35)
17.21 (13.19; 21.23)	14.32 (8.43; 20.22)	11.83 (9.92; 13.75)	11.23 (9.26; 13.19)	10.43 (6.82; 14.04)	10.17 (8.11; 12.24)	8.95 (7.08; 10.81)	7.54 (1.22; 13.87)	7.19 (3.97; 10.41)	4.73 (1.23; 8.23)	4.22 (1.59; 6.85)	3.87 (1.40; 6.35)	CT

All results are presented in the form of MD (95% CI). Non-pharmacological interventions are ranked according to the *P*-value from left to right, starting with the best. The results of the network meta-analysis are showed in the lower left part, and results from pairwise comparisons in the upper right half (if available). ADL, Activities of Daily Living Scale; MD, Mean Difference; CI, Credible Interval; ACUP, acupuncture; MB, moxibustion; RT, Rehabilitation training; rTMS, Repetitive Transcranial Magnetic Stimulation; AE, Aerobic exercise; EMGBFB, electromyographic biofeedback; HBO, hyperbaric oxygen therapy; FNS, Fastigial nucleus stimulation; CFT, Cognitive function training; EA, electroacupuncture; CT, Conventional treatment.

### 3.4 Cluster analysis

**Figure 5:**
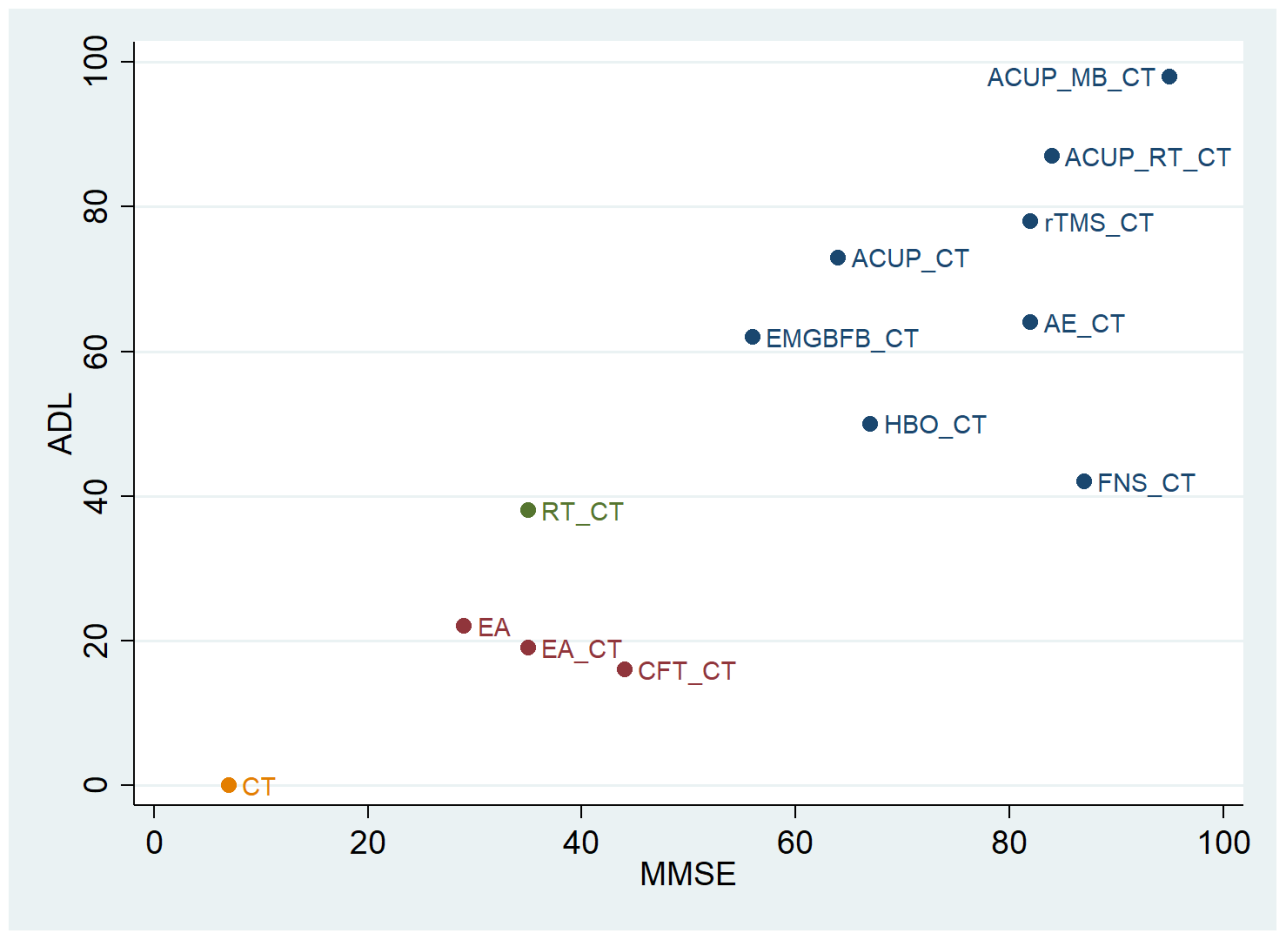
Cluster analysis plot of main outcomes. ACUP, acupuncture; MB, moxibustion; RT, Rehabilitation training; rTMS, Repetitive Transcranial Magnetic Stimulation; AE, Aerobic exercise; EMGBFB, electromyographic biofeedback; HBO, hyperbaric oxygen therapy; FNS, Fastigial nucleus stimulation; CFT, Cognitive function training; EA, electroacupuncture; CT, Conventional treatment; MMSE, Mini-Mental State Examination; ADL, Activities of Daily Living Scale.

Figure 5 shows the results of the cluster analysis. We conducted cluster analysis on the MMSE and ADL outcomes in this study to identify interventions that were effective for improving both outcomes. The cluster analysis of MMSE and ADL showed that ACUP_MB_CT, ACUP_RT_CT, rTMS_CT, AE_CT, and ACUP_CT were located in the upper right corner, indicating relatively better performance.

### 3.5 Adverse reactions

Among the 18 studies (34, 37, 46, 47, 52, 53, 66, 70, 82, 87, 93, 95, 98, 100, 101, 104, 106, 110) included, adverse reactions were reported in all cases. Specifically, 14 studies (34, 37, 46, 47, 66, 70, 87, 93, 95, 98, 100, 104, 106, 110) documented various adverse reactions, primarily characterized by symptoms such as nausea, abdominal pain, and dizziness, which exhibited mild intensity and did not disrupt the treatment procedures. These adverse reactions were predominantly noted in research studies linked to rTMS_CT, ACUP_CT, MB_CT, ACUP_MB_CT, HBO_CT, EMGBFB_CT, and FNS_CT. Additional details regarding the specific adverse reactions were accessed in Supplementary Appendix 6.

### 3.6 The small sample effect and publication bias

**Figure 6:**
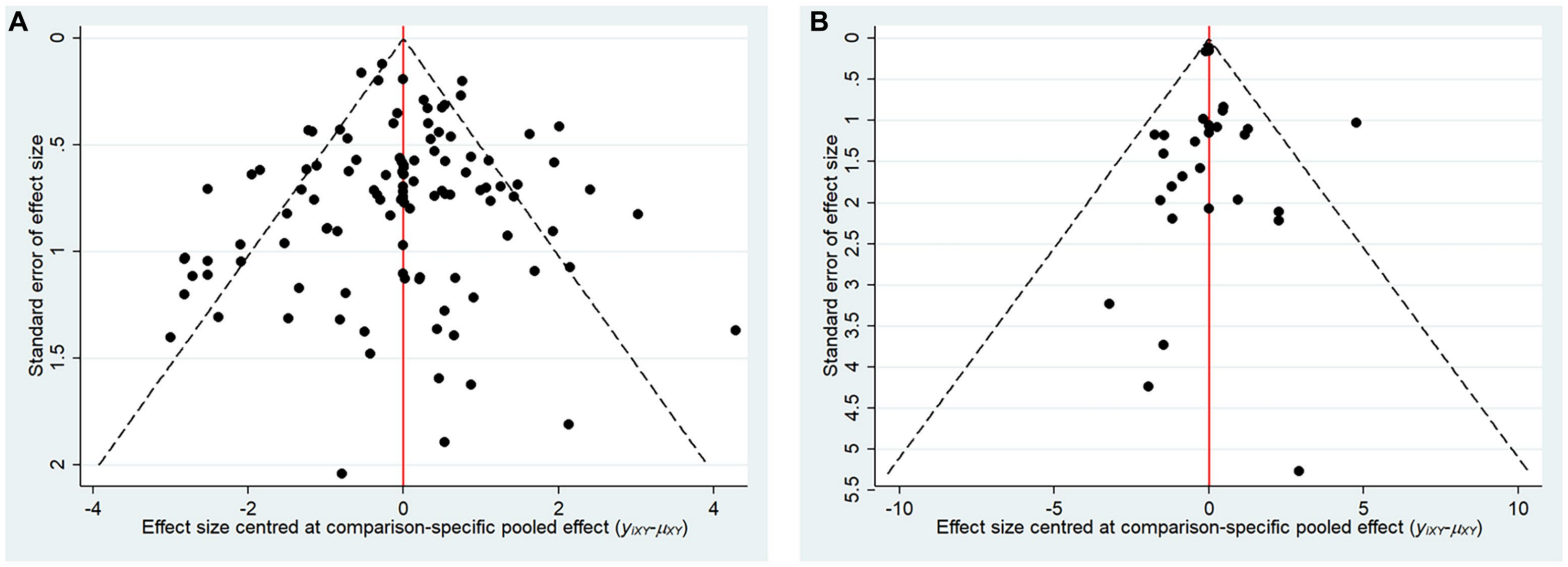
Comparison adjusted funnel plots for main outcomes. **(A)** Mini-mental state examination (MMSE), **(B)** Activities of Daily Living Scale (ADL).

The comparative adjusted funnel plot results demonstrate that the funnel plots of MMSE and ADL are generally symmetrical (Figure 6). The study findings are symmetrically distributed around the midline at the top, indicating a lower likelihood of small sample effects.

### 3.7 Heterogeneity and certainty of evidence

Table 4 shows the results of the assessment of heterogeneity and inconsistency. The heterogeneity results varied from moderate to high, with a global I^2^ of 74.3% for MMSE and 54.8% for ADL. Moreover, none of the global inconsistencies in the outcome measures were statistically significant, and the local inconsistency assessed by the SIDE test did not demonstrate substantial disparities (Table 4; Supplementary Appendix 7). Furthermore, the level of evidence grading for each outcome measure varied from very low to high certainty, suggesting an overall low quality (Supplementary Appendix 10).

**Table 4:** Evaluation of heterogeneity and inconsistency.

Outcomes	Number of studies	Heterogeneity					Heterogeneity assessment	SIDE splitting number of inconsistent comparisons out of total	Percentage of inconsistent comparisons out of total	The design-by-treatment test			
		τ^2^	Q	df	*P*	I^2^				Q	df	τ^2^	*p*-value
MMSE	89	0.9763	310.89	80	< 0.0001	74.3%	Moderate to high	0	0%	15.54	9	0.7332	0.0770
ADL	27	2.0683	35.42	16	0.0035	54.8%	Moderate to high	0	0%	1.74	1	1.4219	0.1875

MMSE, Mini-Mental State Examination; ADL, Activities of Daily Living Scale.

### 3.8 Network meta-regression and sensitivity analysis

Table 5 shows the results of the meta-regression. We identified sources of heterogeneity through meta-regression and sensitivity analysis, with a primary focus on baseline information, treatment duration, treatment frequency, and other covariates. We found that sample, duration and time were the sources of heterogeneity in this study. Additionally, we compared the adjusted results with the original outcomes through the centralization of values for various covariates according to the model. The MDs of non-pharmacological interventions types did not change significantly, and the hierarchy largely remained consistent compared to the unadjusted model (Supplementary Appendix 8). Refined sensitivity analyses, which excluded studies with high risk of bias or focused on studies with treatment duration between 4 and 16 weeks, did not significantly influence the MDs and rankings (Supplementary Appendix 9). In conclusion, the results of our study were stable.

**Table 5:** Network meta-regression.

Outcomes	Shared beta (median and 95% CrI)							
	Year	Sample	Male	Age	Duration	Period	Frequency	Time
MMSE	0.07 (−0.64; 0.79)	−0.81 (−1.44; −0.17)*	−0.38 (−1.06; 0.26)	−0.38 (−1.06; 0.26)	0.01 (−0.52; 0.56)	−0.05 (−0.76; 0.65)	0.18 (−0.84; 1.24)	−1.03 (−2.00; −0,06) *
ADL	−2.34 (−5.51; 1.13)	−2.18 (−5.24; 1.70)	−0.99 (−4.49; 2.63)	0.32 (−1.95; 2.49)	−1.96 (−3.32; −0.31)*	1.22 (−1.77; 4.25)	−0.10 (−5.95; 6.15)	2.96 (−0.52; 6.20)

CI, Credible Interval; *, significant influence factors, 95% CI does not contain zero; MMSE, Mini-Mental State Examination; ADL, Activities of Daily Living Scale.

## 4 Discussion

Our study included 91 studies on non-pharmacological therapies and 2 outcome indicators. We conducted a comprehensive evaluation of the effectiveness of various non-pharmacological therapies in managing VaD through NMA. We found that the majority of non-pharmacological therapies employed as complementary treatments for VaD were statistically significant. The NMA results indicated that (1) acupuncture-related therapies achieved high rankings in both MMSE and ADL assessments, including ACUP_MB_CT, ACUP_RT_CT, and ACUP_CT; (2) 16 non-pharmacological therapies significantly improved the MMSE, with ACUP_MB_CT showing the best effect, and FNS_CT, ACUP_RT_CT, rTMS_CT, AE_CT achieving similarly high *p*-values; (3) 12 non-pharmacological therapies significantly improved the ADL, with ACUP_MB_CT showing the best effect; (4) rTMS_CT and AE_CT also showed significant improvements in both MMSE and ADL.

In addition, our study revealed that all non-pharmacological therapies combined with conventional treatment significantly outperformed conventional treatment in improving ADL. Furthermore, in terms of enhancing MMSE, most non-pharmacological therapies combined with conventional treatment were superior to conventional treatment; however, MB, AA, HBO, and EMGBFB showed no significant difference compared to conventional treatment in this aspect. This lack of significance may be attributed to the limited number of studies incorporating MMSE or the lower baseline MMSE scores. Notably, the analysis indicated that rTMS_ACUP_MB_CT did not improve MMSE as effectively as utilizing rTMS_CT, ACUP_CT, or MB_CT alone. Given the quality, quantity, and baselines of the included RCTs, more research is necessary to validate this observation. Importantly, the findings suggest that non-pharmacological therapies did not significantly increase the incidence of adverse reactions based on the outcomes reported in the included studies.

As clinical trials and animal experiments progress, the mechanisms of non-pharmacological therapies for treating VaD are gradually being unveiled. Acupuncture-related treatments such as ACUP_MB_CT, ACUP_RT_CT, and ACUP_CT have demonstrated promising outcomes in enhancing MMSE and ADL scores. This indicates that acupuncture is a clinically valuable approach, and its synergistic effects can be enhanced when combined with RT or MB therapies. Acupuncture is a unique traditional Chinese therapy known for its multi-target, multi-faceted, and holistic approach. Recent research indicates that acupuncture holds promise in reducing peripheral inflammation and immune abnormalities by targeting inflammatory mediators like Interleukin-1 beta (IL-1β), IL-2, and Tumor Necrosis Factor-alpha (TNF-α) (123), consequently alleviating neural inflammation and ameliorating cognitive impairments (124). Furthermore, acupuncture exhibits a direct mechanism for enhancing cognitive function affected by neural inflammation through the inhibition of the microRNA-93 (miR-93)-mediated Toll-like receptor 4 (TLR4) signaling pathway (125, 126). Past investigations have underscored the pivotal role of TLR4 in mediating inflammatory responses of immune cells within the central nervous system (127), directly linking it to brain damage and neuronal death observed in cases of cerebral ischemia and stroke (128). Furthermore, according to an MRI-based imaging study, acupuncture has been shown to enhance cerebral white matter perfusion and maintain myelin integrity, subsequently safeguarding cognitive function (129). Notably, acupuncture can boost synaptic plasticity (130), acting as the biological foundation for learning and memory processes (131). According to the included studies, we found that Baihui (DU20), Shenting (DU24), Si Shencong (EX-HN1), and Zu Sanli (ST36) were the most commonly used acupoints for treating vascular dementia. A functional brain imaging study showed that adding DU20 enhanced cognitive function by enhancing the medial temporal lobe system, thalamus system, and prefrontal cortex system (132). Furthermore, acupuncture discovered by Yang et al. reduced the inhibitory effects of the 2-vessel occlusion model on hippocampal long-term potentiation, thereby protecting synaptic plasticity. Among the acupoints, DU20 and ST36 exhibited the best therapeutic effect (133). Additionally, needling DU20 and DU24 augmented the density of dendritic spines in the hippocampus of rats (134). An increase in dendritic spine density is associated with improvements in cognitive processes such as learning and memory (135). Within clinical settings, acupuncture has demonstrated promising outcomes in improving limb movement, swallowing function, and language skills (136–138), thereby playing a crucial role in rejuvenating patients’ everyday life capabilities. As a passive non-pharmacological intervention, the integration of acupuncture with active rehabilitation exercises can enhance the restoration of motor and daily life functions (139). Consequently, these findings may elucidate the favorable rankings of acupuncture-related interventions in enhancing MMSE and ADL within this NMA.

ACUP_MB_CT ranks the best according to the P-score in improving both MMSE and ADL. Moxibustion enhances neurogenesis and angiogenesis in rats by upregulating the expression of nestin, doublecortin, and CD34 in the hippocampus (140). Furthermore, It further improves cognitive function in rats with VaD by attenuating hippocampal neuronal apoptosis (141). We speculate that the combined effect of acupuncture and moxibustion makes ACUP_MB_CT the most effective in improving MMSE and ADL.

The clinical value of FNS_CT in enhancing MMSE performance is notable; however, its effectiveness in improving ADL is relatively limited. In upcoming clinical trials, the consideration of integrating other treatment modalities to address this limitation is warranted. FNS, as a non-invasive electrical stimulation therapy, plays a crucial role in enhancing cerebral blood flow and exerting neuroprotective effects by inhibiting excitotoxicity, neuroinflammation, and cell apoptosis (142). Significantly, animal experiments have demonstrated that FNS can downregulate NLRP3 mRNA and protein expression, thereby inhibiting autophagy processes and suppressing the expression of caspase 1, IL-1β, and IL-18. This leads to a reduction in neuroinflammation, neuronal apoptosis, and an improvement in cognitive function among patients (143).

One treatment method is unlikely to be the sole best approach for VaD. rTMS_CT and AE_CT also demonstrate good efficacy. This provides more treatment options for healthcare professionals and patients to choose the most suitable approach based on individual circumstances. Studies have shown that rTMS can improve the learning and memory abilities of rats with VaD by upregulating the expression of vascular endothelial growth factor, brain-derived neurotrophic factor, and the NMDAR (144). BDNF is well-known for its important role in repairing and regenerating neural cells, as well as enhancing neural function (145). Additionally, the NMDAR is closely associated with synaptic plasticity within the hippocampal cornu ammonis 1 region (146). Previous research has also found that rTMS can protect hippocampal cholinergic neurons damaged by chronic brain ischemia-hypoxia and restore the activity of the hippocampal cholinergic system (147). These may be reasons why rTMS_CT ranks highly in improving ADL and MMSE. AE regulates the expression of Beclin-1 in the hippocampus, impacting autophagy and apoptosis, and enhancing hippocampal function (148). This exercise also promotes brain blood circulation, increasing cerebral blood flow (149). Furthermore, aerobic exercise enhances metabolism, strengthens muscle training, and improves cardiovascular function, all contributing to improved daily life abilities and cognitive function (150, 151) Based on the analysis presented above and the results of NMA, it is evident that the combination of non-pharmacological therapy with conventional treatment can address VaD through diverse pathways and targets, offering valuable insights for clinicians to select more effective and suitable non-pharmacological interventions.

## 5 Strengths and limitations

To our knowledge, this is the first NMA comparing the effectiveness and safety of different non-pharmacological therapies for VaD. The NMA combines direct and indirect evidence to compare different interventions, thereby enhancing the evidence. It also provides a comprehensive evaluation and ranking of various interventions to identify their strengths and weaknesses. We searched 7 databases, including 3 English databases and 4 Chinese databases, to increase the breadth and diversity of studies. We assessed the overall heterogeneity and used network meta-regression to explore potential sources of heterogeneity. For inconsistency, we performed both global inconsistency tests and localized inconsistency tests using the SIDE test, which allows for clear assessment of significant differences between each node comparison. We demonstrated the stability of our results through network meta-regression and sensitivity analysis.

Our research also has certain limitations: (1) There may be certain methodological limitations. Out of the included studies, 41 (45.05%) described specific randomization methods, 37 (40.66%) only mentioned randomization, and 13 (14.29%) grouped participants based on visit order or did not mention it. Moreover, only 3 studies mentioned blinding. (2) The sample size ranged from 33 to 234, resulting in moderate to high heterogeneity in the studies. However, the funnel plot did not reveal any significant small sample effects, suggesting the possible presence of individual studies with larger sample sizes. (3) The included studies had a wide range of disease duration (approximately 1–85 months). Previous studies have found that overall, VaD worsens with time. Our meta-regression also identified disease duration as a source of heterogeneity in this study. (4) The included studies were predominantly conducted in China, which may affect the generalizability of the results. (5) Most literature reported adverse reactions descriptively, and there were safety variations among different interventions, so we only conducted descriptive analysis. (6) Our treatment methods were classified based on the descriptions in the included literature, resulting in 21 different treatment methods, which may introduce some bias to the results. For example, rTMS_ACUP_MB_CT is a combination of multiple treatment methods, while EA does not include conventional treatment. Therefore, more research is needed in the future to support our findings.

## 6 Conclusion

This study, which utilizes the NMA method, aims to compare the efficacy and safety of non-pharmacological therapies in conjunction with conventional treatments for VaD. In summary, following pairwise comparisons of different treatment methods and utilizing P-score ranking and cluster analysis, ACUP_MB_CT emerges as the most effective intervention for enhancing VaD, as indicated by improvements in both MMSE and ADL. Moreover, ACUP_RT_CT, rTMS_CT, ACUP_CT, FNS_CT, and AE_CT exhibit significant efficacy across various domains. As for safety, the descriptive results reveal no instances of serious adverse reactions, and it is noted that non-pharmacological therapies do not lead to a significant increase in adverse reactions, thereby indicating a certain degree of safety. It is anticipated that the outcomes of this study will assist clinicians, caregivers, and patients in making informed decisions.

## Data availability statement

The datasets presented in this study can be found in online repositories. The names of the repository/repositories and accession number(s) can be found in the article/Supplementary material.

## Author contributions

YY: Writing – review & editing, Writing – original draft, Software, Methodology, Formal analysis, Data curation, Conceptualization. YQ: Writing – review & editing, Validation, Supervision. SL: Writing – review & editing, Software, Methodology, Investigation. GZ: Writing – review & editing, Software, Investigation, Data curation, Conceptualization. YR: Writing – review & editing, Software, Resources, Project administration, Methodology. ML: Writing – review & editing, Visualization, Validation, Supervision, Funding acquisition.
