# Supplementary material for: Comparative efficacy and safety of non-pharmacological interventions as adjunctive treatment for vascular dementia: a systematic review and network meta-analysis
Source: Front Neurol. 2024 Jul 12;15:1397088. doi: 10.3389/fneur.2024.1397088 (PMC11272661; doi:10.3389/fneur.2024.1397088)
Supplement: Supplementary file 1 [file Data_Sheet_1.DOCX]

**Appendix**

**Contents:**

[Appendix 1: Search Strategy 2](#_Toc160663429)

[1.1 Database: PubMed <inception to January 31, 2024> 2](#_Toc160663430)

[1.2 Database: Embase < inception to January 31, 2024> 4](#_Toc160663431)

[1.3 Database: Cochrane < inception to January 31, 2024> 6](#_Toc160663432)

[Appendix 2: Definitions of different non-pharmacological therapies and conventional treatment 9](#_Toc160663433)

[Appendix 3: Outcome 11](#_Toc160663434)

[3.1 Primary outcomes：MMSE 11](#_Toc160663435)

[3.2 Secondary outcomes：ADL 11](#_Toc160663436)

[3.3 Adverse reactions 11](#_Toc160663437)

[Appendix 4: Characteristics of studies and subjects included in the review 13](#_Toc160663438)

[Appendix 5: Risk of Bias 22](#_Toc160663439)

[Appendix 6: Adverse reactions 25](#_Toc160663440)

[Appendix 7: Evaluation of heterogeneity and inconsistency 26](#_Toc160663441)

[7.1 Heterogeneity 26](#_Toc160663442)

[7.2 Inconsistency (global inconsistency and SIDE splitting results) 26](#_Toc160663443)

[Appendix 8: Network Meta-Regression 38](#_Toc160663444)

[8.1 MMSE 39](#_Toc160663445)

[8.2 ADL 47](#_Toc160663446)

[Appendix 9：Sensitivity analyses 55](#_Toc160663447)

[9.1 treatment period 4-16 weeks 55](#_Toc160663448)

[9.2 Exclude high-risk research. 57](#_Toc160663449)

[Appendix 10: Grading the evidence for outcomes of the network meta-analysis using CINeMA 59](#_Toc160663450)

[10.1 MMSE 59](#_Toc160663451)

[10.2 ADL 73](#_Toc160663452)

[Appendix 11: PRISMA guideline 79](#_Toc160663453)

# Appendix 1: Search Strategy

## 1.1 Database: PubMed <inception to January 31, 2024>

| #1 | "Dementia, Vascular"[Mesh] | 7642 |
| --- | --- | --- |
| #2 | Vascular Dementias[Title/Abstract] OR Vascular Dementia[Title/Abstract] OR Vascular Dementia, Acute Onset[Title/Abstract] OR Acute Onset Vascular Dementia[Title/Abstract] OR Subcortical Vascular Dementia[Title/Abstract] OR Dementia, Subcortical Vascular[Title/Abstract] OR Dementias, Subcortical Vascular[Title/Abstract] OR Subcortical Vascular Dementias[Title/Abstract] OR Vascular Dementia, Subcortical[Title/Abstract] OR Vascular Dementias, Subcortical[Title/Abstract] OR Arteriosclerotic Dementia[Title/Abstract] OR Arteriosclerotic Dementias[Title/Abstract] OR Dementia, Arteriosclerotic[Title/Abstract] OR Dementias, Arteriosclerotic[Title/Abstract] OR Binswanger Disease[Title/Abstract] OR Disease, Binswanger[Title/Abstract] OR Chronic Progressive Subcortical Encephalopathy[Title/Abstract] OR Binswanger Encephalopathy[Title/Abstract] OR Leukoencephalopathy, Subcortical[Title/Abstract] OR Leukoencephalopathies, Subcortical[Title/Abstract] OR Subcortical Leukoencephalopathies[Title/Abstract] OR Encephalopathy, Subcortical Arteriosclerotic[Title/Abstract] OR Binswanger's Disease[Title/Abstract] OR Binswangers Disease[Title/Abstract] OR Disease, Binswanger's[Title/Abstract] OR Encephalopathy, Subcortical, Chronic Progressive[Title/Abstract] OR Subcortical Encephalopathy, Chronic Progressive[Title/Abstract] OR Subcortical Leukoencephalopathy[Title/Abstract] OR Subcortical Arteriosclerotic Encephalopathy[Title/Abstract] OR Arteriosclerotic Encephalopathy, Subcortical[Title/Abstract] OR Arteriosclerotic Encephalopathies, Subcortical[Title/Abstract] OR Encephalopathies, Subcortical Arteriosclerotic[Title/Abstract] OR Subcortical Arteriosclerotic Encephalopathies[Title/Abstract] OR Encephalopathy, Binswanger's[Title/Abstract] OR Binswanger's Encephalopathy[Title/Abstract] OR Encephalopathy, Binswangers[Title/Abstract] OR Encephalopathy, Binswanger[Title/Abstract] OR Encephalopathy, Chronic Progressive Subcortical[Title/Abstract] OR dementia, multi-infarct[Title/Abstract] OR dementia, multiinfarct[Title/Abstract] OR dementia, vascular[Title/Abstract] OR lacunar dementia[Title/Abstract] OR multi-infarct dementia[Title/Abstract] OR multi-infarction dementia[Title/Abstract] OR multiinfarction dementia[Title/Abstract] OR vascular dementia[Title/Abstract] OR multiinfarct dementia[Title/Abstract] | 11963 |
| #3 | #1 OR #2 | 14156 |
| #4 | "Complementary Therapies"[Mesh] | 249235 |
| #5 | Therapies, Complementary[Title/Abstract] OR Therapy, Complementary[Title/Abstract] OR Complementary Medicine[Title/Abstract] OR Medicine, Complementary[Title/Abstract] OR Alternative Medicine[Title/Abstract] OR Medicine, Alternative[Title/Abstract] OR Alternative Therapies[Title/Abstract] OR Therapies, Alternative[Title/Abstract] OR Therapy, Alternative[Title/Abstract] | 21757 |
| #6 | non-pharmacological interventions[Title/Abstract] OR non-pharmacological Therapy[Title/Abstract] OR non-drug Therapy[Title/Abstract] | 786 |
| #7 | "Transcranial Magnetic Stimulation"[Mesh] | 15451 |
| #8 | TMS[Title/Abstract] OR Magnetic Stimulation, Transcranial[Title/Abstract] OR Magnetic Stimulations, Transcranial[Title/Abstract] OR Stimulation, Transcranial Magnetic[Title/Abstract] OR Stimulations, Transcranial Magnetic[Title/Abstract] OR Transcranial Magnetic Stimulations[Title/Abstract] OR Transcranial Magnetic Stimulation, Single Pulse[Title/Abstract] OR Transcranial Magnetic Stimulation, Paired Pulse[Title/Abstract] OR Transcranial Magnetic Stimulation, Repetitive[Title/Abstract] | 26622 |
| #9 | "Acupuncture Therapy"[Mesh] | 29984 |
| #10 | Acupuncture Treatment[Title/Abstract] OR Acupuncture Treatments[Title/Abstract] OR Treatment, Acupuncture[Title/Abstract] OR Therapy, Acupuncture[Title/Abstract] OR Pharmacoacupuncture Treatment[Title/Abstract] OR Treatment, Pharmacoacupuncture[Title/Abstract] OR Pharmacoacupuncture Therapy[Title/Abstract] OR Therapy, Pharmacoacupuncture[Title/Abstract] OR Acupotomy[Title/Abstract] OR Acupotomies[Title/Abstract] | 4278 |
| #11 | "Electroacupuncture"[Mesh] | 5148 |
| #12 | "Rehabilitation"[Mesh] | 359755 |
| #13 | Habilitation[Title/Abstract] OR Rehabilitation training[Title/Abstract] | 3439 |
| #14 | Cognitive function training[Title/Abstract] | 19 |
| #15 | "Hyperbaric Oxygenation"[Mesh] | 12793 |
| #16 | Hyperbaric Oxygenations[Title/Abstract] OR Oxygenations, Hyperbaric[Title/Abstract] OR Hyperbaric Oxygen Therapy[Title/Abstract] OR Hyperbaric Oxygen Therapies[Title/Abstract] OR Oxygen Therapies, Hyperbaric[Title/Abstract] OR Oxygen Therapy, Hyperbaric[Title/Abstract] OR Therapies, Hyperbaric Oxygen[Title/Abstract] OR Therapy, Hyperbaric Oxygen[Title/Abstract] OR Oxygenation, Hyperbaric[Title/Abstract] | 13052 |
| #17 | Fastigial nucleus stimulation[Title/Abstract] OR FNS[Title/Abstract] | 1242 |
| #18 | electromyographic biofeedback[Title/Abstract] OR EMGBFB[Title/Abstract] | 278 |
| #19 | "Moxibustion"[Mesh] | 2859 |
| #20 | "Acupuncture, Ear"[Mesh] | 513 |
| #21 | Acupunctures, Ear[Title/Abstract] OR Ear Acupunctures[Title/Abstract] OR Auricular Acupuncture[Title/Abstract] OR Ear Acupuncture[Title/Abstract] OR Acupuncture, Auricular[Title/Abstract] OR Acupunctures, Auricular[Title/Abstract] OR Auricular Acupunctures[Title/Abstract] | 1616 |
| #22 | "Exercise"[Mesh] | 253614 |
| #23 | Exercises[Title/Abstract] OR Physical Activity[Title/Abstract] OR Activities, Physical[Title/Abstract] OR Activity, Physical[Title/Abstract] OR Physical Activities[Title/Abstract] OR Exercise, Physical[Title/Abstract] OR Exercises, Physical[Title/Abstract] OR Physical Exercise[Title/Abstract] OR Physical Exercises[Title/Abstract] OR Acute Exercise[Title/Abstract] OR Acute Exercises[Title/Abstract] OR Exercise, Acute[Title/Abstract] OR Exercises, Acute[Title/Abstract] OR Exercise, Isometric[Title/Abstract] OR Exercises, Isometric[Title/Abstract] OR Isometric Exercises[Title/Abstract] OR Isometric Exercise[Title/Abstract] OR Exercise, Aerobic[Title/Abstract] OR Aerobic Exercise[Title/Abstract] OR Aerobic Exercises[Title/Abstract] OR Exercises, Aerobic[Title/Abstract] OR Exercise Training[Title/Abstract] OR Exercise Trainings[Title/Abstract] OR Training, Exercise[Title/Abstract] OR Trainings, Exercise[Title/Abstract] | 310092 |
| #24 | #4 or #5 or #6 or #7 or #8 or #9 or #10 or #11 or #12 or #13 or #14 or #15 or #16 or #17 or #18 or #19 or #20 or #21 or #22 or #23 | 1006377 |
| #25 | randomized controlled trial[Publication Type] OR randomized[Title/Abstract] OR placebo[Title/Abstract] | 1049740 |
| #26 | #3 and #24 and #25 | 170 |

## 1.2 Database: Embase < inception to January 31, 2024>

| #1 | 'multiinfarct dementia'/exp | 15,001 |
| --- | --- | --- |
| #2 | 'vascular dementias':ab,ti OR 'vascular dementia, acute onset':ab,ti OR 'acute onset vascular dementia':ab,ti OR 'subcortical vascular dementia':ab,ti OR 'dementia, subcortical vascular':ab,ti OR 'dementias, subcortical vascular':ab,ti OR 'subcortical vascular dementias':ab,ti OR 'vascular dementia, subcortical':ab,ti OR 'vascular dementias, subcortical':ab,ti OR 'arteriosclerotic dementia':ab,ti OR 'arteriosclerotic dementias':ab,ti OR 'dementia, arteriosclerotic':ab,ti OR 'dementias, arteriosclerotic':ab,ti OR 'binswanger disease':ab,ti OR 'disease, binswanger':ab,ti OR 'chronic progressive subcortical encephalopathy':ab,ti OR 'binswanger encephalopathy':ab,ti OR 'leukoencephalopathy, subcortical':ab,ti OR 'leukoencephalopathies, subcortical':ab,ti OR 'subcortical leukoencephalopathies':ab,ti OR 'encephalopathy, subcortical arteriosclerotic':ab,ti OR 'binswangers disease':ab,ti OR 'disease, binswangers':ab,ti OR 'encephalopathy, subcortical, chronic progressive':ab,ti OR 'subcortical encephalopathy, chronic progressive':ab,ti OR 'subcortical leukoencephalopathy':ab,ti OR 'subcortical arteriosclerotic encephalopathy':ab,ti OR 'arteriosclerotic encephalopathy, subcortical':ab,ti OR 'arteriosclerotic encephalopathies, subcortical':ab,ti OR 'encephalopathies, subcortical arteriosclerotic':ab,ti OR 'subcortical arteriosclerotic encephalopathies':ab,ti OR 'binswangers encephalopathy':ab,ti OR 'encephalopathy, binswangers':ab,ti OR 'encephalopathy, binswanger':ab,ti OR 'encephalopathy, chronic progressive subcortical':ab,ti OR 'dementia, multi-infarct':ab,ti OR 'dementia, multiinfarct':ab,ti OR 'dementia, vascular':ab,ti OR 'lacunar dementia':ab,ti OR 'multi-infarct dementia':ab,ti OR 'multi-infarction dementia':ab,ti OR 'multiinfarction dementia':ab,ti OR 'vascular dementia':ab,ti | 12849 |
| #3 | #1 OR #2 | 18,113 |
| #4 | 'alternative medicine'/exp | 79,166 |
| #5 | 'complementary therapies':ab,ti OR 'therapies, complementary':ab,ti OR 'therapy, complementary':ab,ti OR 'complementary medicine':ab,ti OR 'medicine, complementary':ab,ti OR 'medicine, alternative':ab,ti OR 'alternative therapies':ab,ti OR 'therapies, alternative':ab,ti OR 'therapy, alternative':ab,ti | 17527 |
| #6 | 'non-pharmacological therapy':ab,ti OR 'non-pharmacological interventions':ab,ti OR 'non-drug therapy':ab,ti | 5182 |
| #7 | 'transcranial magnetic stimulation'/exp | 33,023 |
| #8 | tms:ab,ti OR 'magnetic stimulation, transcranial':ab,ti OR 'magnetic stimulations, transcranial':ab,ti OR 'stimulation, transcranial magnetic':ab,ti OR 'stimulations, transcranial magnetic':ab,ti OR 'transcranial magnetic stimulations':ab,ti OR 'transcranial magnetic stimulation, single pulse':ab,ti OR 'transcranial magnetic stimulation, paired pulse':ab,ti OR 'transcranial magnetic stimulation, repetitive':ab,ti | 22657 |
| #9 | 'acupuncture'/exp | 59107 |
| #10 | 'acupuncture treatment':ab,ti OR 'acupuncture treatments':ab,ti OR 'treatment, acupuncture':ab,ti OR 'therapy, acupuncture':ab,ti OR 'pharmacoacupuncture treatment':ab,ti OR 'treatment, pharmacoacupuncture':ab,ti OR 'pharmacoacupuncture therapy':ab,ti OR 'therapy, pharmacoacupuncture':ab,ti OR acupotomy:ab,ti OR acupotomies:ab,ti | 5961 |
| #11 | 'electroacupuncture'/exp | 9735 |
| #12 | 'rehabilitation'/exp | 516,026 |
| #13 | 'functional readaptation':ab,ti OR 'medical rehabilitation':ab,ti OR readaption:ab,ti OR readjustment:ab,ti OR 'rehabilitation concept':ab,ti OR 'rehabilitation engineering':ab,ti OR 'rehabilitation potential':ab,ti OR 'rehabilitation process':ab,ti OR 'rehabilitation program':ab,ti OR 'rehabilitation programme':ab,ti OR 'rehabilitation, medical':ab,ti OR 'rehabilitative treatment':ab,ti OR resocialisation:ab,ti OR 'resocialisation therapy':ab,ti OR resocialization:ab,ti OR 'resocialization therapy':ab,ti OR revalidation:ab,ti OR rehabilitation:ab,ti | 300829 |
| #14 | 'cognitive function training':ab,ti | 29 |
| #15 | 'hyperbaric oxygen therapy'/exp | 21,422 |
| #16 | 'hbo therapy':ab,ti OR 'high pressure oxygen':ab,ti OR 'high tension o2':ab,ti OR 'high tension oxygen':ab,ti OR 'hyperbaric medicine':ab,ti OR 'hyperbaric o2':ab,ti OR 'hyperbaric oxygen':ab,ti OR 'hyperbaric oxygen treatment':ab,ti OR 'hyperbaric oxygenation':ab,ti OR 'hyperbaric oxygenisation':ab,ti OR 'hyperbaric oxygenization':ab,ti OR 'hyperbaric therapy':ab,ti OR 'oxygen, hyperbaric':ab,ti OR 'hyperbaric oxygen therapy':ab,ti | 14938 |
| #17 | 'fastigial nucleus stimulation':ab,ti OR fns:ab,ti | 1,564 |
| #18 | 'electromyographic biofeedback'/exp | 13 |
| #19 | emgbfb:ab,ti | 28 |
| #20 | 'moxibustion'/exp | 4,875 |
| #21 | 'auricular acupuncture'/exp | 873 |
| #22 | 'acupunctures, ear':ab,ti OR 'ear acupunctures':ab,ti OR 'auricular acupuncture':ab,ti OR 'ear acupuncture':ab,ti OR 'acupuncture, auricular':ab,ti OR 'acupunctures, auricular':ab,ti OR 'auricular acupunctures':ab,ti | 1062 |
| #23 | 'aerobic exercise'/exp | 24133 |
| #24 | 'embase: aerobic dance':ab,ti OR 'aerobic dancing':ab,ti OR aerobics:ab,ti OR 'aerobics exercise':ab,ti OR 'dancing, aerobic':ab,ti OR 'exercise, aerobic':ab,ti OR 'low impact aerobic exercise':ab,ti OR 'low impact aerobics':ab,ti OR 'step aerobics':ab,ti OR 'aerobic exercise':ab,ti | 18678 |
| #25 | #4 OR #5 OR #6 OR #7 OR #8 OR #9 OR #10 OR #11 OR #12 OR #13 OR #14 OR #15 OR #16 OR #17 OR #18 OR #19 OR #20 OR #21 OR #22 OR #23 OR #24 | 890511 |
| #26 | 'randomized controlled trial':ab,ti OR randomized:ab,ti OR placebo:ab,ti | 1,175,282 |
| #27 | #3 AND #25 AND #26 | 140 |

## 1.3 Database: Cochrane < inception to January 31, 2024>

| #1 | MeSH descriptor: [Dementia, Vascular] explode all trees | 459 |
| --- | --- | --- |
| #2 | (Vascular Dementias or Vascular Dementia, Acute Onset or Acute Onset Vascular Dementia or Subcortical Vascular Dementia or Dementia, Subcortical Vascular or Dementias, Subcortical Vascular or Subcortical Vascular Dementias or Vascular Dementia, Subcortical or Vascular Dementias, Subcortical or Arteriosclerotic Dementia or Arteriosclerotic Dementias or Dementia, Arteriosclerotic or Dementias, Arteriosclerotic or Binswanger Disease or Disease, Binswanger or Chronic Progressive Subcortical Encephalopathy or Binswanger Encephalopathy or Leukoencephalopathy, Subcortical or Leukoencephalopathies, Subcortical or Subcortical Leukoencephalopathies or Encephalopathy, Subcortical Arteriosclerotic or Binswanger's Disease or Binswangers Disease or Disease, Binswanger's or Encephalopathy, Subcortical, Chronic Progressive or Subcortical Encephalopathy, Chronic Progressive or Subcortical Leukoencephalopathy or Subcortical Arteriosclerotic Encephalopathy or Arteriosclerotic Encephalopathy, Subcortical or Arteriosclerotic Encephalopathies, Subcortical or Encephalopathies, Subcortical Arteriosclerotic or Subcortical Arteriosclerotic Encephalopathies or Encephalopathy, Binswanger's or Binswanger's Encephalopathy or Encephalopathy, Binswangers or Encephalopathy, Binswanger or Encephalopathy, Chronic Progressive Subcortical or dementia, multi-infarct or dementia, multiinfarct or dementia, vascular or lacunar dementia or multi-infarct dementia or multi-infarction dementia or multiinfarction dementia or vascular dementia or multiinfarct dementia):ti,ab,kw | 2049 |
| #3 | #1 or #2 | 2051 |
| #4 | MeSH descriptor: [Complementary Therapies] explode all trees | 28911 |
| #5 | (Therapies, Complementary or Therapy, Complementary or Complementary Medicine or Medicine, Complementary or Alternative Medicine or Medicine, Alternative or Alternative Therapies or Therapies, Alternative or Therapy, Alternative):ti,ab,kw | 46517 |
| #6 | (non-pharmacological Therapy or non-pharmacological interventions or non-drug Therapy):ti,ab,kw | 5603 |
| #7 | MeSH descriptor: [Transcranial Magnetic Stimulation] explode all trees | 2568 |
| #8 | (TMS or Magnetic Stimulation, Transcranial or Magnetic Stimulations, Transcranial or Stimulation, Transcranial Magnetic or Stimulations, Transcranial Magnetic or Transcranial Magnetic Stimulations or Transcranial Magnetic Stimulation, Single Pulse or Transcranial Magnetic Stimulation, Paired Pulse or Transcranial Magnetic Stimulation, Repetitive):ti,ab,kw | 9299 |
| #9 | MeSH descriptor: [Acupuncture Therapy] explode all trees | 7020 |
| #10 | (Acupuncture Treatment or Acupuncture Treatments or Treatment, Acupuncture or Therapy, Acupuncture or Pharmacoacupuncture Treatment or Treatment, Pharmacoacupuncture or Pharmacoacupuncture Therapy or Therapy, Pharmacoacupuncture or Acupotomy or Acupotomies):ti,ab,kw | 16076 |
| #11 | MeSH descriptor: [Electroacupuncture] explode all trees | 1156 |
| #12 | MeSH descriptor: [Rehabilitation] explode all trees | 54123 |
| #13 | (functional readaptation or medical rehabilitation or readaption or readjustment or rehabilitation concept or rehabilitation engineering or rehabilitation potential or rehabilitation process or rehabilitation program or rehabilitation programme or rehabilitation, medical or rehabilitative treatment or resocialisation or resocialisation therapy or resocialization or resocialization therapy or revalidation or rehabilitation):ti,ab,kw | 69596 |
| #14 | (Cognitive function training):ti,ab,kw | 11647 |
| #15 | MeSH descriptor: [Hyperbaric Oxygenation] explode all trees | 610 |
| #16 | (Hyperbaric Oxygenations or Oxygenations, Hyperbaric or Hyperbaric Oxygen Therapy or Hyperbaric Oxygen Therapies or Oxygen Therapies, Hyperbaric or Oxygen Therapy, Hyperbaric or Therapies, Hyperbaric Oxygen or Therapy, Hyperbaric Oxygen or Oxygenation, Hyperbaric):ti,ab,kw | 1865 |
| #17 | (Fastigial nucleus stimulation or FNS):ti,ab,kw | 106 |
| #18 | (electromyographic biofeedback or EMGBFB):ti,ab,kw | 349 |
| #19 | MeSH descriptor: [Moxibustion] explode all trees | 671 |
| #20 | (Acupunctures, Ear or Ear Acupunctures or Auricular Acupuncture or Ear Acupuncture or Acupuncture, Auricular or Acupunctures, Auricular or Auricular Acupunctures):ti,ab,kw | 1355 |
| #21 | MeSH descriptor: [Acupuncture, Ear] explode all trees | 262 |
| #22 | (Acupunctures, Ear or Ear Acupunctures or Auricular Acupuncture or Ear Acupuncture or Acupuncture, Auricular or Acupunctures, Auricular or Auricular Acupunctures):ti,ab,kw | 1355 |
| #23 | MeSH descriptor: [Exercise] explode all trees | 38461 |
| #24 | (Exercises or Physical Activity or Activities, Physical or Activity, Physical or Physical Activities or Exercise, Physical or Exercises, Physical or Physical Exercise or Physical Exercises or Acute Exercise or Acute Exercises or Exercise, Acute or Exercises, Acute or Exercise, Isometric or Exercises, Isometric or Isometric Exercises or Isometric Exercise or Exercise, Aerobic or Aerobic Exercise or Aerobic Exercises or Exercises, Aerobic or Exercise Training or Exercise Trainings or Training, Exercise or Trainings, Exercise):ti,ab,kw | 184948 |
| #25 | #4 or #5 or #6 or #7 or #8 or #9 or #10 or #11 or #12 or #13 or #14 or #15 or #16 or #17 or #18 or #19 or #20 or #21 or #22 or #23 or #24 | 326581 |
| #26 | #3 and #25 | 667 |

# Appendix 2: Definitions of different non-pharmacological therapies and conventional treatment

| abbreviation | Full name | Definitions |
| --- | --- | --- |
| rTMS | Repetitive Transcranial Magnetic Stimulation | A method of therapy utilizing a repetitive pulsatile magnetic field directed towards the central nervous system externally, modifying the membrane potential of cortical neurons to elicit currents, influencing brain metabolism and neuronal electrical function, subsequently eliciting a sequence of physiological and biochemical reactions. ^[1]^ |
| ACUP | Acupuncture | The method of treating illnesses by inserting needles along specific pathways or meridians, where the positioning of the needles is adjusted based on the particular ailment being addressed. This approach may be complemented by the application of heat, moxibustion, acupressure, or electric stimulation.^[2]^ In the treatment process, acupuncture therapy often involves acupoints from the entire meridian system, including acupoints on the head and body. Therefore, this study combines head and body acupuncture needles as part of acupuncture therapy without differentiation. |
| MB | Moxibustion | The process involves igniting a tiny, thimble-sized burning cylinder of dried leaves directly on the surface of the skin, specifically targeting an acupuncture point. Typically, these cylinders are filled with mugwort or moxa leaves. ^[3]^ |
| EA | Electroacupuncture | Utilizing electrical impulses transmitted through the needles to activate nerve tissue, this type of acupuncture can be employed for pain relief, numbing, recovery, and managing various medical conditions. |
| RT | Rehabilitation training | Restoration of human functions to the maximum degree possible in a person or persons suffering from disease or injury. In this study, rehabilitation training mainly includes memory training, attention training, orientation impairment training, physical exercise training, psychological training, language exercises, and reasoning and problem-solving training. |
| CFT | Cognitive function training | Cognitive function training is a scientifically designed assessment and systematic training program for human cognitive abilities based on the theory of neuroplasticity.^[4]^  The training in this study mainly targets patients’ cognitive impairments such as memory, attention, and visual-spatial deficits. |
| HBO | Hyperbaric oxygen therapy | Hyperbaric oxygen therapy is a promising field in clinical medicine, offering unique therapeutic benefits and optimistic growth potential. Patients undergo this treatment by entering a chamber where they breathe in highly concentrated oxygen at levels between 85% and 99%, all under elevated pressure conditions. This method significantly boosts blood oxygen levels, surpassing those achieved through normal oxygen intake at regular atmospheric pressure by several to tens times. It proves to be efficient in elevating oxygen levels in the blood and is particularly effective for treating specific acute and chronic hypoxic ailments.^[5]^ |
| EMGBFB | Electromyographic biofeedback | EMGBFT is a therapeutic method that utilizes muscle electrical signals for treatment.^[6]^ Patients learn to self-regulate muscle tension by interpreting EMG values and audiovisual signals displayed by the treatment device. Once mastering self-awareness and control techniques, patients can reduce reliance on the device, improving function and treating diseases. Ultimately, enhancing psychological activities through EMGBFT can alleviate mental symptoms, restore cognitive function, and promote neurotransmitter recovery. |
| FNS | Fastigial nucleus stimulation | Cerebellar fastigial nucleus electrical stimulation (FNS) utilizes digital frequency synthesis technology to non-invasively introduce effective therapeutic currents into the cerebellar fastigial nucleus region through surface electrodes, improving cerebral blood supply and correcting neural conduction disorders. This increases cerebral blood flow, reduces neuronal necrosis in the penumbra, and alleviates brain edema, ultimately enhancing cerebral circulation function. Recent studies have shown that FNS is not only applicable to diseases like stroke but also demonstrates good efficacy in improving cognitive function.^[7]^ |
| AA | Auricular acupuncture | Auricular acupuncture refers to a method of diagnosing and treating diseases by stimulating ear acupoints with short needles or applying Wang Buxing’s bean pellets. In this study, the focus is on using bean pellets to apply pressure to ear acupoints. |
| AE | Aerobic exercise | Aerobic exercise relies on aerobic metabolism for energy, involving major muscle groups, sustained rhythmic movements, and activities like running, swimming, and jump roping. |
| CT | Conventional treatment | Conventional drugs for the treatment of vascular dementia mainly include conventional drugs to improve cognition and conventional drugs to control risk factors , such as donepezil and memantine to improve cognition, and aspirin and other antiplatelet drugs, antihypertensive drugs, hypoglycemic drugs, etc. to control risk factors. |

**Reference:**

[1] Lefaucheur JP, Aleman A, Baeken C, Benninger DH, Brunelin J, Di Lazzaro V, Filipović SR, Grefkes C, Hasan A, Hummel FC, Jääskeläinen SK, Langguth B, Leocani L, Londero A, Nardone R, Nguyen JP, Nyffeler T, Oliveira-Maia AJ, Oliviero A, Padberg F, Palm U, Paulus W, Poulet E, Quartarone A, Rachid F, Rektorová I, Rossi S, Sahlsten H, Schecklmann M, Szekely D, Ziemann U. Evidence-based guidelines on the therapeutic use of repetitive transcranial magnetic stimulation (rTMS): An update (2014-2018). Clin Neurophysiol. 2020 Feb;131(2):474-528. doi: 10.1016/j.clinph.2019.11.002.

[2] Wang F. [Comments on the definition of "acupuncture science"]. Zhongguo Zhen Jiu. 2017 Dec 12;37(12):1333-6. Chinese. doi: 10.13703/j.0255-2930.2017.12.021.

[3] Sun Y, Liu B, He L, Wu X, Liu J. [The current situation of acupuncture definition in international organizations and legislation of some countries]. Zhongguo Zhen Jiu. 2017 Dec 12;37(12):1329-32. Chinese. doi: 10.13703/j.0255-2930.2017.12.020.

[4] Garlick D. Understanding the nature of the general factor of intelligence: the role of individual differences in neural plasticity as an explanatory mechanism. Psychol Rev. 2002 Jan;109(1):116-36. doi: 10.1037/0033-295x.109.1.116.

[5] He, J. Dictionary of Neurology and Psychiatry. China Traditional Chinese Medicine Press. 1998.

[6] Lirio-Romero C, Torres-Lacomba M, Gómez-Blanco A, Acero-Cortés A, Retana-Garrido A, de la Villa-Polo P, Sánchez-Sánchez B. Electromyographic biofeedback improves upper extremity function: a randomized, single-blinded, controlled trial. Physiotherapy. 2021 Mar;110:54-62. doi: 10.1016/j.physio.2020.02.002.

[7] Wang J, Dong WW, Zhang WH, Zheng J, Wang X. Electrical stimulation of cerebellar fastigial nucleus: mechanism of neuroprotection and prospects for clinical application against cerebral ischemia. CNS Neurosci Ther. 2014 Aug;20(8):710-6. doi: 10.1111/cns.12288.

# Appendix 3: Outcome

## 3.1 Primary outcomes：MMSE

The Mini-Mental State Examination (MMSE), developed by Folstein et al. in 1975, is one of the most influential standardized tools for assessing cognitive impairment. It is a simple and practical method for evaluating cognitive function. The scoring method is as follows: 1 point is given for each correct answer, while 0 points are given for errors or unknown answers. Inappropriate responses are scored as 9 points, refusal to answer or lack of understanding as 8 points. When calculating the total score, both 8 and 9 points are considered as 0 points. The maximum score is 30 points. The classification of dementia is related to the individual’s education level, so if an elderly person is illiterate and scores below 17, elementary education and scores below 20, or secondary education and scores below 24, they may be classified as having dementia.

## 3.2 Secondary outcomes：ADL

The Barthel Index is a measure of a patient’s functional status in activities of daily living, with individual scores determined by the assessment of a series of independent behaviors, ranging from 0 to 100 points. Developed and established by Americans Dorother Barthel and Florence Mahoney in 1965, the Barthel Index is a commonly used method of assessing activities of daily living (ADL) in rehabilitation facilities in the United States. It evaluates a patient’s level of independence in daily self-care by assessing their ability to perform 10 basic activities of daily living. These activities include feeding, bathing, dressing, toileting, bowel and bladder control, transfers (bed to chair and back), mobility, stair climbing, and grooming. The Barthel Index score ranges from 0 to 100 points, with higher scores indicating greater independence and less dependence.

## 3.3 Adverse reactions

Adverse reactions refer to harmful responses that occur during the course of treatment for vascular dementia with medications administered according to normal therapy or standard protocols, but are unrelated to the therapeutic purpose. Common adverse reactions to acupuncture include needle fainting, bleeding, and subcutaneous bruising; adverse reactions to transcranial magnetic stimulation may include headaches, dizziness, and seizures.

# Appendix 4: Characteristics of studies and subjects included in the review

This table is used to summarize the covariates included in the study, providing additional information to be used in meta-regression analysis.

Table 4.1 Characteristics of eligible RCTs included in network meta-analysis

| study | year | sample | male | age | duration  (month) | Period (week) | frequency (per week) | time (min/ per session) |
| --- | --- | --- | --- | --- | --- | --- | --- | --- |
| Hao Jianwei 2022 | 2022 | 120 | 61 | 69.1 | 1.8 | 9 | 7 | 20 |
| Ren Yafang 2016 | 2016 | 78 | 47 | 69.01 | 3.23 | 8 | 5 | NA |
| Yang Mingjian 2023 | 2023 | 90 | 44 | 67.06 | 83.76 | 6 | 5 | 16 |
| Li Xiaoying 2016 | 2016 | 80 | 46 | 62.4 | NA | 12 | 7 | 30 |
| Li Hang 2020 | 2020 | 96 | 51 | 65.27 | 20.64 | 4 | 5 | NA |
| Guo Ling 2021 | 2021 | 60 | 35 | 76.17 | NA | 6 | 5 | 25 |
| Pan Hongshan 2018 | 2018 | 80 | 30 | 68.82 | 7.64 | 12 | 8 | 20 |
| Wu Xiaohui 2020 | 2020 | 40 | 24 | 67.25 | 7.25 | 12 | 6 | 60 |
| Cheng Nanfang 2021 | 2021 | 80 | 43 | 70.89 | 0.99 | 4.3 | 7 | 30 |
| Meng Yingchun 2011 | 2011 | 60 | NA | NA | NA | 6 | 7 | 30 |
| Han Hui 2021 | 2021 | 120 | 59 | 65 | 6.5 | 8 | 5 | 30 |
| Qiao Shiguang 2023 | 2023 | 80 | 50 | 68.67 | 24.72 | 3 | 7 | NA |
| Chen Yongjun 2011 | 2011 | 80 | 37 | 64.84 | NA | 4 | 7 | 30 |
| Wang Huazheng 2015 | 2015 | 90 | 66 | 71.38 | 6.72 | 8 | 6 | NA |
| Hu Fengxia 2019 | 2019 | 88 | 47 | 67.5 | 32.16 | 8 | 6 | NA |
| Feng Xiaorong 2019 | 2019 | 94 | 63 | 64.13 | 7 | 8 | 7 | 30 |
| Ye Baoye 2016 | 2016 | 60 | 32 | 58.12 | NA | 4 | 6 | 30 |
| Zhang Shaolei 2022 | 2022 | 90 | 52 | 66.15 | 40.2 | 12 | 6 | 30 |
| Cui Lele 2015 | 2015 | 30 | 32 | 67.5 | 4.99 | 4 | 6 | 40 |
| Yu Tao 2007 | 2007 | 63 | 31 | 70.92 | 15.6 | 12 | 5 | 30 |
| Hu Jingjing 2009 | 2009 | 68 | 43 | 65.45 | NA | 12.86 | 6 | 30 |
| Tan Tao 2017 | 2017 | 60 | 36 | 66.52 | 2.57 | 4 | 7 | 30 |
| Zhang Rui 2010 | 2010 | 60 | 37 | 65.84 | NA | 8 | 7 | 40 |
| Zheng Shenghui 2011 | 2011 | 75 | 43 | 67.91 | 5.05 | 8 | 5 | 30 |
| Gao Qianren 2013 | 2013 | 60 | 27 | 71.92 | 5.32 | 8 | 6 | 30 |
| Yao Weiling 2018 | 2018 | 60 | 34 | 67.77 | NA | 4 | 7 | 20 |
| Xu Lihong 2006 | 2006 | 90 | 58 | 63.1 | NA | 12 | 7 | 30 |
| Liu Lijie 2022 | 2022 | 60 | 33 | 72.23 | NA | 12 | 5 | 45 |
| Yang Xiaoyan 2008 | 2008 | 90 | 41 | 62.6 | NA | 8 | 6 | 30 |
| Peng Xiaohong 2009 | 2009 | 77 | 51 | 65.54 | NA | 6 | 6 | 30 |
| Zhao Ling 2009 | 2009 | 73 | NA | NA | NA | 6 | 5 | 30 |
| Wang Huiming 2007 | 2007 | 64 | 37 | 71.07 | 26.88 | 12 | 6 | 30 |
| Yin Jianquan 2011 | 2011 | 60 | 35 | 62.75 | 19.68 | 12 | 5 | 30 |
| Li Qiaowei 2015 | 2015 | 56 | 31 | 65.75 | NA | 4 | 5 | 30 |
| Wang Minchao 2020 | 2020 | 70 | 37 | 72.9 | 17.55 | 8 | 4 | 60 |
| Sheng Dandan 2017 | 2017 | 60 | 34 | NA | NA | 4 | 6 | 60 |
| Gao Yujie 2018 | 2018 | 80 | 49 | NA | NA | 8 | 1 | NA |
| Luo Benhua 2015 | 2015 | 90 | 40 | 71.85 | 16.68 | 8 | 4 | 30 |
| Fan Weiquan 2019 | 2019 | 100 | 63 | 67.29 | 41.28 | 12 | 5 | 60 |
| Zhao Ruixia 2012 | 2012 | 76 | 41 | 53.2 | 7.65 | 14.5 | 7 | NA |
| Ma Li 2018 | 2018 | 60 | 33 | 67.59 | 20.64 | 8 | 6 | 60 |
| Wang Heng 2021 | 2021 | 100 | 59 | 71.67 | 14.92 | 6 | 5 | NA |
| Wang Xuepeng 2018 | 2018 | 66 | 31 | 69.7 | 27.6 | 12 | 7 | 45 |
| Wang Huaijing 2007 | 2007 | 63 | 49 | 62.27 | 27.72 | 12 | 3 | 90 |
| Sun Peijun 2016 | 2016 | 64 | 39 | 64 | 14.4 | 12 | 5 | 60 |
| Zhai Zhiyong 2015 | 2015 | 56 | 29 | 70 | 36.6 | 24 | 4 | 60 |
| Wu Xiaoling 2018 | 2018 | 86 | 54 | 71.25 | 31.08 | 24 | 7 | 30 |
| Ji Jing 2017 | 2017 | 72 | NA | 51.76 | 2.46 | 8 | 7 | 60 |
| Zhu Kaifeng 2023 | 2023 | 80 | 47 | 69.94 | 28.08 | 12 | NA | NA |
| Qu Yancao 2019 | 2019 | 60 | 40 | 67.9 | NA | 8 | 7 | 30 |
| Zhao Pengxiang 2015 | 2015 | 166 | 81 | 68.5 | 26.4 | 12 | 7 | 60 |
| Liu Li 2015 | 2015 | 96 | 55 | 65.55 | 21 | 6.5 | 7 | 60 |
| Chen Shidong 2011 | 2011 | 82 | 43 | 63.95 | 21 | 7.1 | 7 | 60 |
| Liu Zhiyi 2016 | 2016 | 64 | 37 | 62.5 | 19.2 | 6.5 | 7 | 60 |
| Lei Xiaodan 2016 | 2016 | 60 | 33 | 66.75 | NA | 3 | 7 | 60 |
| Sun Wei 2015 | 2015 | 60 | 34 | 67.5 | 9.6 | 3.4 | 7 | 40 |
| Wang Yongsheng 2013 | 2013 | 80 | 42 | 66.6 | 22.2 | 8 | NA | 60 |
| Wang Shuiping 2009 | 2009 | 64 | 41 | 70.24 | 26.4 | 12 | 6 | 60 |
| Tang Longchong 2013 | 2013 | 200 | 99 | 65.65 | NA |  | 7 | 60 |
| Li Wen 2013 | 2013 | 72 | 41 | 65.25 | 19.8 | 7.1 | 7 | 60 |
| Feng Yufeng 2020 | 2020 | 78 | 49 | 67.52 | 12 | 4 | 7 | 60 |
| Hu Qian 2023 | 2023 | 120 | 57 | 72.79 | 8.07 | 4 | 7 | 80 |
| Wang Yongsheng 2014 | 2014 | 70 | 35 | 68.3 | 22.8 | 8 | 7 | 60 |
| Yang Li 2018 | 2018 | 98 | 47 | 73.1 | 85.2 | 4 | 7 | 60 |
| Li Zicheng 2020 | 2020 | 90 | 49 | 68.66 | NA | 2 | 7 | 80 |
| Wang Songlin 2011 | 2011 | 80 | 42 | 63.95 | 21 | 7.1 | 7 | 60 |
| Xia Xiyuan 2012 | 2012 | 60 | 29 | NA | NA | 4 | 7 | 60 |
| Bao Zhiying 2007 | 2007 | 89 | 40 | 72.1 | NA | 6 | 7 | 60 |
| Wu Yong 2010 | 2010 | 150 | 81 | 63.2 | 6.67 | 3 | 7 | 60 |
| Song Yuhua 2012 | 2012 | 123 | NA | NA | NA | 3 | 7 | 60 |
| Bu Guiwen 2012 | 2012 | 96 | NA | NA | NA | 3 | 7 | 60 |
| Liu Yuqian 2020 | 2020 | 104 | 55 | 64.76 | NA | 4 | 6 | 60 |
| Ran Qian 2018 | 2018 | 78 | 47 | 66.92 | 8.34 | 16 | 5 | 60 |
| Du Huiqing 2015 | 2015 | 84 | 66 | 71 | 23.8 | 24 | 5 | 60 |
| Liu Yalin 2022 | 2022 | 114 | 57 | 69.23 | 6.69 | 4 | 7 | 30 |
| Cai Rui 2018 | 2018 | 94 | 51 | 63.56 | NA | 12 | 5 | 30 |
| Chen Songsheng 2013 | 2013 | 68 | 39 | 72.27 | NA | 3 | 14 | 60 |
| Wu Bingjie 2005 | 2005 | 33 | 24 | 66.8 | 6.6 | 2 | 7 | 40 |
| Dai Jianwu 2008 | 2008 | 46 | 27 | 75.75 | NA | 4 | 14 | 30 |
| Li Ji 2023 | 2023 | 96 | 54 | 61.52 | 10.1 | 8 | 6 | NA |
| Wu Fuyou 2022 | 2022 | 100 | 62 | 60.96 | 19.79 | 4 | NA | NA |
| Li Weimin 2023 | 2023 | 100 | 62 | 60.96 | 20.95 | 4 | 5 | NA |
| Li Weimin 2017 | 2017 | 120 | 68 | NA | NA | 12 | 7 | NA |
| Wang Haiyan 2009 | 2009 | 68 | 45 | 58.23 | 5 | 8 | 7 | NA |
| Shi Guirong 2011 | 2011 | 126 | 85 | 68.64 | NA | 12 | 7 | NA |
| Chen Qi 2009 | 2009 | 163 | 88 | 70.99 | 9.85 | 12 | 7 | NA |
| Kuang Weichuan 2012 | 2012 | 234 | NA | NA | NA | 12 | 6 | NA |
| Wang Xiaoyan 2019 | 2019 | 40 | 25 | 65.75 | 11.78 | 12 | 7 | 20 |
| Wang Linjuan 2014 | 2014 | 60 | 35 | 64.48 | NA | 8 | 6 | 30 |
| Liu Suhua 2016 | 2016 | 124 | 64 | 65.7 | 56.4 | 4 | 14 | 120 |
| Li Rong 2017 | 2017 | 92 | 66 | 65.81 | 14.85 | 8 | NA | NA |

Table 4.2 Intervention measures detailed introduction

| Included studies | Intervention measures |  | Treatment course |
| --- | --- | --- | --- |
|  | E | C |  |
| Hao Jianwei 2022 | rTMS+CT. rTMS stimulates the dorsolateral prefrontal cortex at 5Hz, for 20 minutes each session, once a day. | CT | 21 D*3 |
| Ren Yafang 2016 | rTMS+CT. rTMS stimulates the dorsolateral prefrontal cortex at 120% of motor threshold (MT), with a sequence of 50 pulses, 25 sequences per session, once a day. | CT | 2 M |
| Yang Mingjian 2023 | rTMS+CT. rTMS stimulates the left dorsolateral prefrontal cortex at 20Hz, 80% of MT, for 16 minutes each session, once a day. | CT | 6 W |
| Li Xiaoying 2016 | rTMS+CT. rTMS for 30 minutes each session, once a day. | CT | 3 M |
| Li Hang 2020 | rTMS+CT. rTMS stimulates the bilateral dorsolateral prefrontal cortex at 60% of MT, with a frequency of 3Hz. Each sequence consists of 30 repetitions, performed once daily, five times per week. | CT | 4 W |
| Guo Ling 2021 | rTMS+CT. rTMS stimulates the right dorsolateral prefrontal cortex at 10Hz, 80% of MT intensity, for 20 minutes each session, five times a week. | CT | 6 M |
|  | EA, using a connecting electroacupuncture device with a frequency of 1.3 to 1.7Hz, leaving the needles in for 30 minutes.  Selected acupoints: Baihui (DU20), Sishencong (EX-HN1), Shenmen (HT7), Sanyinjiao (SP6), Zusanli (ST36), Fenglong (ST40), Shangxing (DU23), Daling (PC7), Quchi (LI11), Shenmai (BL62). |  |  |
| Pan Hongshan 2018 | rTMS+Acupuncture+Moxibustion+CT. rTMS stimulates the frontal lobe at a frequency of 20 Hz, with 30 pulses as one sequence, once daily. Moxibustion, lasting 20 minutes each session, combined with acupuncture once daily, six times a week.  Selected acupoints: Shenting (DU24), Baihui (DU20), Dazhui (DU14). | CT | 12 W |
| Wu Xiaohui 2020 | rTMS targeting the frontal lobe at a frequency of 20 Hz, with 30 repetitions per sequence, once daily. Moxibustion for 20 minutes each session, combined with acupuncture once daily, six times per week.  Selected acupoints: Shenting (DU24), Baihui (DU20), Dazhui (DU14). | CT | 12 W |
| Cheng Nanfang 2021 | Acupuncture with needle retention for 30 minutes after achieving De Qi sensation, once daily, six times per week.  Selected acupoints: Neiguan (PC6), Renzhong (GV26), Sanyinjiao (SP6) | CT | 30 D |
| Meng Yingchun 2011 | ACUP+CT. Needling with retaining the needle for 30 minutes after obtaining Qi, once a day.  Selected acupoints: Danzhong (CV17), Zhongwan (CV12), Qihai (CV6), Xuehai (SP10), Zusanli (ST36). | CT | 6 W |
| Han Hui 2021 | ACUP+CT. Retain the needle for 30 minutes after needling the acupoint, once a day, five times a week.  Selected acupoints: Baihui (DU20), Sishencong (EX-HN1), Zhongwan (RN12), Waiguan (SJ5), Xiawan (RN10), Qihai (CV6), Guanyuan (RN4), Yindu (KI19), Shangqu (KI17), Tianshu (ST25), Daheng (SP15), Fenglong (ST40), Huaroumen (ST24). | CT | 8 W |
| Qiao Shiguang 2023 | ACUP+CT. Needle insertion with manipulation, once daily.  Selected acupoints: Baihui (DU20), Sanyinjiao (SP6), Neiguan (PC6), Sishencong (EX-HN1), Shenting (DU 24), Renzhong (GV26), Jingjiaji (EX-B2), Taixi (KI3), Yongquan (KI1). | CT | 20 D |
| Chen Yongjun 2011 | ACUP+CT. Needle insertion with manipulation, retain the needle for 30 minutes, once daily.  Selected acupoints: Baihui (DU20), Sanyinjiao (SP6), Neiguan (PC6), Renzhong (GV26), Shenshu (BL23). | CT | 30 D |
| Wang Huazheng 2015 | ACUP+CT. Needle insertion with manipulation, once daily, six times a week.  Selected acupoints: Baihui (DU20), Sishencong (EX-HN1), Neiguan (PC6), Renzhong (GV26), Sanyinjiao (SP6), Fengchi (GB20), Yintang (EX-HN3), Shenmen (HT7). | CT | 8 W |
| Hu Fengxia 2019 | ACUP+CT. once daily, treatment for 6 days per week.  Selected acupoints: Head Acupoints. | CT | 8 W |
| Feng Xiaorong 2019 | ACUP+CT. Retain the needle for 30 minutes after obtaining qi during acupuncture, once daily.  Selected acupoints: Jianzhen (SI9), Neiguan (PC6), Daling (PC7), Laogong (PC8). | CT | 8 W |
| Ye Baoye 2016 | ACUP+CT. Retain the needle for 30 minutes after obtaining qi during acupuncture. Once daily, 6 times a week.  Selected acupoints: Baihui (DU20), Shenting (DU24), Sishencong (EX-HN1), Touwei (ST8). | CT | 4 W |
| Zhang Shaolei 2022 | ACUP+CT. Retain the needle for 30 minutes after obtaining qi during acupuncture, once daily in the morning, for consecutive treatment over 6 days.  Selected acupoints: Baihui (DU20), Sishencong (EX-HN1), Shenmen (HT7), Neiguan (PC6), Sanyinjiao (SP6), Tongli (HT5), Dazhong (KI4), Yintang (EX-HN3), Fengchi (GB20). | CT | 12 W |
| Cui Lele 2015 | ACUP+CT. Retain the needle for 40 minutes after obtaining qi during acupuncture, with one needle manipulation during the session. Once daily, 6 times a week.  Selected acupoints: Baihui (DU20), Shenting (DU24), Fengfu (DU16), Yamen (GV15), Shuigou (GV26), Dazhui (DU14), Zhiyang (GV9), Yaoyangguan (DU3). | CT | 4 W |
| Yu Tao 2007 | ACUP. Acupuncture with retaining needles for 30 minutes, once daily, five times per week.  Selected acupoints: Renzhong (CV17), Zhongwan (CV12), Qihai (CV6), Xuehai (SP10), Zusanli (ST36), Waiguan (TE5) | CT | 12 W |
| Hu Jingjing 2009 | The ACUP method involves retaining the needle for 30 minutes, once a day, six times a week.Selected acupoints: Baihui (DU20), Sishencong (EX-HN1). | CT | 90 D |
| Tan Tao 2017 | The ACUP method involves retaining the needle for 30 minutes, once a day.  Selected acupoints: Baihui (DU20), Shenting (DU24), Shuigou (DU26), Neiguan (PC6), Daling (PC7), Laogong (PC8), Dazhui (DU14), Fengfu (DU16), Tanzhong (CV17), Hegu (LI4), Zusanli (ST36), Taichong (LR3). | CT | 30 D |
| Zhang Rui 2010 | The ACUP method involves retaining the needle for 40 minutes, performing needle manipulation once during the session, once daily.  Selected acupoints: Baihui (DU20), Shenting (DU24), Dazhong (KI4), Fengchi (GB20), Neiguan (PC6), Shenmen (HT7), Zusanli (ST36), Sanyinjiao (SP6). | CT | 8 W |
| Zheng Shenghui 2011 | EA+CT. After needling to obtain qi, connect to an electroacupuncture device. Electroacupuncture once daily, retaining the needle for 30 minutes, 5 times per week.  Selected acupoints: Baihui (DU20), Shenting (DU24), Benshen (DU22), Qianding Xue (DU21), Houding Xue (DU20). | CT | 8 W |
| Gao Qianren 2013 | EA+CT. After the acupuncture, connect the electroacupuncture apparatus. Administer electric acupuncture once daily, leave the needle in for 30 minutes, 6 times per week.  Selected acupoints: Shen Ting (DU24), Ben Shen (DU22). | CT | 8 W |
| Yao Weiling 2018 | EA+CT. After acupuncture, connect the electroacupuncture apparatus, leave the needle in for 20 minutes, once daily.  Selected acupoints: Baihui (DU20), Sishencong (EX-HN1), Shenting (DU24), Shenmen (HT7), Fengchi (GB20). | CT | 30 D |
| Xu Lihong 2006 | EA+CT. After needling for qi, connect the electroacupuncture apparatus, leave the needle in for 30 minutes, once daily.  Selected acupoints: Baihui (DU20), Shenting (DU24), Sishencong (EX-HN1). | CT | 3 M |
|  | EA. After needling for qi, connect the electroacupuncture apparatus, leave the needle in for 30 minutes, once daily.  Selected acupoints: Baihui (DU20), Shenting (DU24), Sishencong (EX-HN1). |  |  |
| Liu Lijie 2022 | RT+CT. Training duration is 30 to 60 mins per session, 4 to 6 times per week. | CT | 12 W |
| Yang Xiaoyan 2008 | EA+CT. Electroacupuncture treatment for 30 minutes, once daily, six times per week.  Selected acupoints: Baihui (DU20),Shenting (DU24), Sishencong (EX-HN1). | CT | 2 M |
|  | EA. Electroacupuncture treatment for 30 minutes, once daily, six times per week.  Selected acupoints: Baihui (DU20),Shenting (DU24), Sishencong (EX-HN1). |  |  |
| Peng Xiaohong 2009 | EA+CT. Electroacupuncture treatment for 30 minutes, once daily, five times per week.  Selected acupoints: Baihui (DU20),Shenting (DU24), Sishencong (EX-HN1), Fengchi (GB20). | CT | 6 W |
|  | EA. Electroacupuncture treatment for 30 minutes, once daily, five times per week.  Selected acupoints: Baihui (DU20),Shenting (DU24), Sishencong (EX-HN1), Fengchi (GB20). |  |  |
| Zhao Ling 2009 | EA+CT. Electroacupuncture treatment for 30 minutes, once daily, five times per week.  Selected acupoints: Sishencong (EX-HN1), Baihui (GV 20), Shenting (GV 24) and Fengchi (GB 20), | CT | 6 W |
|  | EA. Electroacupuncture treatment for 30 minutes, once daily, five times per week.  Selected acupoints: Sishencong (EX-HN1), Baihui (GV 20), Shenting (GV 24) and Fengchi (GB 20), |  |  |
| Wang Huiming 2007 | EA. Acupuncture twice a day, with 30 minutes of needling each time, for a total of six treatments per week.  Selected acupoints: Baihui (DU20), Sishencong (EX-HN1), Fengchi (GB20), Shuigou (GV26), Yintang (EX-HN3), Shangxing (GV23). | CT | 3 M |
| Yin Jianquan 2011 | EA. Electroacupuncture therapy for 30 minutes, once daily, five times a week.  Selected acupoints: Baihui (DU20),Shenting (DU24), Sishencong (EX-HN1). | CT | 12 W |
| Li Qiaowei 2015 | EA. Electroacupuncture treatment for 30 minutes, once a day, five times a week.  Selected acupoints: Shenting (DU24), Benshen (GB13) | CT | 4 W |
| Wang Minchao 2020 | MB+CT. Moxibustion every two days, for 60 minutes each session.  Selected acupoints: Baihui (GV 20), Sishencong (EX-HN 1), bilateral Xuehai (SP 10), Xuanzhong (GB 39) and Taichong (LR 4) | CT | 8 W |
| Sheng Dandan 2017 | MB+CT. Moxibustion for 60 minutes each session, once a day, six times a week.  Selected acupoints: Mingmen (DU4), Dazhui (DU14) | CT | 4 W |
| Gao Yujie 2018 | MB+CT.  Selected acupoints: Sishencong (EX-HN1), Fengchi (GB20), Benshen (GB13). | CT | 8 W |
| Luo Benhua 2015 | MB.  Selected acupoints: Shanzhong (Ren17), Zhongwan (Ren12), Qihai (Ren6), Xuehai (SP10), Zusanli (ST36), Waiguan (SJ5). | CT | 8 W |
|  | ACUP. Retaining acupuncture needle for 30 minutes, once every other day.  Selected acupoints: Shanzhong (Ren17), Zhongwan (Ren12), Qihai (Ren6), Xuehai (SP10), Zusanli (ST36), Waiguan (SJ5). |  |  |
| Fan Weiquan 2019 | ACUP+MB+CT. Five times a week.  Selected acupoints: Baihui (DU20), Zusanli (ST36), Sanyinjiao (SP6), Neiguan (PC6), Sishencong (EX-HN1), Zhenxia Panghai (GB12), Fengchi (GB20), Hegu (LI4), Taichong (LR3), Shenque (CV8). | CT | 12 W |
| Zhao Ruixia 2012 | ACUP+MB+CT.  Selected acupoints: Baihui (DU20), Zusanli (ST36), Sanyinjiao (SP6), Neiguan (PC6), Shenshu (BL23), Shenmen (HT7), Shuigou (GV26), Dazhui (DU14). | CT | 100 D |
| Ma Li 2018 | ACUP+MB+CT. Acupuncture once a day, continuous treatment for 6 days a week, moxibustion at Dazhui acupoint for 1 hour each day, once every other day.  Selected acupoints: Baihui (DU20), Dazhui (DU14) | Acupuncture and cupping therapy once a day, continuous treatment for 6 days a week. | 8 W |
| Wang Heng 2021 | ACUP+MB+CT. Place a moxa stick 2-3cm above the tip of the needle, ignite the moxa stick for moxibustion, remove the ashes after the moxa stick burns out, leave the needle in for 30 minutes after moxibustion, once a day, continuous treatment for 5 days a week.  Selected acupoints: Yongquan (KI1), Baihui (DU20), Shenting (DU24), Sishencong (EX-HN1), Zusanli (ST36), Sanyinjiao (SP6), Taixi (KI3). | ACUP+CT. After obtaining qi with acupuncture, leave the needle in for 30 minutes, once a day, 5 times a week.  Selected acupoints: Baihui (DU20), Yintang (EX-HN3), Shenting (DU24), Taixi (KI3), Sishencong (EX-HN1), GV16 (DU16), Shenshu (BL23) | 6 W |
| Wang Xuepeng 2018 | RT+CT. Train once a day, for 40-50 minutes each session. | CT | 3 M |
| Wang Huaijing 2007 | RT+CT. | CT | 12 W |
| Sun Peijun 2016 | RT+CT. Each training session lasts 1 hour or as deemed appropriate until the patient feels no fatigue, twice a day, for 5 days a week. | CT | 12 W |
| Zhai Zhiyong 2015 | RT+CT. Each treatment lasts 1 hour, 4 times a week. | CT | 6 M |
| Wu Xiaoling 2018 | CFT+CT. Train once a day, for 30 minutes each session. | CT | 2 M |
| Ji Jing 2017 | CFT+CT. Train twice a day, for 30 minutes each session. | CT | 2 M |
| Zhu Kaifeng 2023 | CFT+CT. | CT | 3 M |
| Qu Yancao 2019 | CFT+CT. Train once a day, for 30 minutes each session. | CT | 8 W |
| Zhao Pengxiang 2015 | HBO+CT. Maintain hyperbaric oxygen therapy at 0.2 MPa, pressurize for around 20 minutes, depressurize for around 25 minutes, then maintain pressure and inhale oxygen for 1 hour. Rest for 5 minutes after every 0.5 hour of inhaling oxygen. Treatment once a day. | CT | 3 M |
| Liu Li 2015 | HBO+CT, the pressure in the hyperbaric oxygen chamber is 0.2~0.25 MPa, breathing pure oxygen for 60 minutes each time, once daily. | CT | 10 D*(4-5) |
| Chen Shidong 2011 | HBO+CT, the pressure in the hyperbaric oxygen chamber is 0.2~0.25 MPa, oxygen inhalation time is 60 minutes, once per day. | CT | 5*10 D |
| Liu Zhiyi 2016 | HBO+CT, the hyperbaric oxygen therapy is adjusted to 0.2~0.25 MPa, 60 minutes per session, once daily. | CT | 5*10 D |
| Lei Xiaodan 2016 | HBO+CT, hyperbaric oxygen therapy, 1 hour per day. | CT | 3 W |
| Sun Wei 2015 | HBO+CT. Hyperbaric oxygen therapy chamber pressure 0.2 MPa, lasting 20 min, 23 min decompression, 40 min oxygen inhalation, 5 min interval rest. Hyperbaric oxygen therapy once daily. | CT | 24 D |
| Wang Yongsheng 2013 | HBO+CT. Hyperbaric oxygen therapy chamber pressure 0.2 MPa, pressurization time 15-20 minutes, decompression time 20-25 minutes, stable oxygen inhalation lasting 60 minutes, oxygen inhalation every 30 minutes, rest for 5 minutes, once a day. | CT | 60 D |
| Wang Shuiping 2009 | HBO+CT. The hyperbaric oxygen therapy chamber pressure is 0.2 MPa, for 60 minutes every day, once a day. | CT | 12 W |
| Tang Longchong 2013 | HBO+CT. The hyperbaric oxygen therapy chamber pressure is 0.2 MPa, pressurization time is 15-20 minutes, decompression time is 20-25 minutes, stable oxygen inhalation duration is 60 minutes, each oxygen inhalation session lasts 30 minutes, rest for 5 minutes, once daily. | CT | NA |
| Li Wen 2013 | HBO+CT. The hyperbaric oxygen therapy chamber pressure ranges from 0.2 to 0.25 MPa, with an oxygen inhalation time of 60 minutes per session, once daily. | CT | 5*10 D |
| Feng Yufeng 2020 | HBO+CT. The hyperbaric oxygen therapy pressure is 0.2 MPa, with continuous pressurization for 20 minutes, followed by decompression for 25 minutes. Subsequently, under the condition of maintaining the hyperbaric oxygen therapy pressure, oxygen inhalation is carried out for 1 hour. During the oxygen inhalation process, rest for 5 minutes every 30 minutes. This procedure is done once daily. | CT | 1 M |
| Hu Qian 2023 | HBO+CT. The hyperbaric oxygen therapy chamber pressure is set at 0.15 to 0.75 MPa, with oxygen inhalation lasting 80 minutes per session, once daily. | CT | 1 M |
| Wang Yongsheng 2014 | HBO+CT. The hyperbaric oxygen therapy chamber pressure is 0.2 MPa, with pressurization lasting 15-20 minutes, decompression lasting 20-25 minutes, and stabilized oxygen inhalation lasting 60 minutes. Oxygen is inhaled every 30 minutes with a 5-minute break in between. This procedure is performed once daily. | CT | 8 W |
| Yang Li 2018 | HBO+CT. The hyperbaric oxygen therapy chamber pressure is set at 0.2 MPa, with pressurization lasting 15-20 minutes, decompression lasting 20-25 minutes, and stable oxygen inhalation for 60 minutes. Oxygen is inhaled every 30 minutes with a 5-minute break in between. This treatment is conducted once daily. | CT | 4 W |
| Li Zicheng 2020 | HBO+CT. The pressure in the hyperbaric oxygen therapy chamber is set between 0.15 and 0.75 MPa, with 80 minutes of oxygen inhalation once a day. | CT | 14 D |
| Wang Songlin 2011 | HBO+CT. The hyperbaric oxygen therapy chamber pressure is set at 0.2 to 0.25 MPa, with 60 minutes of oxygen inhalation once a day. | CT | 5*10 D |
| Xia Xiyuan 2012 | HBO+CT. The hyperbaric oxygen therapy chamber pressure is set at 0.2 to 0.25 MPa, with 30 minutes of oxygen inhalation twice a day. | CT | 4 W |
| Bao Zhiying 2007 | HBO+CT. The hyperbaric oxygen therapy chamber pressure is set to reach 0.2 MPa in 20 minutes, followed by inhaling pure oxygen for 60 minutes (with an intermediate rest of 10 minutes), and then decompression for 25-30 minutes. | CT | 4 D*10 |
| Wu Yong 2010 | HBO+CT, hyperbaric oxygen therapy therapy for 1 hour per day. | CT | 3 W |
|  | HBO, hyperbaric oxygen therapy therapy for 1 hour per day. |  |  |
| Song Yuhua 2012 | HBO+CT, hyperbaric oxygen therapy therapy for 1 hour per day. | CT | 3 W |
|  | HBO, hyperbaric oxygen therapy therapy for 1 hour per day. |  |  |
| Bu Guiwen 2012 | HBO+CT, hyperbaric oxygen therapy therapy for 1 hour per day. | CT | 3 W |
|  | HBO, hyperbaric oxygen therapy therapy for 1 hour per day. |  |  |
| Liu Yuqian 2020 | EMGBFB+CT. Electromyography biofeedback each treatment lasts 1 hour, with 30 minutes for each upper and lower limb, once a day, 6 times a week. | CT | 4 W |
| Ran Qian 2018 | EMGBFB+CT. Electromyography biofeedback treatment lasts 1 hour each session, with 30 minutes for each upper and lower limb, once a day, 5 times a week. | CT | 4 M |
| Du Huiqing 2015 | EMGBFB+CT. Electromyography biofeedback treatment lasts 1 hour each session, with 30 minutes for each upper and lower limb, once a day, 5 times a week. | CT | 6 M |
| Liu Yalin 2022 | EMGBFB+CT. Electromyography biofeedback treatment lasts 30 minutes each session, once a day. | CT | 1 M |
| Cai Rui 2018 | EMGBFB+CT. Electromyography biofeedback treatment lasts for 30 minutes each session, once a day, five times a week. | EMGBFB. Electromyography biofeedback treatment lasts for 30 minutes each session, once a day, five times a week. | 3 M |
| Chen Songsheng 2013 | FNS+CT. The main electrodes are placed on both sides of the mastoid process. Each treatment session lasts for 1 hour, twice a day. Treatment parameters set at: frequency 136%, intensity 90%. | CT | 2*10 D |
| Wu Bingjie 2005 | FNS+CT. The main electrodes are placed on both sides of the mastoid process. The frequency is set at 50-100Hz, with each treatment session lasting 30-45 minutes, once a day. | CT | 15 D |
| Dai Jianwu 2008 | FNS+CT. The main electrodes are placed on both sides of the mastoid process. The frequency is set at 130%-135% and the intensity is set at 80%-100%. Treatments are administered twice a day, with each session lasting 30 minutes. | CT | 4 W |
| Li Ji 2023 | ACUP+RT+CT. After obtaining the qi with acupuncture, leave the needles in for 30 minutes, once a day. After receiving treatment continuously for 6 times per week, take a rest day.  Selected acupoints: Guan Yuan (CV4), Shen Shu (BL23), Xue Hai (SP10), Tai Xi (KI3), He Gu (LI4). | RT+CT. Rehabilitation training same as the experimental group. | 8 W |
| Wu Fuyou 2022 | ACUP+RT+CT. Needle insertion with retention for 30 minutes each time, twice a day, five times a week. Rehabilitation training twice a day, five days a week.  Selected acupoints: Ren Zhong (GV26), Si Shen Cong (EX-HN1), ShenTing (DU24), Shen Men (HT7), Nei Guan (PC6), Xue Hai (SP10), Tai Chong (LR3), Ran Gu (KI2). | RT+CT. Rehabilitation training same as the experimental group. | 4 W |
| Li Weimin 2023 | ACUP+RT+CT. Needle insertion for 30 minutes each session, once a day, six times a week; Rehabilitation training once a day, six times a week.Selected acupoints: Baihui (DU20), Si Shen Cong (EX-HN1), Shenting (DU24), Benshen (GB13), Fengchi (GB20), Zusanli (ST36), Xuehai (SP10), Jiaosun (GV2), Lüegu (GB34), Xuánlí (GB31), Qubin (GB7). | RT+CT. Rehabilitation training same as the experimental group. | 12 W |
| Li Weimin 2017 | ACUP+RT+CT. Needle insertion with retention for 30 minutes, once a day; Rehabilitation training 1-2 times a day.  Selected acupoints: Jiaosun (GV2), Lüegu (GB34), Xuánlí (GB31), Qubin (GB7) | RT+CT. Rehabilitation training same as the experimental group. | 12 W |
| Wang Haiyan 2009 | ACUP+RT+CT。Selected acupoints: Baihui (DU20), Sishencong (EX-HN1), Neiguan (PC6), Shenshu (BL23), Taixi (KI3), Zusanli (ST36), Ganshu (BL18), Fenglong (ST40) | CT | 8 W |
| Shi Guirong 2011 | AA+MB. Ear acupressure pressing three times a day, alternating between both ears, once every three days; Moxibustion on each acupoint for 10 minutes.  Selected acupoints:  Ear acupoints: Nao, Xin, Pi.  Moxibustion: Bilateral Pishu (BL20), Shenshu (BL23). | CT | 12 W |
|  | AA. Auricular acupuncture pressing three times a day, alternating between both ears, once every three days.  Selected acupoints: Shen, Pi, Xin, Nao for ear acupressure. |  |  |
| Chen Qi 2009 | AA. Rubbing and pressing 5 times a day, 5 minutes each time. Alternating between both ears, changing daily.  Selected acupoints: Shenmen (MA-TF1), Nao, Shen, Zhen. | CT | 12 W |
| Kuang Weichuan 2012 | AA+MB. Press 3 times a day until a slight swelling is felt, alternating between both ears, changing every 2 days. Moxibustion on each acupoint for 10 minutes, once a day, 6 times a week.  Selected acupoints: Pizhixia (MA-Atl), Nie, E. | CT | 12 W |
|  | AA. Press 3 times a day until a slight swelling is felt, alternating between both ears, changing every 2 days.  Selected acupoints: Pizhixia (MA-Atl), Nie, E. |  |  |
| Wang Xiaoyan 2019 | ACUP. Insert the needle to obtain qi, leaving it in for 20 minutes, once a day.Selected acupoints: Baihui (DU20), Sishencong (EX-HN1), Taixi (KI3), Xuanchong (GB42), Zusanli (ST36), Qihai (CV6), Xuehai (SP10), Sanyinjiao (SP6) | CT | 12 W |
| Wang Linjuan 2014 | ACUP. Retain the needle for 30 minutes after obtaining qi, once a day.  Selected acupoints: Baihui (DU20), Sishencong (EX-HN1), Shenting (DU24), Waiguan (SJ5), Fenglong (ST40), Qihai (CV6), Xuehai (SP10), Zusanli (ST36) | CT | 8 W |
| Liu Suhua 2016 | AE+CT. Exercise for 1 hour in the morning and evening, persist in exercising for 30 days. | CT | 30 D |
| Li Rong 2017 | FNS+CT. | CT | 8 W |

m months, w weeks, d days, AA auricular acupuncture, ACUP acupuncture, AE Aerobic exercise, CFT Cognitive function training, CT Conventional treatment, EMGBFB electromyographic biofeedback, EA electroacupunctur, FNS Fastigial nucleus stimulation, HBO hyperbaric oxygen therapy, MB moxibustion, RT Rehabilitation training, rTMS Repetitive Transcranial Magnetic Stimulation.

# Appendix 5: Risk of Bias

Figure 5.1 The risk of bias assessment for the individual included studies

# Appendix 6: Adverse reactions

Table 5.1 Included studies adverse events details

| Included studies | traetment | adverse reactions |
| --- | --- | --- |
| Yang Mingjian 2023 | rTMS_CT | 3 cases of transient headache |
|  | CT | NA |
| Guo Ling 2021 | rTMS_CT | NA |
|  | EA_CT | NA |
|  | CT | 2 cases of burning sensation in the genital area |
| Hu Fengxia 2019 | ACUP_CT | 1 case of nausea, 1 case of rash, 1 case of headache |
|  | CT | 2 case of nausea, 1 case of rash, 2 case of headache |
| Feng Xiaorong 2019 | ACUP_CT | NA |
|  | CT | 1 case of nausea |
| Wang Minchao 2020 | MB_CT | 2 cases of dry mouth |
|  | CT | 2 cases of insomnia, 1 case of loss of appetite |
| Fan Weiquan 2019 | ACUP_MB_CT | 1 case of nausea, 1 case of skin redness, 1 case of dizziness |
|  | CT | 1 case of nausea, 1 case of skin redness, 1 case of dizziness |
| Sun Wei 2015 | HBO_CT | 1 case of nausea, 1 case of abdominal pain |
|  | CT | 1 case of nausea, 2 case of abdominal pain，6 cases of mental abnormalities |
| Hu Qian 2023 | HBO_CT | 2 cases of earache, 1 case of diarrhea |
|  | CT | 1 case of abdominal pain |
| Yang Li 2018 | HBO_CT | 2 cases of nausea and vomiting, 2 cases of abdominal pain, 3 cases of dizziness, 1 case of high pressure oxygen injury |
|  | CT | 3 cases of nausea and vomiting, 1 case of abdominal pain, 2 cases of dizziness, 1 case of blurred vision |
| Xia Xiyuan 2012 | HBO_CT | 1 case of mild palpitations, 1 case of insomnia |
|  | CT | 1 case of dizziness, 1 case of insomnia |
| Wu Yong 2010 | HBO_CT | 1 case of elevated blood pressure |
|  | HBO | 2 cases of elevated blood pressure |
|  | CT | NA |
| Ran Qian 2018 | EMGBFB_CT | 1 case of abdominal discomfort, 2 cases of nausea, 2 cases of headache |
|  | CT | 2 cases of abdominal discomfort, 1 case of headache |
| Liu Yalin 2022 | EMGBFB_CT | 3 cases of nausea and vomiting, 4 cases of dizziness, 1 case of blurred vision |
|  | CT | 2 cases of nausea and vomiting, 3 cases of dizziness, 1 case of blurred vision |
| Dai Jianwu 2008 | FNS_CT | NA |
|  | CT | 1 case of mild diarrhea, 2 cases of insomnia |

# Appendix 7: Evaluation of heterogeneity and inconsistency

## 7.1 Heterogeneity

| Outcomes | Number of studies | Heterogeneity | | | | | Heterogeneity  assessment |
| --- | --- | --- | --- | --- | --- | --- | --- |
|  |  | τ^2^ | Q | df | *P* | I^2^ |  |
| MMSE | 89 | 0.9763 | 310.89 | 80 | < 0.0001 | 74.3% | moderate to high |
| ADL | 27 | 2.0683 | 35.42 | 16 | 0.0035 | 54.8% | moderate to high |

We utilize the tau square (τ2) test and p-value to qualitatively analyze the statistical heterogeneity across studies. A larger τ2 and a smaller p-value indicate a higher likelihood of heterogeneity; conversely, smaller values suggest lower heterogeneity. Additionally, I2 serves as a parameter for quantitatively assessing heterogeneity among study results, ranging from 0 to 100%. An I2 below 25% suggests low heterogeneity, while 25%-50% indicates moderate heterogeneity, and I2 exceeding 75% signifies high heterogeneity. In summary, an I2 greater than 50% implies substantial heterogeneity.

Table 7.1 Global heterogeneity

## 7.2 Inconsistency (global inconsistency and SIDE splitting results)

Table 7.2.1 Summary of the global inconsistency and SIDE splitting results

| Outcomes | SIDE splitting Number of inconsistent comparisons out of total | Percentage of inconsistent comparisons out of total | The Design-by-Treatment test | | | |
| --- | --- | --- | --- | --- | --- | --- |
|  |  |  | Q | df | τ^2^ | *p*-value |
| MMSE | 0 | 0% | 15.54 | 9 | 0.7332 | 0.0770 |
| ADL | 0 | 0% | 1.74 | 1 | 1.4219 | 0.1875 |

Table 7.2.2 Details of SIDE splitting results （MMSE）

| **comparison** | **k** | **prop** | **NMA** | | **Direct** | | **Indir** | | **Diff** | | **z** | **p** |
| --- | --- | --- | --- | --- | --- | --- | --- | --- | --- | --- | --- | --- |
|  |  |  | **TE** | **seTE** | **TE** | **seTE** | **TE** | **seTE** | **TE** | **seTE** |  |  |
| AA vs AA_MB | 2 | 0.900741 | -2.37213 | 0.81394 | -2.18903 | 0.857615 | -4.03367 | 2.5835 | 1.844637 | 2.722127 | 0.677645 | 0.497997 |
| AA vs ACUP | 0 | 0 | -0.64471 | 0.834467 | NA | NA | -0.64471 | 0.834467 | NA | NA | NA | NA |
| AA vs ACUP_CT | 0 | 0 | -2.33587 | 0.784163 | NA | NA | -2.33587 | 0.784163 | NA | NA | NA | NA |
| AA vs ACUP_MB_CT | 0 | 0 | -4.28535 | 0.947611 | NA | NA | -4.28535 | 0.947611 | NA | NA | NA | NA |
| AA vs ACUP_RT_CT | 0 | 0 | -3.37792 | 1.000754 | NA | NA | -3.37792 | 1.000754 | NA | NA | NA | NA |
| AA vs AE_CT | 0 | 0 | -3.44238 | 1.403464 | NA | NA | -3.44238 | 1.403464 | NA | NA | NA | NA |
| AA vs CFT_CT | 0 | 0 | -1.38961 | 0.965299 | NA | NA | -1.38961 | 0.965299 | NA | NA | NA | NA |
| AA vs CT | 3 | 0.999666 | 0.807621 | 0.690647 | 0.837493 | 0.690762 | NA | NA | NA | NA | NA | NA |
| AA vs EA | 0 | 0 | -0.69978 | 0.8388 | NA | NA | -0.69978 | 0.8388 | NA | NA | NA | NA |
| AA vs EA_CT | 0 | 0 | -0.95861 | 0.826193 | NA | NA | -0.95861 | 0.826193 | NA | NA | NA | NA |
| AA vs EMGBFB | 0 | 0 | 1.36499 | 1.644014 | NA | NA | 1.36499 | 1.644014 | NA | NA | NA | NA |
| AA vs EMGBFB_CT | 0 | 0 | -1.96501 | 0.887203 | NA | NA | -1.96501 | 0.887203 | NA | NA | NA | NA |
| AA vs FNS_CT | 0 | 0 | -3.70536 | 1.198229 | NA | NA | -3.70536 | 1.198229 | NA | NA | NA | NA |
| AA vs HBO | 0 | 0 | 0.794102 | 0.931074 | NA | NA | 0.794102 | 0.931074 | NA | NA | NA | NA |
| AA vs HBO_CT | 0 | 0 | -2.42669 | 0.741292 | NA | NA | -2.42669 | 0.741292 | NA | NA | NA | NA |
| AA vs MB | 0 | 0 | -0.4557 | 1.265984 | NA | NA | -0.4557 | 1.265984 | NA | NA | NA | NA |
| AA vs MB_CT | 0 | 0 | -2.51426 | 1.038028 | NA | NA | -2.51426 | 1.038028 | NA | NA | NA | NA |
| AA vs RT_CT | 0 | 0 | -0.97913 | 0.85836 | NA | NA | -0.97913 | 0.85836 | NA | NA | NA | NA |
| AA vs rTMS_ACUP_MB_CT | 0 | 0 | -2.15461 | 1.09329 | NA | NA | -2.15461 | 1.09329 | NA | NA | NA | NA |
| AA vs rTMS_CT | 0 | 0 | -3.16909 | 0.829244 | NA | NA | -3.16909 | 0.829244 | NA | NA | NA | NA |
| AA_MB vs ACUP | 0 | 0 | 1.727421 | 0.932026 | NA | NA | 1.727421 | 0.932026 | NA | NA | NA | NA |
| AA_MB vs ACUP_CT | 0 | 0 | 0.036256 | 0.88727 | NA | NA | 0.036256 | 0.88727 | NA | NA | NA | NA |
| AA_MB vs ACUP_MB_CT | 0 | 0 | -1.91322 | 1.034555 | NA | NA | -1.91322 | 1.034555 | NA | NA | NA | NA |
| AA_MB vs ACUP_RT_CT | 0 | 0 | -1.0058 | 1.083441 | NA | NA | -1.0058 | 1.083441 | NA | NA | NA | NA |
| AA_MB vs AE_CT | 0 | 0 | -1.07025 | 1.463574 | NA | NA | -1.07025 | 1.463574 | NA | NA | NA | NA |
| AA_MB vs CFT_CT | 0 | 0 | 0.98252 | 1.050781 | NA | NA | 0.98252 | 1.050781 | NA | NA | NA | NA |
| AA_MB vs CT | 2 | 0.908493 | 3.179748 | 0.805811 | 3.367649 | 0.845419 | 1.314237 | 2.663826 | 2.053412 | 2.794764 | 0.734735 | 0.462501 |
| AA_MB vs EA | 0 | 0 | 1.672342 | 0.935908 | NA | NA | 1.672342 | 0.935908 | NA | NA | NA | NA |
| AA_MB vs EA_CT | 0 | 0 | 1.41352 | 0.924625 | NA | NA | 1.41352 | 0.924625 | NA | NA | NA | NA |
| AA_MB vs EMGBFB | 0 | 0 | 3.737116 | 1.695618 | NA | NA | 3.737116 | 1.695618 | NA | NA | NA | NA |
| AA_MB vs EMGBFB_CT | 0 | 0 | 0.407116 | 0.979524 | NA | NA | 0.407116 | 0.979524 | NA | NA | NA | NA |
| AA_MB vs FNS_CT | 0 | 0 | -1.33323 | 1.268105 | NA | NA | -1.33323 | 1.268105 | NA | NA | NA | NA |
| AA_MB vs HBO | 0 | 0 | 3.166229 | 1.019429 | NA | NA | 3.166229 | 1.019429 | NA | NA | NA | NA |
| AA_MB vs HBO_CT | 0 | 0 | -0.05457 | 0.849618 | NA | NA | -0.05457 | 0.849618 | NA | NA | NA | NA |
| AA_MB vs MB | 0 | 0 | 1.916429 | 1.332311 | NA | NA | 1.916429 | 1.332311 | NA | NA | NA | NA |
| AA_MB vs MB_CT | 0 | 0 | -0.14214 | 1.117962 | NA | NA | -0.14214 | 1.117962 | NA | NA | NA | NA |
| AA_MB vs RT_CT | 0 | 0 | 1.392995 | 0.953478 | NA | NA | 1.392995 | 0.953478 | NA | NA | NA | NA |
| AA_MB vs rTMS_ACUP_MB_CT | 0 | 0 | 0.217515 | 1.169453 | NA | NA | 0.217515 | 1.169453 | NA | NA | NA | NA |
| AA_MB vs rTMS_CT | 0 | 0 | -0.79696 | 0.927353 | NA | NA | -0.79696 | 0.927353 | NA | NA | NA | NA |
| ACUP vs ACUP_CT | 0 | 0 | -1.69117 | 0.597713 | NA | NA | -1.69117 | 0.597713 | NA | NA | NA | NA |
| ACUP vs ACUP_MB_CT | 0 | 0 | -3.64064 | 0.800197 | NA | NA | -3.64064 | 0.800197 | NA | NA | NA | NA |
| ACUP vs ACUP_RT_CT | 0 | 0 | -2.73322 | 0.862471 | NA | NA | -2.73322 | 0.862471 | NA | NA | NA | NA |
| ACUP vs AE_CT | 0 | 0 | -2.79767 | 1.308457 | NA | NA | -2.79767 | 1.308457 | NA | NA | NA | NA |
| ACUP vs CFT_CT | 0 | 0 | -0.7449 | 0.821067 | NA | NA | -0.7449 | 0.821067 | NA | NA | NA | NA |
| ACUP vs CT | 7 | 1 | 1.452327 | 0.46834 | 1.452327 | 0.46834 | NA | NA | NA | NA | NA | NA |
| ACUP vs EA | 0 | 0 | -0.05508 | 0.667783 | NA | NA | -0.05508 | 0.667783 | NA | NA | NA | NA |
| ACUP vs EA_CT | 0 | 0 | -0.3139 | 0.651877 | NA | NA | -0.3139 | 0.651877 | NA | NA | NA | NA |
| ACUP vs EMGBFB | 0 | 0 | 2.009696 | 1.563691 | NA | NA | 2.009696 | 1.563691 | NA | NA | NA | NA |
| ACUP vs EMGBFB_CT | 0 | 0 | -1.3203 | 0.727653 | NA | NA | -1.3203 | 0.727653 | NA | NA | NA | NA |
| ACUP vs FNS_CT | 0 | 0 | -3.06066 | 1.085404 | NA | NA | -3.06066 | 1.085404 | NA | NA | NA | NA |
| ACUP vs HBO | 0 | 0 | 1.438808 | 0.780543 | NA | NA | 1.438808 | 0.780543 | NA | NA | NA | NA |
| ACUP vs HBO_CT | 0 | 0 | -1.78199 | 0.540243 | NA | NA | -1.78199 | 0.540243 | NA | NA | NA | NA |
| ACUP vs MB | 1 | 0.780852 | 0.189009 | 1.067361 | -0.29 | 1.207888 | 1.895781 | 2.280041 | -2.18578 | 2.580229 | -0.84713 | 0.396924 |
| ACUP vs MB_CT | 0 | 0 | -1.86956 | 0.905456 | NA | NA | -1.86956 | 0.905456 | NA | NA | NA | NA |
| ACUP vs RT_CT | 0 | 0 | -0.33443 | 0.692193 | NA | NA | -0.33443 | 0.692193 | NA | NA | NA | NA |
| ACUP vs rTMS_ACUP_MB_CT | 0 | 0 | -1.50991 | 0.968313 | NA | NA | -1.50991 | 0.968313 | NA | NA | NA | NA |
| ACUP vs rTMS_CT | 0 | 0 | -2.52438 | 0.65574 | NA | NA | -2.52438 | 0.65574 | NA | NA | NA | NA |
| ACUP_CT vs ACUP_MB_CT | 2 | 0.594912 | -1.94948 | 0.637058 | -2.68085 | 0.825948 | -0.87539 | 1.000932 | -1.80546 | 1.297711 | -1.39126 | 0.164146 |
| ACUP_CT vs ACUP_RT_CT | 0 | 0 | -1.04205 | 0.8139 | NA | NA | -1.04205 | 0.8139 | NA | NA | NA | NA |
| ACUP_CT vs AE_CT | 0 | 0 | -1.10651 | 1.276963 | NA | NA | -1.10651 | 1.276963 | NA | NA | NA | NA |
| ACUP_CT vs CFT_CT | 0 | 0 | 0.946264 | 0.769888 | NA | NA | 0.946264 | 0.769888 | NA | NA | NA | NA |
| ACUP_CT vs CT | 11 | 0.910004 | 3.143492 | 0.371374 | 3.305976 | 0.389304 | 1.500518 | 1.23794 | 1.805458 | 1.297711 | 1.391263 | 0.164146 |
| ACUP_CT vs EA | 0 | 0 | 1.636087 | 0.603747 | NA | NA | 1.636087 | 0.603747 | NA | NA | NA | NA |
| ACUP_CT vs EA_CT | 0 | 0 | 1.377264 | 0.586105 | NA | NA | 1.377264 | 0.586105 | NA | NA | NA | NA |
| ACUP_CT vs EMGBFB | 0 | 0 | 3.700861 | 1.537435 | NA | NA | 3.700861 | 1.537435 | NA | NA | NA | NA |
| ACUP_CT vs EMGBFB_CT | 0 | 0 | 0.370861 | 0.669369 | NA | NA | 0.370861 | 0.669369 | NA | NA | NA | NA |
| ACUP_CT vs FNS_CT | 0 | 0 | -1.36949 | 1.047224 | NA | NA | -1.36949 | 1.047224 | NA | NA | NA | NA |
| ACUP_CT vs HBO | 0 | 0 | 3.129973 | 0.726514 | NA | NA | 3.129973 | 0.726514 | NA | NA | NA | NA |
| ACUP_CT vs HBO_CT | 0 | 0 | -0.09082 | 0.458736 | NA | NA | -0.09082 | 0.458736 | NA | NA | NA | NA |
| ACUP_CT vs MB | 0 | 0 | 1.880174 | 1.124117 | NA | NA | 1.880174 | 1.124117 | NA | NA | NA | NA |
| ACUP_CT vs MB_CT | 0 | 0 | -0.17839 | 0.859318 | NA | NA | -0.17839 | 0.859318 | NA | NA | NA | NA |
| ACUP_CT vs RT_CT | 0 | 0 | 1.356739 | 0.630641 | NA | NA | 1.356739 | 0.630641 | NA | NA | NA | NA |
| ACUP_CT vs rTMS_ACUP_MB_CT | 0 | 0 | 0.181259 | 0.925315 | NA | NA | 0.181259 | 0.925315 | NA | NA | NA | NA |
| ACUP_CT vs rTMS_CT | 0 | 0 | -0.83322 | 0.590399 | NA | NA | -0.83322 | 0.590399 | NA | NA | NA | NA |
| ACUP_MB_CT vs ACUP_RT_CT | 0 | 0 | 0.907425 | 0.972362 | NA | NA | 0.907425 | 0.972362 | NA | NA | NA | NA |
| ACUP_MB_CT vs AE_CT | 0 | 0 | 0.842969 | 1.383362 | NA | NA | 0.842969 | 1.383362 | NA | NA | NA | NA |
| ACUP_MB_CT vs CFT_CT | 0 | 0 | 2.895741 | 0.935833 | NA | NA | 2.895741 | 0.935833 | NA | NA | NA | NA |
| ACUP_MB_CT vs CT | 2 | 0.495084 | 5.092969 | 0.648824 | 4.181364 | 0.92212 | 5.986822 | 0.913098 | -1.80546 | 1.297711 | -1.39126 | 0.164146 |
| ACUP_MB_CT vs EA | 0 | 0 | 3.585563 | 0.804715 | NA | NA | 3.585563 | 0.804715 | NA | NA | NA | NA |
| ACUP_MB_CT vs EA_CT | 0 | 0 | 3.326741 | 0.791564 | NA | NA | 3.326741 | 0.791564 | NA | NA | NA | NA |
| ACUP_MB_CT vs EMGBFB | 0 | 0 | 5.650337 | 1.626887 | NA | NA | 5.650337 | 1.626887 | NA | NA | NA | NA |
| ACUP_MB_CT vs EMGBFB_CT | 0 | 0 | 2.320338 | 0.855049 | NA | NA | 2.320338 | 0.855049 | NA | NA | NA | NA |
| ACUP_MB_CT vs FNS_CT | 0 | 0 | 0.579986 | 1.17462 | NA | NA | 0.579986 | 1.17462 | NA | NA | NA | NA |
| ACUP_MB_CT vs HBO | 0 | 0 | 5.07945 | 0.900487 | NA | NA | 5.07945 | 0.900487 | NA | NA | NA | NA |
| ACUP_MB_CT vs HBO_CT | 0 | 0 | 1.858653 | 0.702491 | NA | NA | 1.858653 | 0.702491 | NA | NA | NA | NA |
| ACUP_MB_CT vs MB | 0 | 0 | 3.829651 | 1.243662 | NA | NA | 3.829651 | 1.243662 | NA | NA | NA | NA |
| ACUP_MB_CT vs MB_CT | 0 | 0 | 1.771083 | 1.010684 | NA | NA | 1.771083 | 1.010684 | NA | NA | NA | NA |
| ACUP_MB_CT vs RT_CT | 0 | 0 | 3.306216 | 0.825083 | NA | NA | 3.306216 | 0.825083 | NA | NA | NA | NA |
| ACUP_MB_CT vs rTMS_ACUP_MB_CT | 0 | 0 | 2.130736 | 1.067362 | NA | NA | 2.130736 | 1.067362 | NA | NA | NA | NA |
| ACUP_MB_CT vs rTMS_CT | 0 | 0 | 1.11626 | 0.794749 | NA | NA | 1.11626 | 0.794749 | NA | NA | NA | NA |
| ACUP_RT_CT vs AE_CT | 0 | 0 | -0.06446 | 1.420293 | NA | NA | -0.06446 | 1.420293 | NA | NA | NA | NA |
| ACUP_RT_CT vs CFT_CT | 0 | 0 | 1.988316 | 0.989608 | NA | NA | 1.988316 | 0.989608 | NA | NA | NA | NA |
| ACUP_RT_CT vs CT | 1 | 0.238748 | 4.185544 | 0.724234 | 2.41 | 1.482207 | 4.742399 | 0.83007 | -2.3324 | 1.69881 | -1.37296 | 0.169764 |
| ACUP_RT_CT vs EA | 0 | 0 | 2.678138 | 0.866665 | NA | NA | 2.678138 | 0.866665 | NA | NA | NA | NA |
| ACUP_RT_CT vs EA_CT | 0 | 0 | 2.419316 | 0.854468 | NA | NA | 2.419316 | 0.854468 | NA | NA | NA | NA |
| ACUP_RT_CT vs EMGBFB | 0 | 0 | 4.742912 | 1.658404 | NA | NA | 4.742912 | 1.658404 | NA | NA | NA | NA |
| ACUP_RT_CT vs EMGBFB_CT | 0 | 0 | 1.412913 | 0.913593 | NA | NA | 1.412913 | 0.913593 | NA | NA | NA | NA |
| ACUP_RT_CT vs FNS_CT | 0 | 0 | -0.32744 | 1.217897 | NA | NA | -0.32744 | 1.217897 | NA | NA | NA | NA |
| ACUP_RT_CT vs HBO | 0 | 0 | 4.172025 | 0.956253 | NA | NA | 4.172025 | 0.956253 | NA | NA | NA | NA |
| ACUP_RT_CT vs HBO_CT | 0 | 0 | 0.951228 | 0.77268 | NA | NA | 0.951228 | 0.77268 | NA | NA | NA | NA |
| ACUP_RT_CT vs MB | 0 | 0 | 2.922226 | 1.284615 | NA | NA | 2.922226 | 1.284615 | NA | NA | NA | NA |
| ACUP_RT_CT vs MB_CT | 0 | 0 | 0.863658 | 1.060671 | NA | NA | 0.863658 | 1.060671 | NA | NA | NA | NA |
| ACUP_RT_CT vs RT_CT | 4 | 0.861275 | 2.398791 | 0.587209 | 2.722353 | 0.632735 | 0.389953 | 1.576579 | 2.3324 | 1.69881 | 1.372961 | 0.169764 |
| ACUP_RT_CT vs rTMS_ACUP_MB_CT | 0 | 0 | 1.223311 | 1.114811 | NA | NA | 1.223311 | 1.114811 | NA | NA | NA | NA |
| ACUP_RT_CT vs rTMS_CT | 0 | 0 | 0.208835 | 0.857419 | NA | NA | 0.208835 | 0.857419 | NA | NA | NA | NA |
| AE_CT vs CFT_CT | 0 | 0 | 2.052772 | 1.395538 | NA | NA | 2.052772 | 1.395538 | NA | NA | NA | NA |
| AE_CT vs CT | 1 | 1 | 4.25 | 1.221768 | 4.25 | 1.221768 | NA | NA | NA | NA | NA | NA |
| AE_CT vs EA | 0 | 0 | 2.742595 | 1.311225 | NA | NA | 2.742595 | 1.311225 | NA | NA | NA | NA |
| AE_CT vs EA_CT | 0 | 0 | 2.483772 | 1.303195 | NA | NA | 2.483772 | 1.303195 | NA | NA | NA | NA |
| AE_CT vs EMGBFB | 0 | 0 | 4.807369 | 1.928343 | NA | NA | 4.807369 | 1.928343 | NA | NA | NA | NA |
| AE_CT vs EMGBFB_CT | 0 | 0 | 1.477369 | 1.342704 | NA | NA | 1.477369 | 1.342704 | NA | NA | NA | NA |
| AE_CT vs FNS_CT | 0 | 0 | -0.26298 | 1.565719 | NA | NA | -0.26298 | 1.565719 | NA | NA | NA | NA |
| AE_CT vs HBO | 0 | 0 | 4.236481 | 1.372087 | NA | NA | 4.236481 | 1.372087 | NA | NA | NA | NA |
| AE_CT vs HBO_CT | 0 | 0 | 1.015684 | 1.251094 | NA | NA | 1.015684 | 1.251094 | NA | NA | NA | NA |
| AE_CT vs MB | 0 | 0 | 2.986682 | 1.618159 | NA | NA | 2.986682 | 1.618159 | NA | NA | NA | NA |
| AE_CT vs MB_CT | 0 | 0 | 0.928115 | 1.446799 | NA | NA | 0.928115 | 1.446799 | NA | NA | NA | NA |
| AE_CT vs RT_CT | 0 | 0 | 2.463247 | 1.323823 | NA | NA | 2.463247 | 1.323823 | NA | NA | NA | NA |
| AE_CT vs rTMS_ACUP_MB_CT | 0 | 0 | 1.287767 | 1.486945 | NA | NA | 1.287767 | 1.486945 | NA | NA | NA | NA |
| AE_CT vs rTMS_CT | 0 | 0 | 0.273291 | 1.305132 | NA | NA | 0.273291 | 1.305132 | NA | NA | NA | NA |
| CFT_CT vs CT | 3 | 1 | 2.197228 | 0.674396 | 2.197228 | 0.674396 | NA | NA | NA | NA | NA | NA |
| CFT_CT vs EA | 0 | 0 | 0.689822 | 0.825471 | NA | NA | 0.689822 | 0.825471 | NA | NA | NA | NA |
| CFT_CT vs EA_CT | 0 | 0 | 0.431 | 0.812657 | NA | NA | 0.431 | 0.812657 | NA | NA | NA | NA |
| CFT_CT vs EMGBFB | 0 | 0 | 2.754597 | 1.637253 | NA | NA | 2.754597 | 1.637253 | NA | NA | NA | NA |
| CFT_CT vs EMGBFB_CT | 0 | 0 | -0.5754 | 0.874612 | NA | NA | -0.5754 | 0.874612 | NA | NA | NA | NA |
| CFT_CT vs FNS_CT | 0 | 0 | -2.31575 | 1.188936 | NA | NA | -2.31575 | 1.188936 | NA | NA | NA | NA |
| CFT_CT vs HBO | 0 | 0 | 2.183709 | 0.919084 | NA | NA | 2.183709 | 0.919084 | NA | NA | NA | NA |
| CFT_CT vs HBO_CT | 0 | 0 | -1.03709 | 0.726175 | NA | NA | -1.03709 | 0.726175 | NA | NA | NA | NA |
| CFT_CT vs MB | 0 | 0 | 0.93391 | 1.257192 | NA | NA | 0.93391 | 1.257192 | NA | NA | NA | NA |
| CFT_CT vs MB_CT | 0 | 0 | -1.12466 | 1.027287 | NA | NA | -1.12466 | 1.027287 | NA | NA | NA | NA |
| CFT_CT vs RT_CT | 0 | 0 | 0.410475 | 0.84534 | NA | NA | 0.410475 | 0.84534 | NA | NA | NA | NA |
| CFT_CT vs rTMS_ACUP_MB_CT | 0 | 0 | -0.76501 | 1.083097 | NA | NA | -0.76501 | 1.083097 | NA | NA | NA | NA |
| CFT_CT vs rTMS_CT | 0 | 0 | -1.77948 | 0.815759 | NA | NA | -1.77948 | 0.815759 | NA | NA | NA | NA |
| EA vs CT | 7 | 0.900249 | 1.507405 | 0.476018 | 1.365972 | 0.501697 | 2.783847 | 1.507181 | -1.41788 | 1.588488 | -0.89259 | 0.372074 |
| EA_CT vs CT | 8 | 0.879288 | 1.766228 | 0.453433 | 1.880137 | 0.483556 | 0.936486 | 1.305083 | 0.943651 | 1.391786 | 0.678014 | 0.497763 |
| EMGBFB vs CT | 0 | 0 | -0.55737 | 1.491908 | NA | NA | -0.55737 | 1.491908 | NA | NA | NA | NA |
| EMGBFB_CT vs CT | 4 | 1 | 2.772631 | 0.556899 | 2.772631 | 0.556899 | NA | NA | NA | NA | NA | NA |
| FNS_CT vs CT | 3 | 1 | 4.512982 | 0.979162 | 4.512982 | 0.979162 | NA | NA | NA | NA | NA | NA |
| HBO vs CT | 3 | 0.790255 | 0.013519 | 0.624424 | -0.53358 | 0.702418 | 2.074836 | 1.363433 | -2.60842 | 1.533734 | -1.7007 | 0.089 |
| HBO_CT vs CT | 21 | 1 | 3.234316 | 0.269296 | 3.234316 | 0.269296 | NA | NA | NA | NA | NA | NA |
| MB vs CT | 1 | 0.81845 | 1.263318 | 1.061 | 0.84 | 1.172788 | 3.171684 | 2.4901 | -2.33168 | 2.752459 | -0.84713 | 0.396924 |
| MB_CT vs CT | 3 | 1 | 3.321885 | 0.774925 | 3.321885 | 0.774925 | NA | NA | NA | NA | NA | NA |
| RT_CT vs CT | 5 | 0.899977 | 1.786753 | 0.509696 | 2.020047 | 0.537273 | -0.31235 | 1.611612 | 2.3324 | 1.69881 | 1.372961 | 0.169764 |
| rTMS_ACUP_MB_CT vs CT | 2 | 1 | 2.962233 | 0.847519 | 2.962233 | 0.847519 | NA | NA | NA | NA | NA | NA |
| rTMS_CT vs CT | 6 | 0.95102 | 3.976709 | 0.458969 | 4.090768 | 0.47064 | 1.762102 | 2.073824 | 2.328666 | 2.126557 | 1.09504 | 0.273499 |
| EA vs EA_CT | 4 | 0.63345 | -0.25882 | 0.552229 | -0.0211 | 0.693847 | -0.66965 | 0.912122 | 0.648552 | 1.146032 | 0.565911 | 0.571454 |
| EA vs EMGBFB | 0 | 0 | 2.064774 | 1.566008 | NA | NA | 2.064774 | 1.566008 | NA | NA | NA | NA |
| EA vs EMGBFB_CT | 0 | 0 | -1.26523 | 0.732618 | NA | NA | -1.26523 | 0.732618 | NA | NA | NA | NA |
| EA vs FNS_CT | 0 | 0 | -3.00558 | 1.088739 | NA | NA | -3.00558 | 1.088739 | NA | NA | NA | NA |
| EA vs HBO | 0 | 0 | 1.493887 | 0.785174 | NA | NA | 1.493887 | 0.785174 | NA | NA | NA | NA |
| EA vs HBO_CT | 0 | 0 | -1.72691 | 0.546912 | NA | NA | -1.72691 | 0.546912 | NA | NA | NA | NA |
| EA vs MB | 0 | 0 | 0.244087 | 1.16289 | NA | NA | 0.244087 | 1.16289 | NA | NA | NA | NA |
| EA vs MB_CT | 0 | 0 | -1.81448 | 0.909451 | NA | NA | -1.81448 | 0.909451 | NA | NA | NA | NA |
| EA vs RT_CT | 0 | 0 | -0.27935 | 0.697411 | NA | NA | -0.27935 | 0.697411 | NA | NA | NA | NA |
| EA vs rTMS_ACUP_MB_CT | 0 | 0 | -1.45483 | 0.97205 | NA | NA | -1.45483 | 0.97205 | NA | NA | NA | NA |
| EA vs rTMS_CT | 0 | 0 | -2.4693 | 0.648163 | NA | NA | -2.4693 | 0.648163 | NA | NA | NA | NA |
| EA_CT vs EMGBFB | 0 | 0 | 2.323597 | 1.559291 | NA | NA | 2.323597 | 1.559291 | NA | NA | NA | NA |
| EA_CT vs EMGBFB_CT | 0 | 0 | -1.0064 | 0.718149 | NA | NA | -1.0064 | 0.718149 | NA | NA | NA | NA |
| EA_CT vs FNS_CT | 0 | 0 | -2.74675 | 1.079055 | NA | NA | -2.74675 | 1.079055 | NA | NA | NA | NA |
| EA_CT vs HBO | 0 | 0 | 1.752709 | 0.77169 | NA | NA | 1.752709 | 0.77169 | NA | NA | NA | NA |
| EA_CT vs HBO_CT | 0 | 0 | -1.46809 | 0.527372 | NA | NA | -1.46809 | 0.527372 | NA | NA | NA | NA |
| EA_CT vs MB | 0 | 0 | 0.50291 | 1.153829 | NA | NA | 0.50291 | 1.153829 | NA | NA | NA | NA |
| EA_CT vs MB_CT | 0 | 0 | -1.55566 | 0.897836 | NA | NA | -1.55566 | 0.897836 | NA | NA | NA | NA |
| EA_CT vs RT_CT | 0 | 0 | -0.02053 | 0.682196 | NA | NA | -0.02053 | 0.682196 | NA | NA | NA | NA |
| EA_CT vs rTMS_ACUP_MB_CT | 0 | 0 | -1.19601 | 0.961192 | NA | NA | -1.19601 | 0.961192 | NA | NA | NA | NA |
| EA_CT vs rTMS_CT | 1 | 0.35614 | -2.21048 | 0.600744 | -2.3 | 1.006652 | -2.16096 | 0.748676 | -0.13904 | 1.254537 | -0.11083 | 0.911754 |
| EMGBFB vs EMGBFB_CT | 1 | 1 | -3.33 | 1.384071 | -3.33 | 1.384071 | NA | NA | NA | NA | NA | NA |
| EMGBFB vs FNS_CT | 0 | 0 | -5.07035 | 1.78453 | NA | NA | -5.07035 | 1.78453 | NA | NA | NA | NA |
| EMGBFB vs HBO | 0 | 0 | -0.57089 | 1.617311 | NA | NA | -0.57089 | 1.617311 | NA | NA | NA | NA |
| EMGBFB vs HBO_CT | 0 | 0 | -3.79168 | 1.516017 | NA | NA | -3.79168 | 1.516017 | NA | NA | NA | NA |
| EMGBFB vs MB | 0 | 0 | -1.82069 | 1.830713 | NA | NA | -1.82069 | 1.830713 | NA | NA | NA | NA |
| EMGBFB vs MB_CT | 0 | 0 | -3.87925 | 1.68116 | NA | NA | -3.87925 | 1.68116 | NA | NA | NA | NA |
| EMGBFB vs RT_CT | 0 | 0 | -2.34412 | 1.576572 | NA | NA | -2.34412 | 1.576572 | NA | NA | NA | NA |
| EMGBFB vs rTMS_ACUP_MB_CT | 0 | 0 | -3.5196 | 1.715831 | NA | NA | -3.5196 | 1.715831 | NA | NA | NA | NA |
| EMGBFB vs rTMS_CT | 0 | 0 | -4.53408 | 1.56091 | NA | NA | -4.53408 | 1.56091 | NA | NA | NA | NA |
| EMGBFB_CT vs FNS_CT | 0 | 0 | -1.74035 | 1.126453 | NA | NA | -1.74035 | 1.126453 | NA | NA | NA | NA |
| EMGBFB_CT vs HBO | 0 | 0 | 2.759112 | 0.836685 | NA | NA | 2.759112 | 0.836685 | NA | NA | NA | NA |
| EMGBFB_CT vs HBO_CT | 0 | 0 | -0.46168 | 0.618593 | NA | NA | -0.46168 | 0.618593 | NA | NA | NA | NA |
| EMGBFB_CT vs MB | 0 | 0 | 1.509313 | 1.198273 | NA | NA | 1.509313 | 1.198273 | NA | NA | NA | NA |
| EMGBFB_CT vs MB_CT | 0 | 0 | -0.54925 | 0.954278 | NA | NA | -0.54925 | 0.954278 | NA | NA | NA | NA |
| EMGBFB_CT vs RT_CT | 0 | 0 | 0.985878 | 0.754935 | NA | NA | 0.985878 | 0.754935 | NA | NA | NA | NA |
| EMGBFB_CT vs rTMS_ACUP_MB_CT | 0 | 0 | -0.1896 | 1.014113 | NA | NA | -0.1896 | 1.014113 | NA | NA | NA | NA |
| EMGBFB_CT vs rTMS_CT | 0 | 0 | -1.20408 | 0.721657 | NA | NA | -1.20408 | 0.721657 | NA | NA | NA | NA |
| FNS_CT vs HBO | 0 | 0 | 4.499464 | 1.16132 | NA | NA | 4.499464 | 1.16132 | NA | NA | NA | NA |
| FNS_CT vs HBO_CT | 0 | 0 | 1.278667 | 1.015519 | NA | NA | 1.278667 | 1.015519 | NA | NA | NA | NA |
| FNS_CT vs MB | 0 | 0 | 3.249664 | 1.443773 | NA | NA | 3.249664 | 1.443773 | NA | NA | NA | NA |
| FNS_CT vs MB_CT | 0 | 0 | 1.191097 | 1.248707 | NA | NA | 1.191097 | 1.248707 | NA | NA | NA | NA |
| FNS_CT vs RT_CT | 0 | 0 | 2.72623 | 1.103879 | NA | NA | 2.72623 | 1.103879 | NA | NA | NA | NA |
| FNS_CT vs rTMS_ACUP_MB_CT | 0 | 0 | 1.55075 | 1.295009 | NA | NA | 1.55075 | 1.295009 | NA | NA | NA | NA |
| FNS_CT vs rTMS_CT | 0 | 0 | 0.536273 | 1.081393 | NA | NA | 0.536273 | 1.081393 | NA | NA | NA | NA |
| HBO vs HBO_CT | 3 | 0.797545 | -3.2208 | 0.623744 | -2.68633 | 0.698439 | -5.32625 | 1.386253 | 2.639912 | 1.552261 | 1.700688 | 0.089002 |
| HBO vs MB | 0 | 0 | -1.2498 | 1.231108 | NA | NA | -1.2498 | 1.231108 | NA | NA | NA | NA |
| HBO vs MB_CT | 0 | 0 | -3.30837 | 0.995195 | NA | NA | -3.30837 | 0.995195 | NA | NA | NA | NA |
| HBO vs RT_CT | 0 | 0 | -1.77323 | 0.806036 | NA | NA | -1.77323 | 0.806036 | NA | NA | NA | NA |
| HBO vs rTMS_ACUP_MB_CT | 0 | 0 | -2.94871 | 1.052708 | NA | NA | -2.94871 | 1.052708 | NA | NA | NA | NA |
| HBO vs rTMS_CT | 0 | 0 | -3.96319 | 0.774956 | NA | NA | -3.96319 | 0.774956 | NA | NA | NA | NA |
| HBO_CT vs MB | 0 | 0 | 1.970998 | 1.094642 | NA | NA | 1.970998 | 1.094642 | NA | NA | NA | NA |
| HBO_CT vs MB_CT | 0 | 0 | -0.08757 | 0.820384 | NA | NA | -0.08757 | 0.820384 | NA | NA | NA | NA |
| HBO_CT vs RT_CT | 0 | 0 | 1.447563 | 0.576463 | NA | NA | 1.447563 | 0.576463 | NA | NA | NA | NA |
| HBO_CT vs rTMS_ACUP_MB_CT | 0 | 0 | 0.272083 | 0.889274 | NA | NA | 0.272083 | 0.889274 | NA | NA | NA | NA |
| HBO_CT vs rTMS_CT | 0 | 0 | -0.74239 | 0.53214 | NA | NA | -0.74239 | 0.53214 | NA | NA | NA | NA |
| MB vs MB_CT | 0 | 0 | -2.05857 | 1.313861 | NA | NA | -2.05857 | 1.313861 | NA | NA | NA | NA |
| MB vs RT_CT | 0 | 0 | -0.52343 | 1.177077 | NA | NA | -0.52343 | 1.177077 | NA | NA | NA | NA |
| MB vs rTMS_ACUP_MB_CT | 0 | 0 | -1.69891 | 1.357943 | NA | NA | -1.69891 | 1.357943 | NA | NA | NA | NA |
| MB vs rTMS_CT | 0 | 0 | -2.71339 | 1.156016 | NA | NA | -2.71339 | 1.156016 | NA | NA | NA | NA |
| MB_CT vs RT_CT | 0 | 0 | 1.535133 | 0.927523 | NA | NA | 1.535133 | 0.927523 | NA | NA | NA | NA |
| MB_CT vs rTMS_ACUP_MB_CT | 0 | 0 | 0.359652 | 1.148389 | NA | NA | 0.359652 | 1.148389 | NA | NA | NA | NA |
| MB_CT vs rTMS_CT | 0 | 0 | -0.65482 | 0.900645 | NA | NA | -0.65482 | 0.900645 | NA | NA | NA | NA |
| RT_CT vs rTMS_ACUP_MB_CT | 0 | 0 | -1.17548 | 0.988978 | NA | NA | -1.17548 | 0.988978 | NA | NA | NA | NA |
| RT_CT vs rTMS_CT | 0 | 0 | -2.18996 | 0.685888 | NA | NA | -2.18996 | 0.685888 | NA | NA | NA | NA |
| rTMS_ACUP_MB_CT vs rTMS_CT | 0 | 0 | -1.01448 | 0.963816 | NA | NA | -1.01448 | 0.963816 | NA | NA | NA | NA |

NA not available, k Number of studies providing direct evidence, prop Direct evidence proportion, nma Estimated treatment effect (MD) in network meta-analysis, direct Estimated treatment effect (MD) derived from direct evidence, indir. Estimated treatment effect (MD) derived from indirect evidence, Diff Difference between direct and indirect treatment estimates, z z-value of test for disagreement (direct versus indirect), p p-value of test for disagreement (direct versus indirect), yellow is 0.05-0.1.

Table 7.2.3 Details of SIDE splitting results （ADL）

| comparison | k | prop | NMA | | Direct | | Indir | | Diff | | z | p |
| --- | --- | --- | --- | --- | --- | --- | --- | --- | --- | --- | --- | --- |
|  |  |  | TE | seTE | TE | seTE | TE | seTE | TE | seTE |  |  |
| ACUP_CT vs ACUP_MB_CT | 1 | 1 | -5.98 | 1.787795 | -5.98 | 1.787795 | NA | NA | NA | NA | NA | NA |
| ACUP_CT vs ACUP_RT_CT | 0 | 0 | -3.09593 | 3.171708 | NA | NA | -3.09593 | 3.171708 | NA | NA | NA | NA |
| ACUP_CT vs AE_CT | 0 | 0 | 0.798706 | 2.095566 | NA | NA | 0.798706 | 2.095566 | NA | NA | NA | NA |
| ACUP_CT vs CFT_CT | 0 | 0 | 7.353958 | 1.61043 | NA | NA | 7.353958 | 1.61043 | NA | NA | NA | NA |
| ACUP_CT vs CT | 4 | 1 | 11.22871 | 1.002189 | 11.22871 | 1.002189 | NA | NA | NA | NA | NA | NA |
| ACUP_CT vs EA | 0 | 0 | 6.494189 | 2.04796 | NA | NA | 6.494189 | 2.04796 | NA | NA | NA | NA |
| ACUP_CT vs EA_CT | 0 | 0 | 7.009347 | 1.674427 | NA | NA | 7.009347 | 1.674427 | NA | NA | NA | NA |
| ACUP_CT vs EMGBFB_CT | 0 | 0 | 1.054135 | 1.453515 | NA | NA | 1.054135 | 1.453515 | NA | NA | NA | NA |
| ACUP_CT vs FNS_CT | 0 | 0 | 3.68457 | 3.37911 | NA | NA | 3.68457 | 3.37911 | NA | NA | NA | NA |
| ACUP_CT vs HBO_CT | 0 | 0 | 2.282792 | 1.382038 | NA | NA | 2.282792 | 1.382038 | NA | NA | NA | NA |
| ACUP_CT vs RT_CT | 0 | 0 | 4.034067 | 1.924534 | NA | NA | 4.034067 | 1.924534 | NA | NA | NA | NA |
| ACUP_CT vs rTMS_CT | 0 | 0 | -0.60444 | 1.400665 | NA | NA | -0.60444 | 1.400665 | NA | NA | NA | NA |
| ACUP_MB_CT vs ACUP_RT_CT | 0 | 0 | 2.884073 | 3.640871 | NA | NA | 2.884073 | 3.640871 | NA | NA | NA | NA |
| ACUP_MB_CT vs AE_CT | 0 | 0 | 6.778709 | 2.754561 | NA | NA | 6.778709 | 2.754561 | NA | NA | NA | NA |
| ACUP_MB_CT vs CFT_CT | 0 | 0 | 13.33396 | 2.406178 | NA | NA | 13.33396 | 2.406178 | NA | NA | NA | NA |
| ACUP_MB_CT vs CT | 0 | 0 | 17.20871 | 2.049535 | NA | NA | 17.20871 | 2.049535 | NA | NA | NA | NA |
| ACUP_MB_CT vs EA | 0 | 0 | 12.47419 | 2.71852 | NA | NA | 12.47419 | 2.71852 | NA | NA | NA | NA |
| ACUP_MB_CT vs EA_CT | 0 | 0 | 12.98935 | 2.449473 | NA | NA | 12.98935 | 2.449473 | NA | NA | NA | NA |
| ACUP_MB_CT vs EMGBFB_CT | 0 | 0 | 7.034139 | 2.304108 | NA | NA | 7.034139 | 2.304108 | NA | NA | NA | NA |
| ACUP_MB_CT vs FNS_CT | 0 | 0 | 9.664573 | 3.822904 | NA | NA | 9.664573 | 3.822904 | NA | NA | NA | NA |
| ACUP_MB_CT vs HBO_CT | 0 | 0 | 8.262796 | 2.259699 | NA | NA | 8.262796 | 2.259699 | NA | NA | NA | NA |
| ACUP_MB_CT vs RT_CT | 0 | 0 | 10.01407 | 2.626793 | NA | NA | 10.01407 | 2.626793 | NA | NA | NA | NA |
| ACUP_MB_CT vs rTMS_CT | 0 | 0 | 5.375561 | 2.271139 | NA | NA | 5.375561 | 2.271139 | NA | NA | NA | NA |
| ACUP_RT_CT vs AE_CT | 0 | 0 | 3.894636 | 3.527374 | NA | NA | 3.894636 | 3.527374 | NA | NA | NA | NA |
| ACUP_RT_CT vs CFT_CT | 0 | 0 | 10.44989 | 3.262584 | NA | NA | 10.44989 | 3.262584 | NA | NA | NA | NA |
| ACUP_RT_CT vs CT | 0 | 0 | 14.32464 | 3.009211 | NA | NA | 14.32464 | 3.009211 | NA | NA | NA | NA |
| ACUP_RT_CT vs EA | 0 | 0 | 9.59012 | 3.499301 | NA | NA | 9.59012 | 3.499301 | NA | NA | NA | NA |
| ACUP_RT_CT vs EA_CT | 0 | 0 | 10.10528 | 3.294643 | NA | NA | 10.10528 | 3.294643 | NA | NA | NA | NA |
| ACUP_RT_CT vs EMGBFB_CT | 0 | 0 | 4.150066 | 3.188052 | NA | NA | 4.150066 | 3.188052 | NA | NA | NA | NA |
| ACUP_RT_CT vs FNS_CT | 0 | 0 | 6.780501 | 4.412409 | NA | NA | 6.780501 | 4.412409 | NA | NA | NA | NA |
| ACUP_RT_CT vs HBO_CT | 0 | 0 | 5.378723 | 3.156105 | NA | NA | 5.378723 | 3.156105 | NA | NA | NA | NA |
| ACUP_RT_CT vs RT_CT | 1 | 1 | 7.129997 | 2.521091 | 7.129997 | 2.521091 | NA | NA | NA | NA | NA | NA |
| ACUP_RT_CT vs rTMS_CT | 0 | 0 | 2.491488 | 3.164306 | NA | NA | 2.491488 | 3.164306 | NA | NA | NA | NA |
| AE_CT vs CFT_CT | 0 | 0 | 6.555252 | 2.230721 | NA | NA | 6.555252 | 2.230721 | NA | NA | NA | NA |
| AE_CT vs CT | 1 | 1 | 10.43 | 1.840384 | 10.43 | 1.840384 | NA | NA | NA | NA | NA | NA |
| AE_CT vs EA | 0 | 0 | 5.695483 | 2.564522 | NA | NA | 5.695483 | 2.564522 | NA | NA | NA | NA |
| AE_CT vs EA_CT | 0 | 0 | 6.210641 | 2.277354 | NA | NA | 6.210641 | 2.277354 | NA | NA | NA | NA |
| AE_CT vs EMGBFB_CT | 0 | 0 | 0.25543 | 2.120221 | NA | NA | 0.25543 | 2.120221 | NA | NA | NA | NA |
| AE_CT vs FNS_CT | 0 | 0 | 2.885864 | 3.714972 | NA | NA | 2.885864 | 3.714972 | NA | NA | NA | NA |
| AE_CT vs HBO_CT | 0 | 0 | 1.484086 | 2.071874 | NA | NA | 1.484086 | 2.071874 | NA | NA | NA | NA |
| AE_CT vs RT_CT | 0 | 0 | 3.235361 | 2.467076 | NA | NA | 3.235361 | 2.467076 | NA | NA | NA | NA |
| AE_CT vs rTMS_CT | 0 | 0 | -1.40315 | 2.084345 | NA | NA | -1.40315 | 2.084345 | NA | NA | NA | NA |
| CFT_CT vs CT | 3 | 1 | 3.874748 | 1.260596 | 3.874748 | 1.260596 | NA | NA | NA | NA | NA | NA |
| CFT_CT vs EA | 0 | 0 | -0.85977 | 2.186061 | NA | NA | -0.85977 | 2.186061 | NA | NA | NA | NA |
| CFT_CT vs EA_CT | 0 | 0 | -0.34461 | 1.840768 | NA | NA | -0.34461 | 1.840768 | NA | NA | NA | NA |
| CFT_CT vs EMGBFB_CT | 0 | 0 | -6.29982 | 1.642384 | NA | NA | -6.29982 | 1.642384 | NA | NA | NA | NA |
| CFT_CT vs FNS_CT | 0 | 0 | -3.66939 | 3.464549 | NA | NA | -3.66939 | 3.464549 | NA | NA | NA | NA |
| CFT_CT vs HBO_CT | 0 | 0 | -5.07117 | 1.579477 | NA | NA | -5.07117 | 1.579477 | NA | NA | NA | NA |
| CFT_CT vs RT_CT | 0 | 0 | -3.31989 | 2.070882 | NA | NA | -3.31989 | 2.070882 | NA | NA | NA | NA |
| CFT_CT vs rTMS_CT | 0 | 0 | -7.9584 | 1.595801 | NA | NA | -7.9584 | 1.595801 | NA | NA | NA | NA |
| EA vs CT | 2 | 1 | 4.734517 | 1.78599 | 4.734517 | 1.78599 | NA | NA | NA | NA | NA | NA |
| EA_CT vs CT | 1 | 0.864715 | 4.219359 | 1.341389 | 4.799999 | 1.442508 | 0.508022 | 3.646951 | 4.291977 | 3.921872 | 1.094369 | 0.273793 |
| EMGBFB_CT vs CT | 3 | 1 | 10.17457 | 1.052769 | 10.17457 | 1.052769 | NA | NA | NA | NA | NA | NA |
| FNS_CT vs CT | 2 | 1 | 7.544136 | 3.227073 | 7.544136 | 3.227073 | NA | NA | NA | NA | NA | NA |
| HBO_CT vs CT | 4 | 1 | 8.945914 | 0.951654 | 8.945914 | 0.951654 | NA | NA | NA | NA | NA | NA |
| RT_CT vs CT | 3 | 1 | 7.194639 | 1.643 | 7.194639 | 1.643 | NA | NA | NA | NA | NA | NA |
| rTMS_CT vs CT | 3 | 1 | 11.83315 | 0.978508 | 11.83315 | 0.978508 | NA | NA | NA | NA | NA | NA |
| EA vs EA_CT | 0 | 0 | 0.515158 | 2.233626 | NA | NA | 0.515158 | 2.233626 | NA | NA | NA | NA |
| EA vs EMGBFB_CT | 0 | 0 | -5.44005 | 2.073182 | NA | NA | -5.44005 | 2.073182 | NA | NA | NA | NA |
| EA vs FNS_CT | 0 | 0 | -2.80962 | 3.688328 | NA | NA | -2.80962 | 3.688328 | NA | NA | NA | NA |
| EA vs HBO_CT | 0 | 0 | -4.2114 | 2.023711 | NA | NA | -4.2114 | 2.023711 | NA | NA | NA | NA |
| EA vs RT_CT | 0 | 0 | -2.46012 | 2.426769 | NA | NA | -2.46012 | 2.426769 | NA | NA | NA | NA |
| EA vs rTMS_CT | 0 | 0 | -7.09863 | 2.036477 | NA | NA | -7.09863 | 2.036477 | NA | NA | NA | NA |
| EA_CT vs EMGBFB_CT | 0 | 0 | -5.95521 | 1.705182 | NA | NA | -5.95521 | 1.705182 | NA | NA | NA | NA |
| EA_CT vs FNS_CT | 0 | 0 | -3.32478 | 3.494757 | NA | NA | -3.32478 | 3.494757 | NA | NA | NA | NA |
| EA_CT vs HBO_CT | 0 | 0 | -4.72655 | 1.644679 | NA | NA | -4.72655 | 1.644679 | NA | NA | NA | NA |
| EA_CT vs RT_CT | 0 | 0 | -2.97528 | 2.121031 | NA | NA | -2.97528 | 2.121031 | NA | NA | NA | NA |
| EA_CT vs rTMS_CT | 1 | 0.862766 | -7.61379 | 1.343092 | -8.2 | 1.44597 | -3.92839 | 3.625556 | -4.27162 | 3.903266 | -1.09437 | 0.273793 |
| EMGBFB_CT vs FNS_CT | 0 | 0 | 2.630434 | 3.394455 | NA | NA | 2.630434 | 3.394455 | NA | NA | NA | NA |
| EMGBFB_CT vs HBO_CT | 0 | 0 | 1.228657 | 1.419144 | NA | NA | 1.228657 | 1.419144 | NA | NA | NA | NA |
| EMGBFB_CT vs RT_CT | 0 | 0 | 2.979931 | 1.951351 | NA | NA | 2.979931 | 1.951351 | NA | NA | NA | NA |
| EMGBFB_CT vs rTMS_CT | 0 | 0 | -1.65858 | 1.43729 | NA | NA | -1.65858 | 1.43729 | NA | NA | NA | NA |
| FNS_CT vs HBO_CT | 0 | 0 | -1.40178 | 3.364468 | NA | NA | -1.40178 | 3.364468 | NA | NA | NA | NA |
| FNS_CT vs RT_CT | 0 | 0 | 0.349497 | 3.62125 | NA | NA | 0.349497 | 3.62125 | NA | NA | NA | NA |
| FNS_CT vs rTMS_CT | 0 | 0 | -4.28901 | 3.372162 | NA | NA | -4.28901 | 3.372162 | NA | NA | NA | NA |
| HBO_CT vs RT_CT | 0 | 0 | 1.751274 | 1.898709 | NA | NA | 1.751274 | 1.898709 | NA | NA | NA | NA |
| HBO_CT vs rTMS_CT | 0 | 0 | -2.88723 | 1.364963 | NA | NA | -2.88723 | 1.364963 | NA | NA | NA | NA |
| RT_CT vs rTMS_CT | 0 | 0 | -4.63851 | 1.91231 | NA | NA | -4.63851 | 1.91231 | NA | NA | NA | NA |

# Appendix 8: Network Meta-Regression

Table 8 Network Meta-Regression

| Outcomes | Shared beta (median and 95% CI) | | | | | | | |
| --- | --- | --- | --- | --- | --- | --- | --- | --- |
|  | year | sample | male | age | duration | period | frequency | time |
| MMSE | 0.07 (-0.64; 0.79) | -0.81 (-1.44; -0.17)* | -0.38 (-1.06; 0.26) | -0.38 (-1.06; 0.26) | 0.01 (-0.52; 0.56) | -0.05 (-0.76; 0.65) | 0.18 (-0.84; 1.24) | -1.03 (-2.00; -0,06) * |
| ADL | -2.34 (-5.51; 1.13) | -2.18 (-5.24; 1.70) | -0.99 (-4.49; 2.63) | 0.32 (-1.95; 2.49) | -1.96 (-3.32; -0.31)* | 1.22 (-1.77; 4.25) | -0.10 (-5.95; 6.15) | 2.96 (-0.52; 6.20) |

CI: Credible Interval; *: Significant influence factors, 95% CI does not contain zero.

## 8.1 MMSE

**8.1.1 Publication year**

When the model was adjusted for centering value of publish year 2015, compared with the control group, the MD value of non-pharmacological interventions types did not change significantly, and the hierarchy from the unadjusted model largely retained.


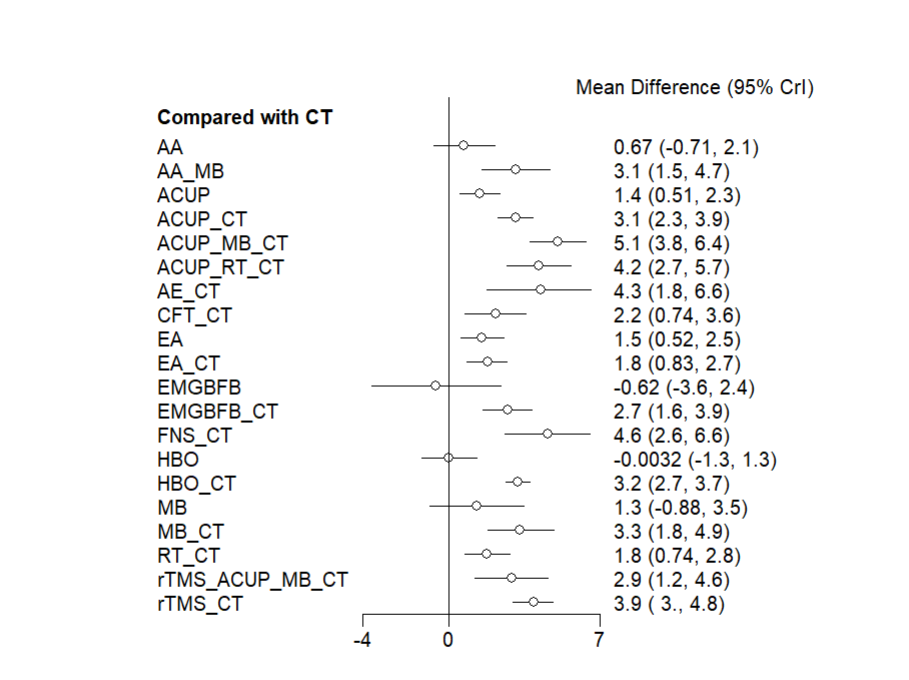


Figure 8.1.1: Forest plot adjusted for Publication year. AA auricular acupuncture, ACUP acupuncture, AE Aerobic exercise, CFT Cognitive function training, CT Conventional treatment, EMGBFB electromyographic biofeedback, EA electroacupunctur, FNS Fastigial nucleus stimulation, HBO hyperbaric oxygen therapy, MB moxibustion, RT Rehabilitation training, rTMS Repetitive Transcranial Magnetic Stimulation.

**8.1.2 Sample Size**

When the model was adjusted for centering value of publish sample 84, compared with the control group, the MD value of non-pharmacological interventions types did not change significantly, and the hierarchy from the unadjusted model largely retained.


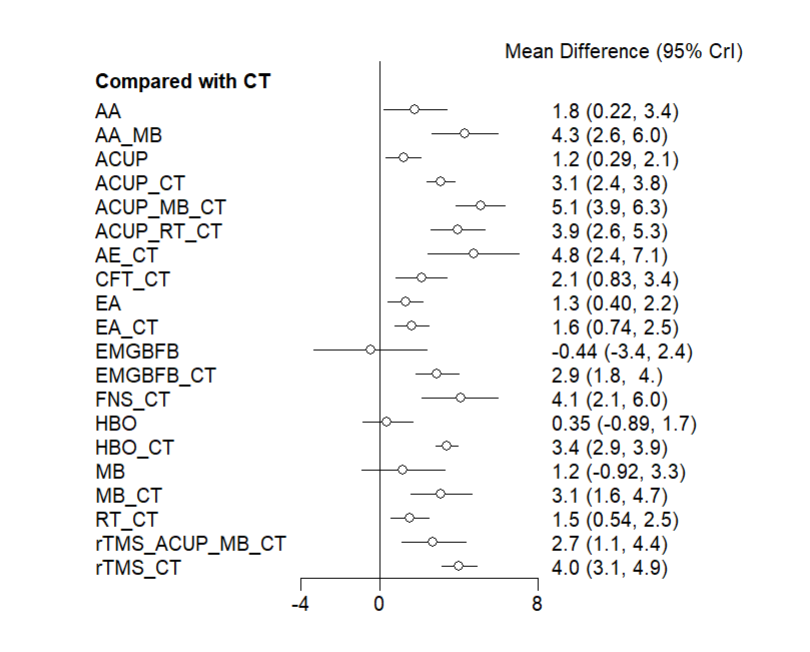


Figure 8.1.2: Forest plot adjusted for Sample Size. AA auricular acupuncture, ACUP acupuncture, AE Aerobic exercise, CFT Cognitive function training, CT Conventional treatment, EMGBFB electromyographic biofeedback, EA electroacupunctur, FNS Fastigial nucleus stimulation, HBO hyperbaric oxygen therapy, MB moxibustion, RT Rehabilitation training, rTMS Repetitive Transcranial Magnetic Stimulation.

**8.1.3** **Percentage Male**

When the model was adjusted for centering value of publish male 46, compared with the control group, the MD value of non-pharmacological interventions types did not change significantly, and the hierarchy from the unadjusted model largely retained.


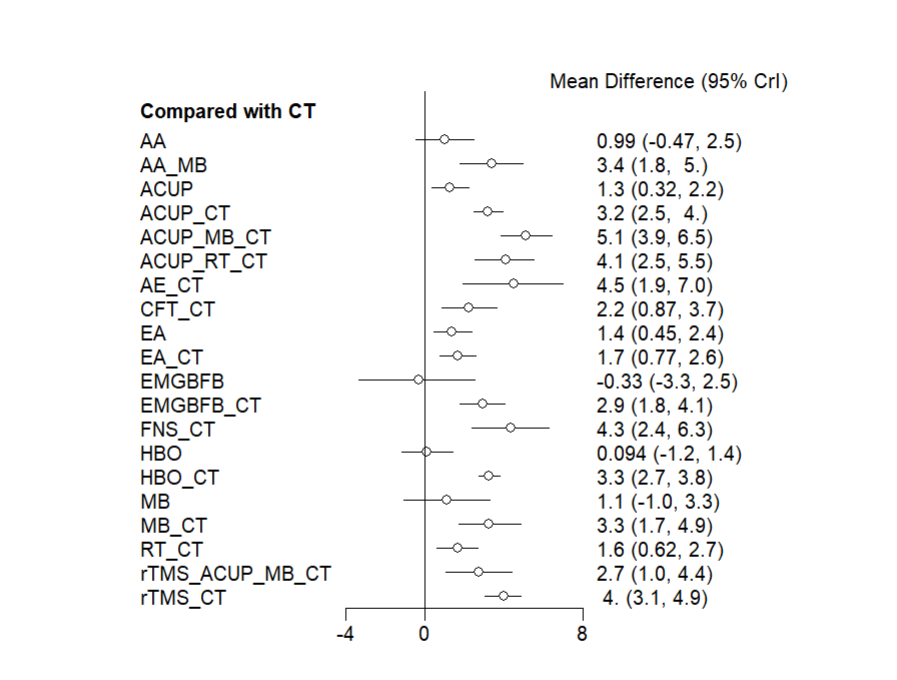
 Figure 8.1.3: Forest plot adjusted for Percentage Male. AA auricular acupuncture, ACUP acupuncture, AE Aerobic exercise, CFT Cognitive function training, CT Conventional treatment, EMGBFB electromyographic biofeedback, EA electroacupunctur, FNS Fastigial nucleus stimulation, HBO hyperbaric oxygen therapy, MB moxibustion, RT Rehabilitation training, rTMS Repetitive Transcranial Magnetic Stimulation.

**8.1.4** **Mean Age**

When the model was adjusted for centering value of publish age 67, compared with the control group, the MD value of non-pharmacological interventions types did not change significantly, and the hierarchy from the unadjusted model largely retained.


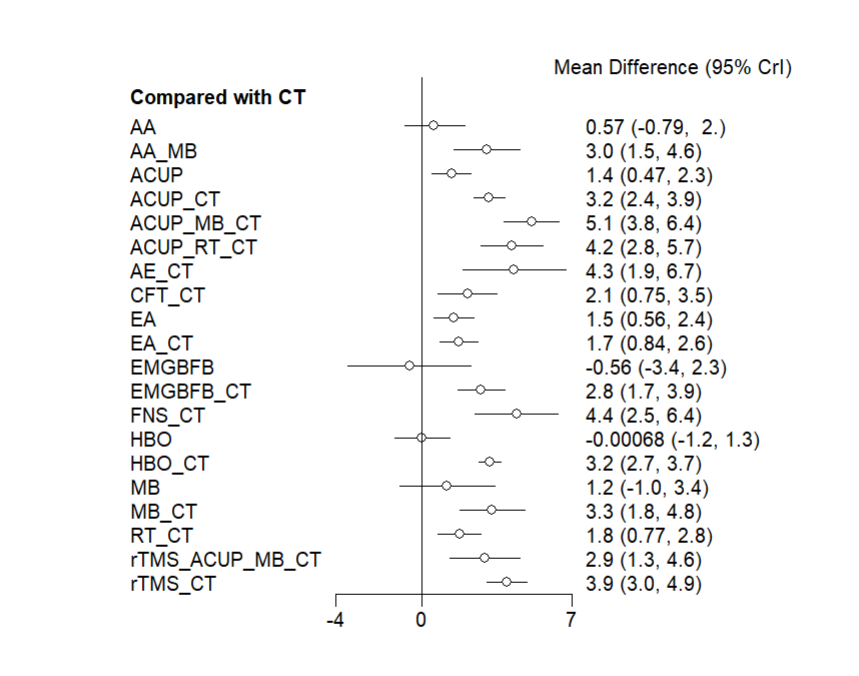


Figure 8.1.4: Forest plot adjusted for Mean Age. AA auricular acupuncture, ACUP acupuncture, AE Aerobic exercise, CFT Cognitive function training, CT Conventional treatment, EMGBFB electromyographic biofeedback, EA electroacupunctur, FNS Fastigial nucleus stimulation, HBO hyperbaric oxygen therapy, MB moxibustion, RT Rehabilitation training, rTMS Repetitive Transcranial Magnetic Stimulation.

**8.1.5** **Disease duration**

When the model was adjusted for centering value of publish duration 19, compared with the control group, the MD value of non-pharmacological interventions types did not change significantly, and the hierarchy from the unadjusted model largely retained.


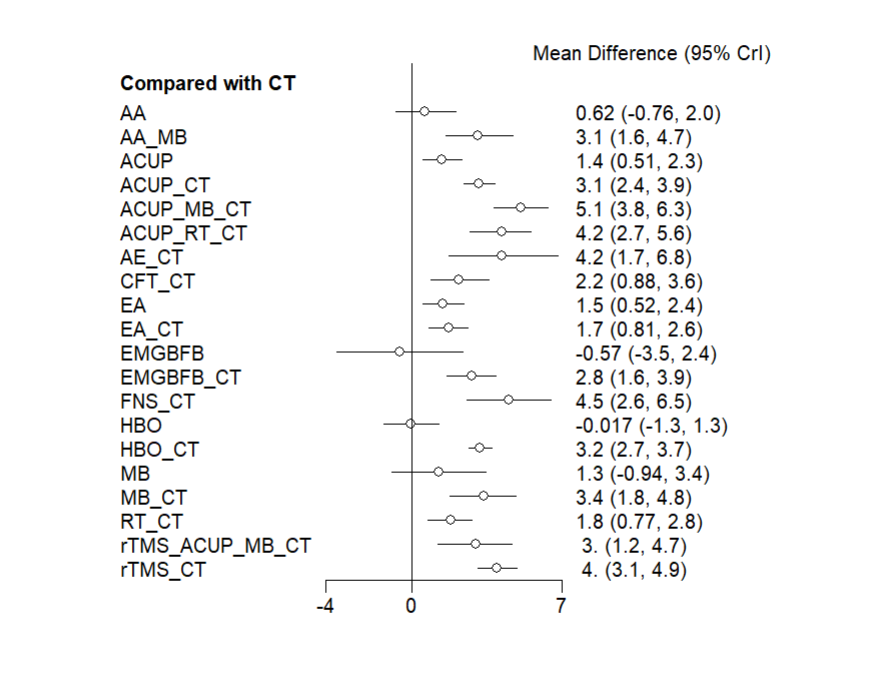


Figure 8.1.5: Forest plot adjusted for Disease duration. AA auricular acupuncture, ACUP acupuncture, AE Aerobic exercise, CFT Cognitive function training, CT Conventional treatment, EMGBFB electromyographic biofeedback, EA electroacupunctur, FNS Fastigial nucleus stimulation, HBO hyperbaric oxygen therapy, MB moxibustion, RT Rehabilitation training, rTMS Repetitive Transcranial Magnetic Stimulation.

**8.1.6 Treatment period**

When the model was adjusted for centering value of publish period 8, compared with the control group, the MD value of non-pharmacological interventions types did not change significantly, and the hierarchy from the unadjusted model largely retained.


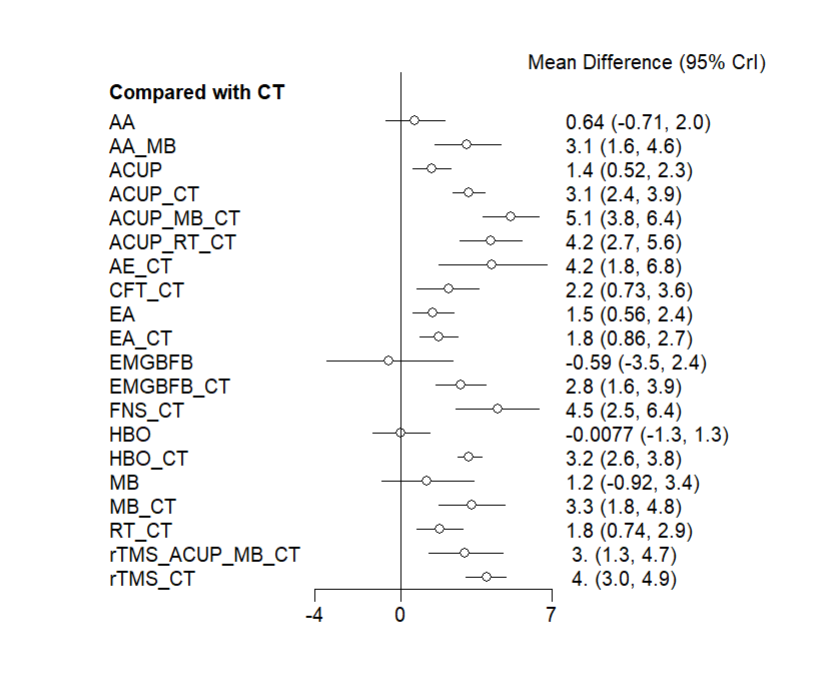


Figure 8.1.6: Forest plot adjusted for Treatment period. AA auricular acupuncture, ACUP acupuncture, AE Aerobic exercise, CFT Cognitive function training, CT Conventional treatment, EMGBFB electromyographic biofeedback, EA electroacupunctur, FNS Fastigial nucleus stimulation, HBO hyperbaric oxygen therapy, MB moxibustion, RT Rehabilitation training, rTMS Repetitive Transcranial Magnetic Stimulation.

**8.1.7 Treatment Frequency**

When the model was adjusted for centering value of publish frequency 6, compared with the control group, the MD value of non-pharmacological interventions types did not change significantly, and the hierarchy from the unadjusted model largely retained.


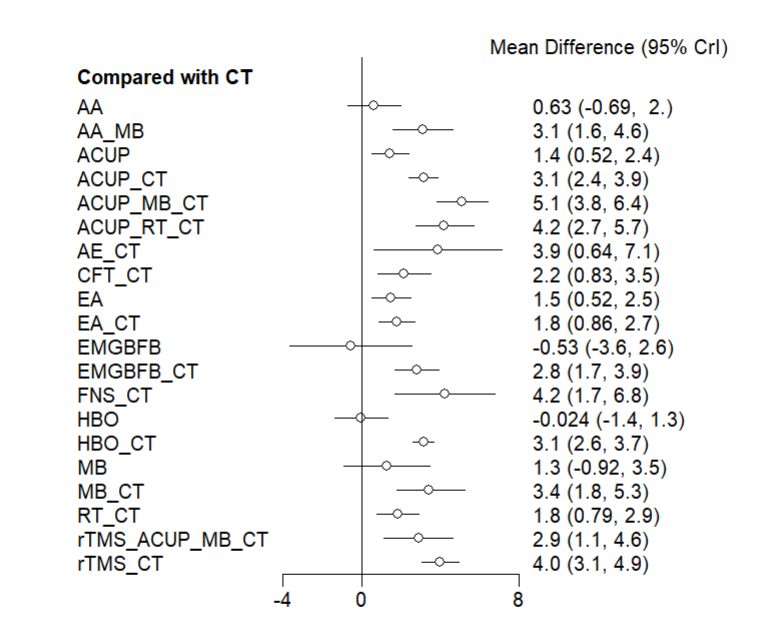


Figure 8.1.7: Forest plot adjusted for Treatment Frequency. AA auricular acupuncture, ACUP acupuncture, AE Aerobic exercise, CFT Cognitive function training, CT Conventional treatment, EMGBFB electromyographic biofeedback, EA electroacupunctur, FNS Fastigial nucleus stimulation, HBO hyperbaric oxygen therapy, MB moxibustion, RT Rehabilitation training, rTMS Repetitive Transcranial Magnetic Stimulation.

**8.1.8 Treatment time**

When the model was adjusted for centering value of publish time 46 min, compared with the control group, the MD value of non-pharmacological interventions types did not change significantly, and the hierarchy from the unadjusted model largely retained.


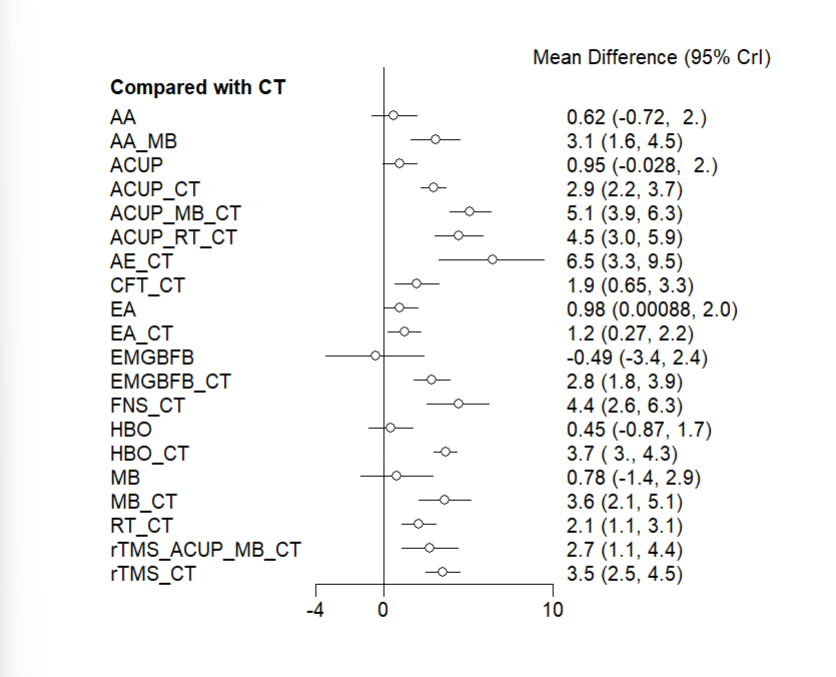


Figure 8.1.8: Forest plot adjusted for Treatment time. AA auricular acupuncture, ACUP acupuncture, AE Aerobic exercise, CFT Cognitive function training, CT Conventional treatment, EMGBFB electromyographic biofeedback, EA electroacupunctur, FNS Fastigial nucleus stimulation, HBO hyperbaric oxygen therapy, MB moxibustion, RT Rehabilitation training, rTMS Repetitive Transcranial Magnetic Stimulation.

## 8.2 ADL

**8.2.1** **Publication year**

When the model was adjusted for centering value of publish year 2017, compared with the control group, the MD value of non-pharmacological interventions types did not change significantly, and the hierarchy from the unadjusted model largely retained.


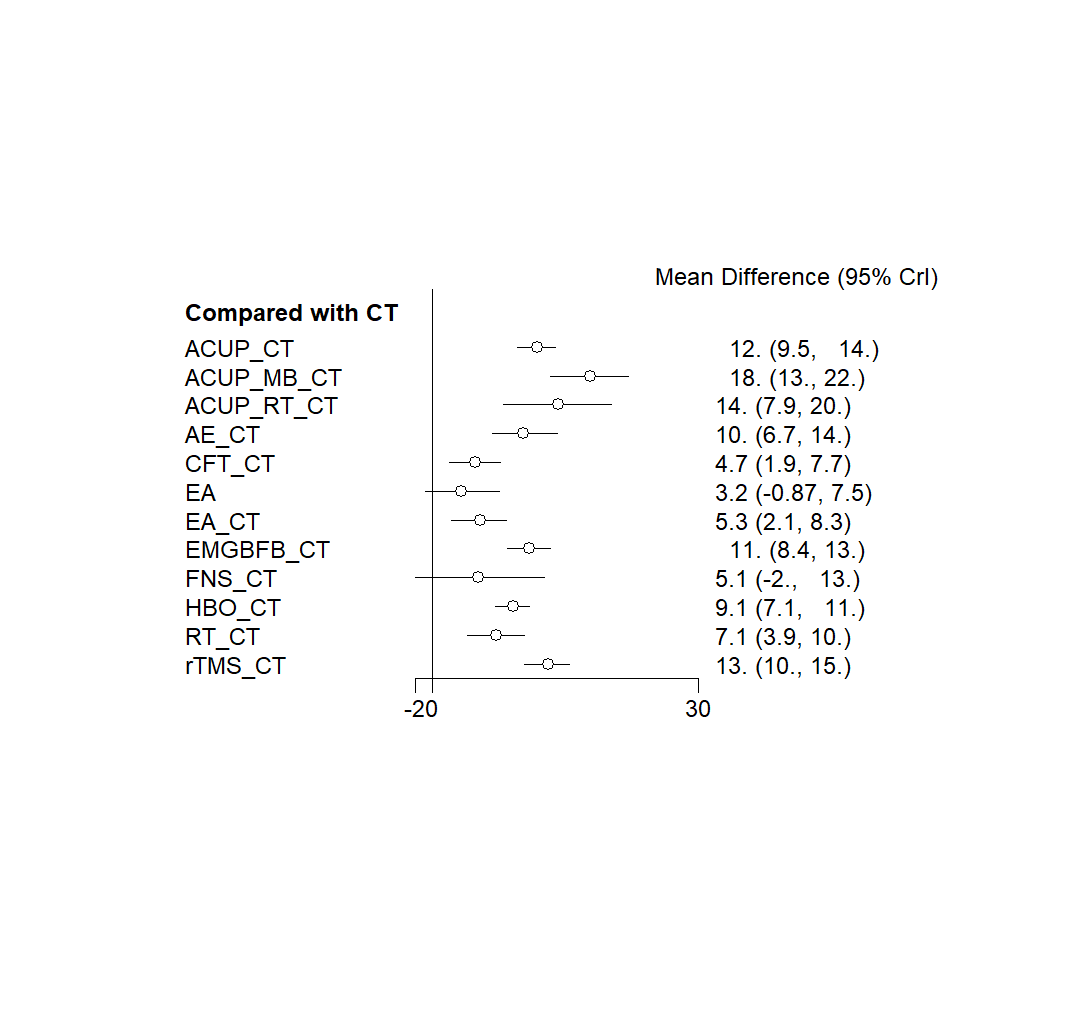


Figure 8.2.1: Forest plot adjusted for Publication year. ACUP acupuncture, AE Aerobic exercise, CFT Cognitive function training, CT Conventional treatment, EMGBFB electromyographic biofeedback, EA electroacupunctur, FNS Fastigial nucleus stimulation, HBO hyperbaric oxygen therapy, MB moxibustion, RT Rehabilitation training, rTMS Repetitive Transcranial Magnetic Stimulation.

**8.2.2 Sample Size**

When the model was adjusted for centering value of publish sample 80, compared with the control group, the MD value of non-pharmacological interventions types did not change significantly, and the hierarchy from the unadjusted model largely retained.


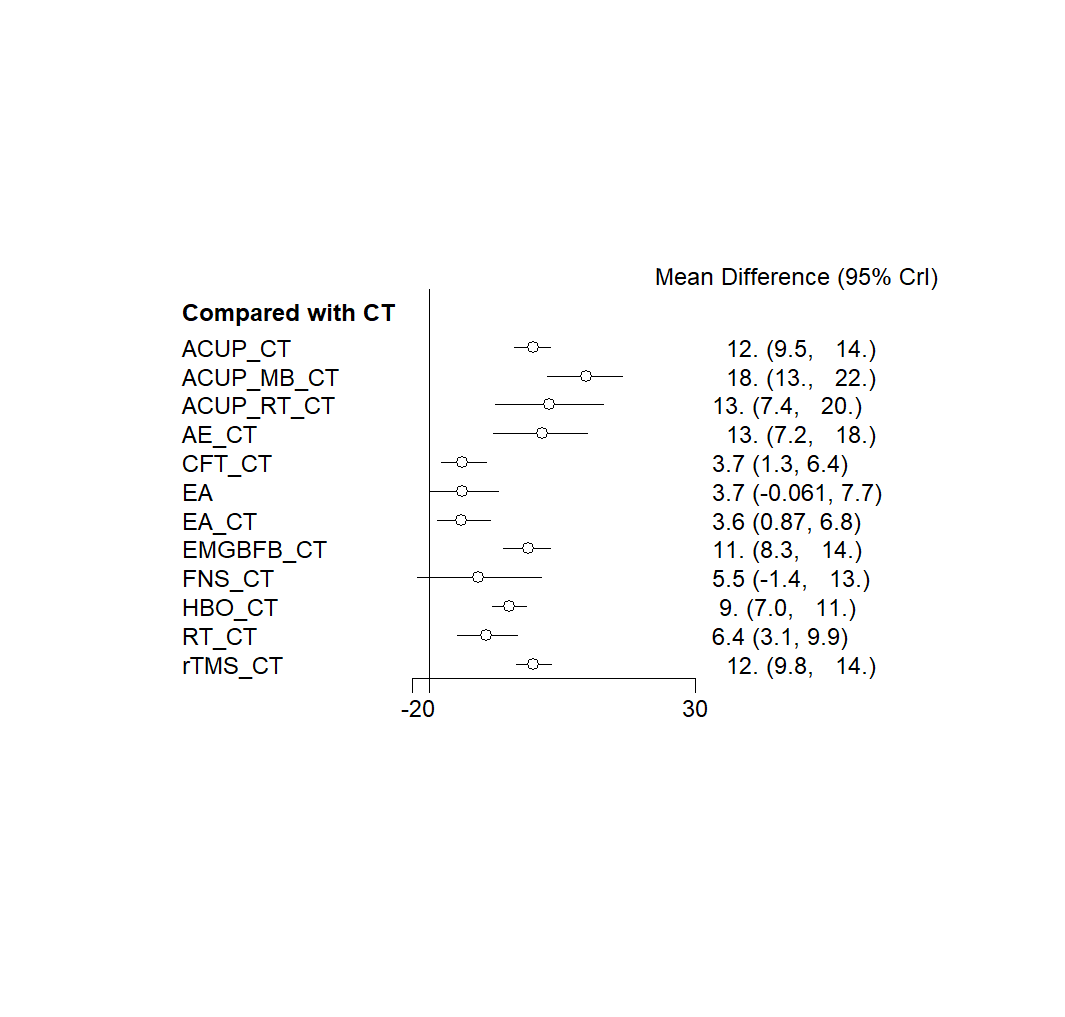


Figure 8.2.2: Forest plot adjusted for Sample Size. ACUP acupuncture, AE Aerobic exercise, CFT Cognitive function training, CT Conventional treatment, EMGBFB electromyographic biofeedback, EA electroacupunctur, FNS Fastigial nucleus stimulation, HBO hyperbaric oxygen therapy, MB moxibustion, RT Rehabilitation training, rTMS Repetitive Transcranial Magnetic Stimulation.

**8.2.3 Percentage Male**

When the model was adjusted for centering value of publish male 45, compared with the control group, the MD value of non-pharmacological interventions types did not change significantly, and the hierarchy from the unadjusted model largely retained.


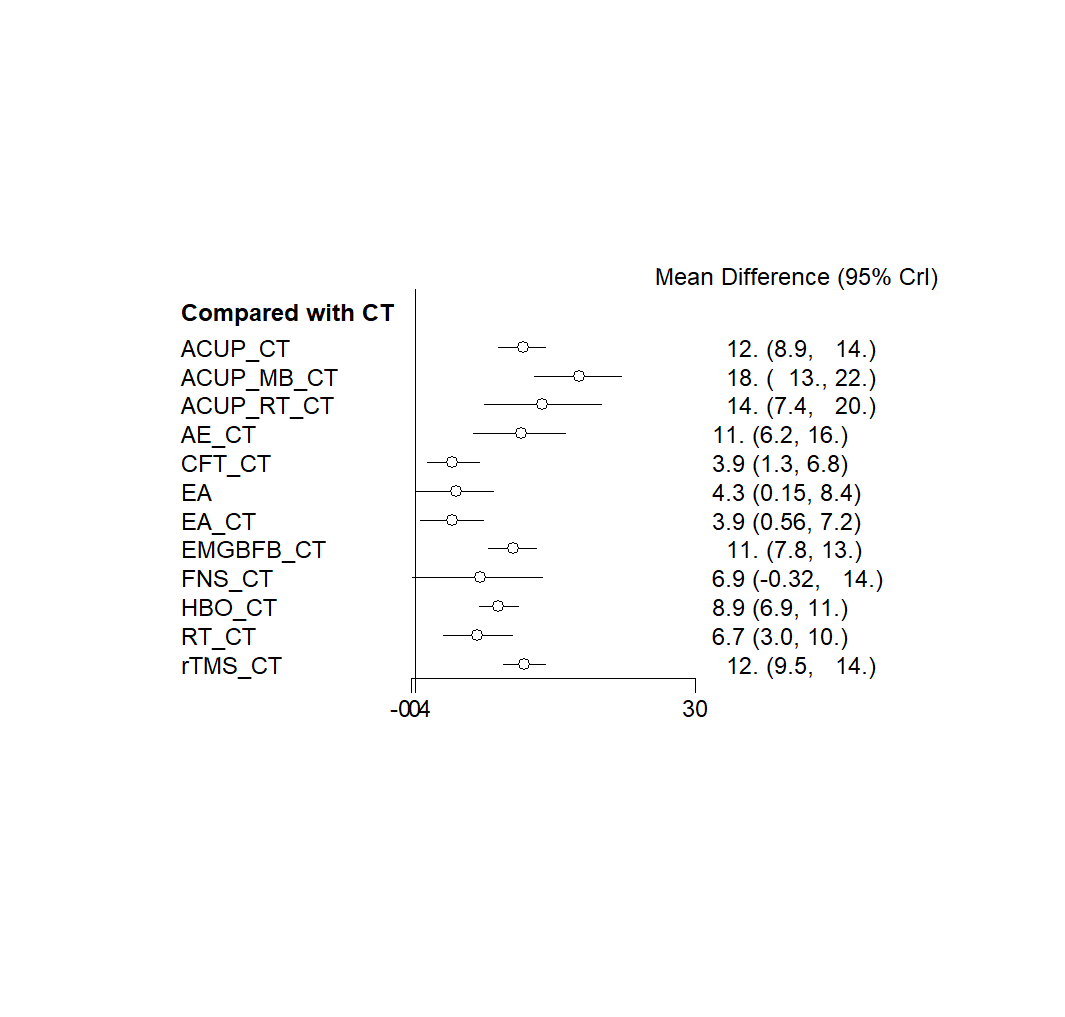


Figure 8.2.3: Forest plot adjusted for Percentage Male. ACUP acupuncture, AE Aerobic exercise, CFT Cognitive function training, CT Conventional treatment, EMGBFB electromyographic biofeedback, EA electroacupunctur, FNS Fastigial nucleus stimulation, HBO hyperbaric oxygen therapy, MB moxibustion, RT Rehabilitation training, rTMS Repetitive Transcranial Magnetic Stimulation.

**8.2.4** **Mean Age**

When the model was adjusted for centering value of publish age 68, compared with the control group, the MD value of non-pharmacological interventions types did not change significantly, and the hierarchy from the unadjusted model largely retained.


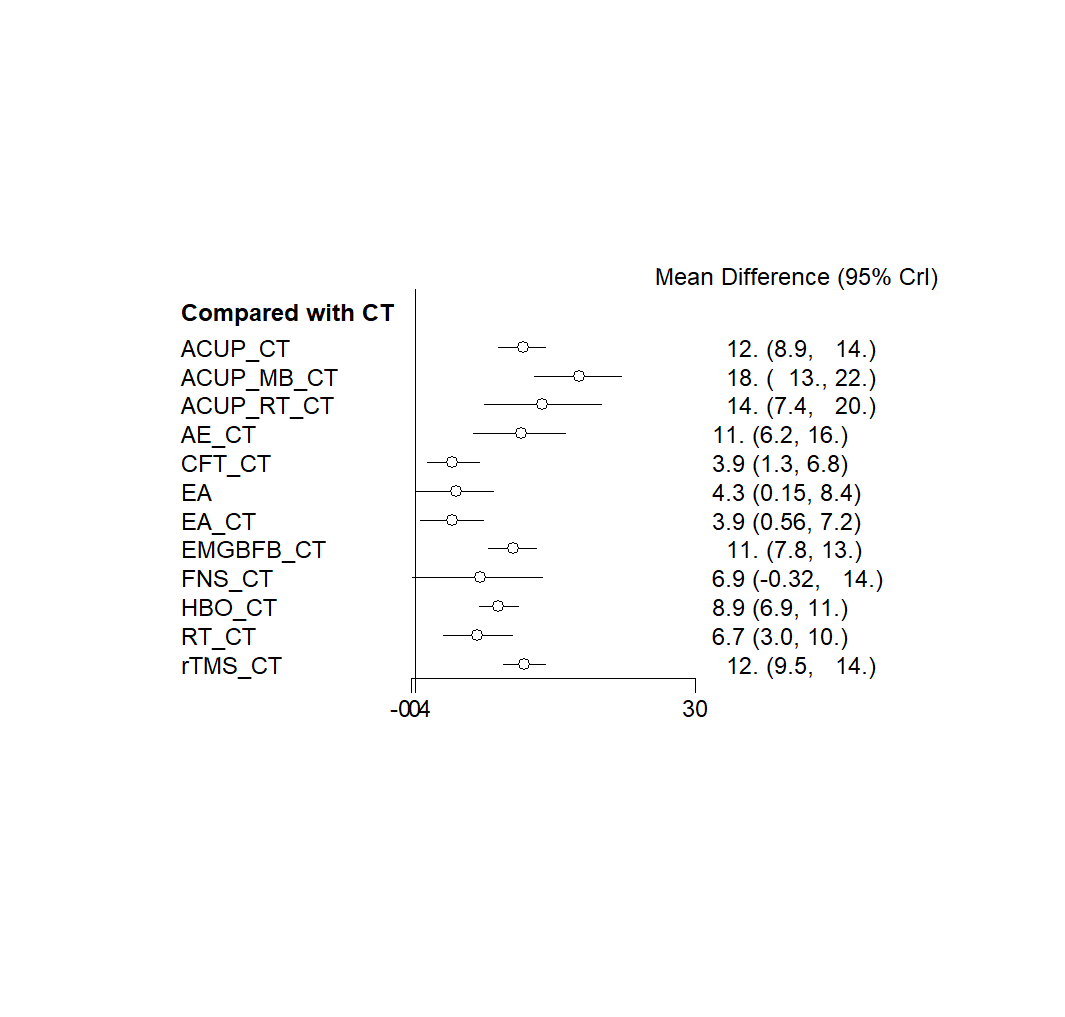


Figure 8.2.4: Forest plot adjusted for Mean Age. ACUP acupuncture, AE Aerobic exercise, CFT Cognitive function training, CT Conventional treatment, EMGBFB electromyographic biofeedback, EA electroacupunctur, FNS Fastigial nucleus stimulation, HBO hyperbaric oxygen therapy, MB moxibustion, RT Rehabilitation training, rTMS Repetitive Transcranial Magnetic Stimulation.

**8.2.5 Disease duration**

When the model was adjusted for centering value of publish duration 23, compared with the control group, the MD value of non-pharmacological interventions types did not change significantly, and the hierarchy from the unadjusted model largely retained.


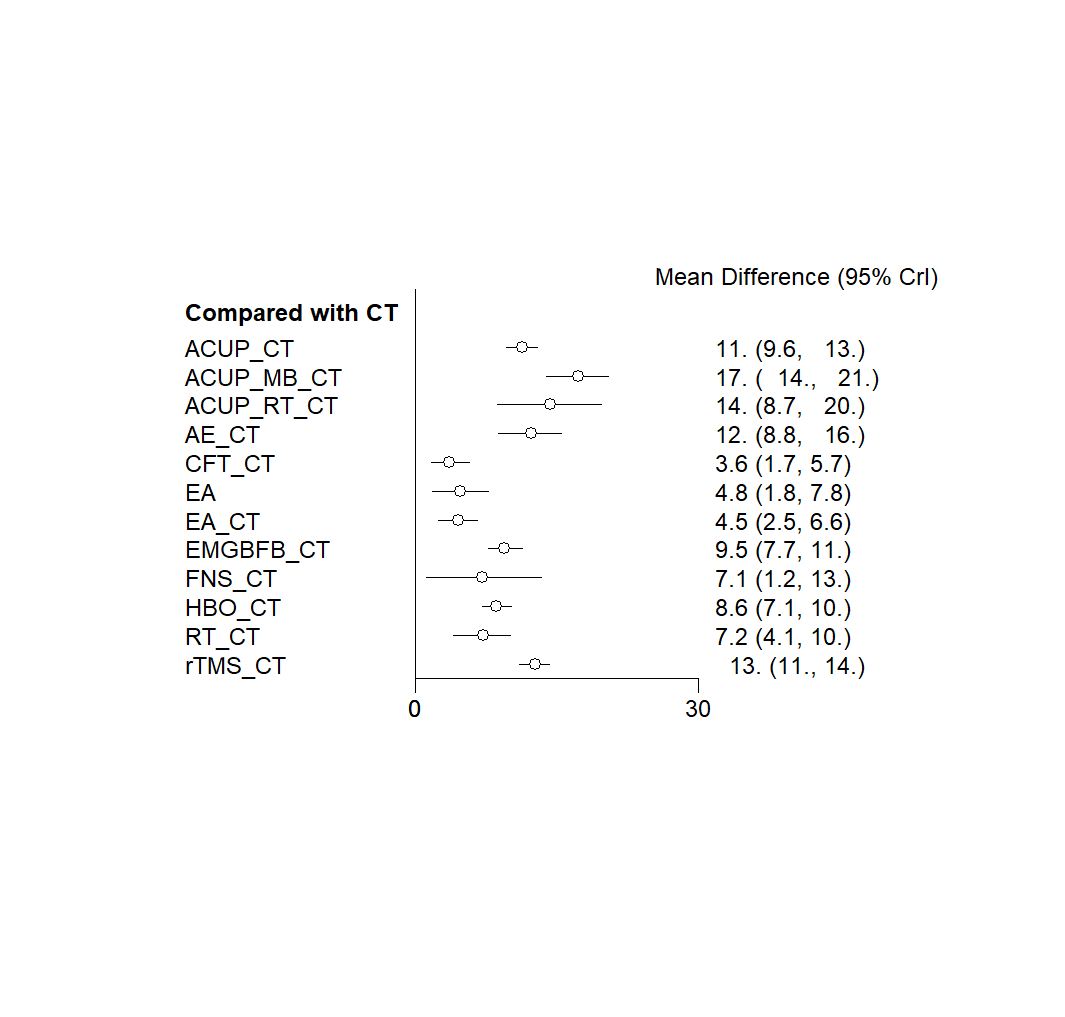


Figure 8.2.5: Forest plot adjusted for Disease duration. ACUP acupuncture, AE Aerobic exercise, CFT Cognitive function training, CT Conventional treatment, EMGBFB electromyographic biofeedback, EA electroacupunctur, FNS Fastigial nucleus stimulation, HBO hyperbaric oxygen therapy, MB moxibustion, RT Rehabilitation training, rTMS Repetitive Transcranial Magnetic Stimulation.

**8.2.6 Treatment period**

When the model was adjusted for centering value of publish period 7, compared with the control group, the MD value of non-pharmacological interventions types did not change significantly, and the hierarchy from the unadjusted model largely retained.


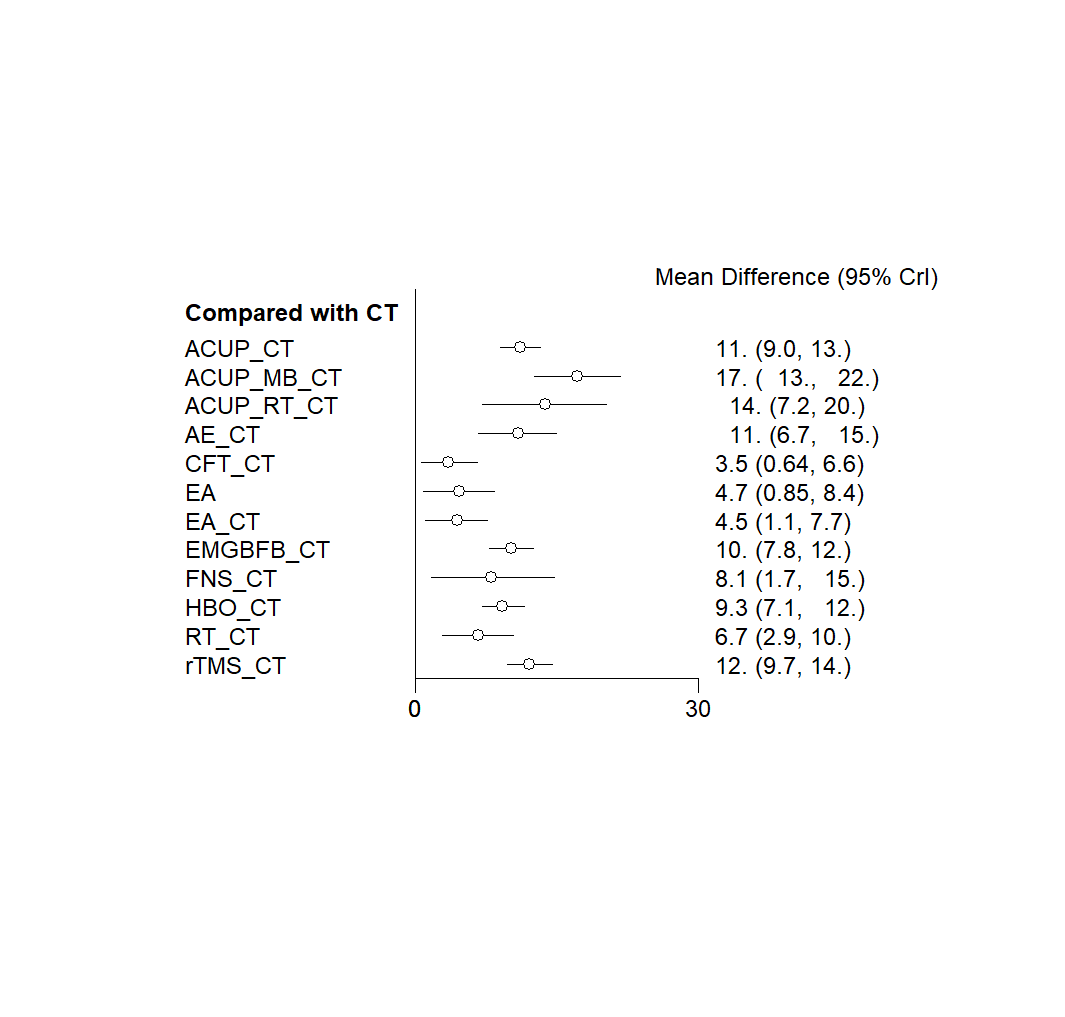


Figure 8.2.6: Forest plot adjusted for Treatment period. ACUP acupuncture, AE Aerobic exercise, CFT Cognitive function training, CT Conventional treatment, EMGBFB electromyographic biofeedback, EA electroacupunctur, FNS Fastigial nucleus stimulation, HBO hyperbaric oxygen therapy, MB moxibustion, RT Rehabilitation training, rTMS Repetitive Transcranial Magnetic Stimulation.

**8.2.7** **Treatment Frequency**

When the model was adjusted for centering value of publish frequency 7, compared with the control group, the MD value of non-pharmacological interventions types did not change significantly, and the hierarchy from the unadjusted model largely retained.


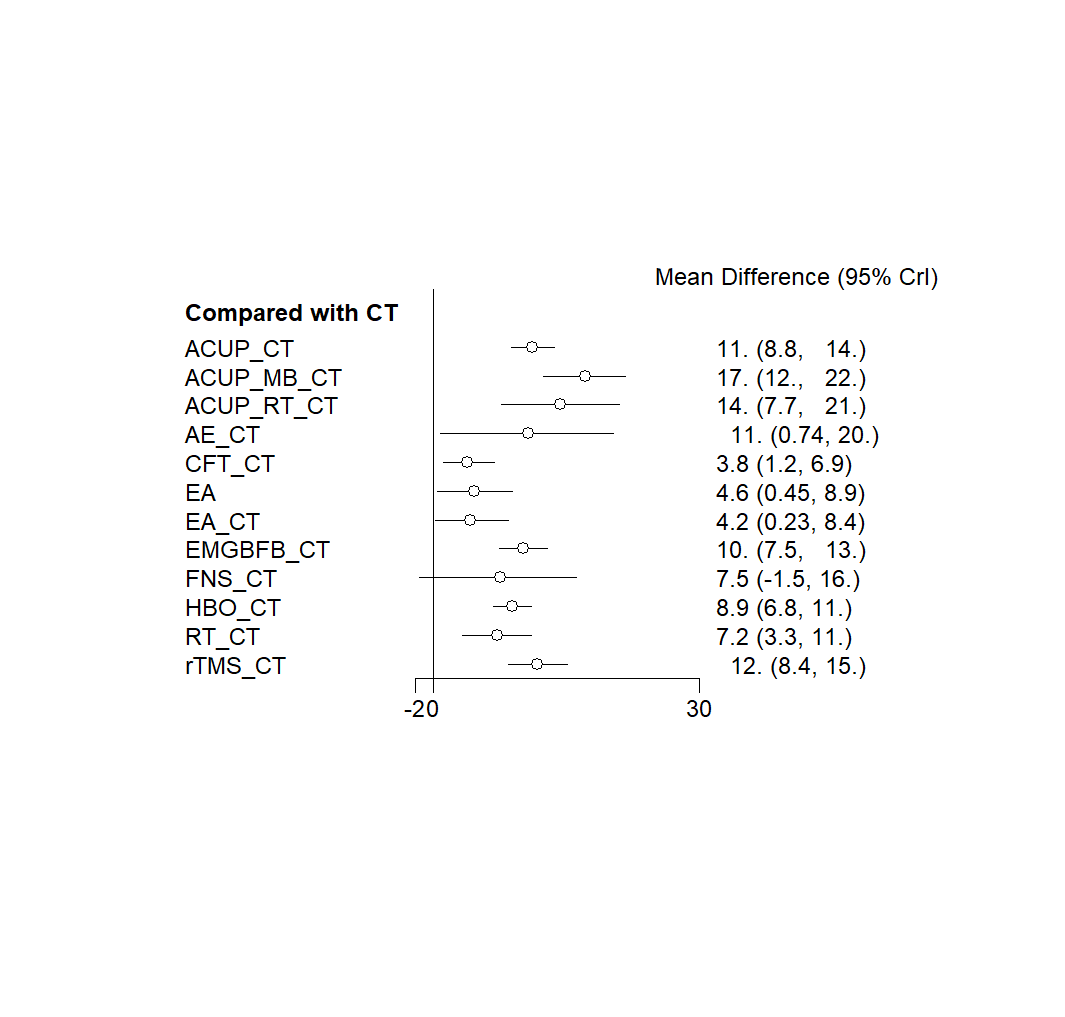


Figure 8.2.7: Forest plot adjusted for Treatment Frequency. ACUP acupuncture, AE Aerobic exercise, CFT Cognitive function training, CT Conventional treatment, EMGBFB electromyographic biofeedback, EA electroacupunctur, FNS Fastigial nucleus stimulation, HBO hyperbaric oxygen therapy, MB moxibustion, RT Rehabilitation training, rTMS Repetitive Transcranial Magnetic Stimulation.

**8.2.8 Treatment time**

When the model was adjusted for centering value of publish time 48 min, compared with the control group, the MD value of non-pharmacological interventions types did not change significantly, and the hierarchy from the unadjusted model largely retained.


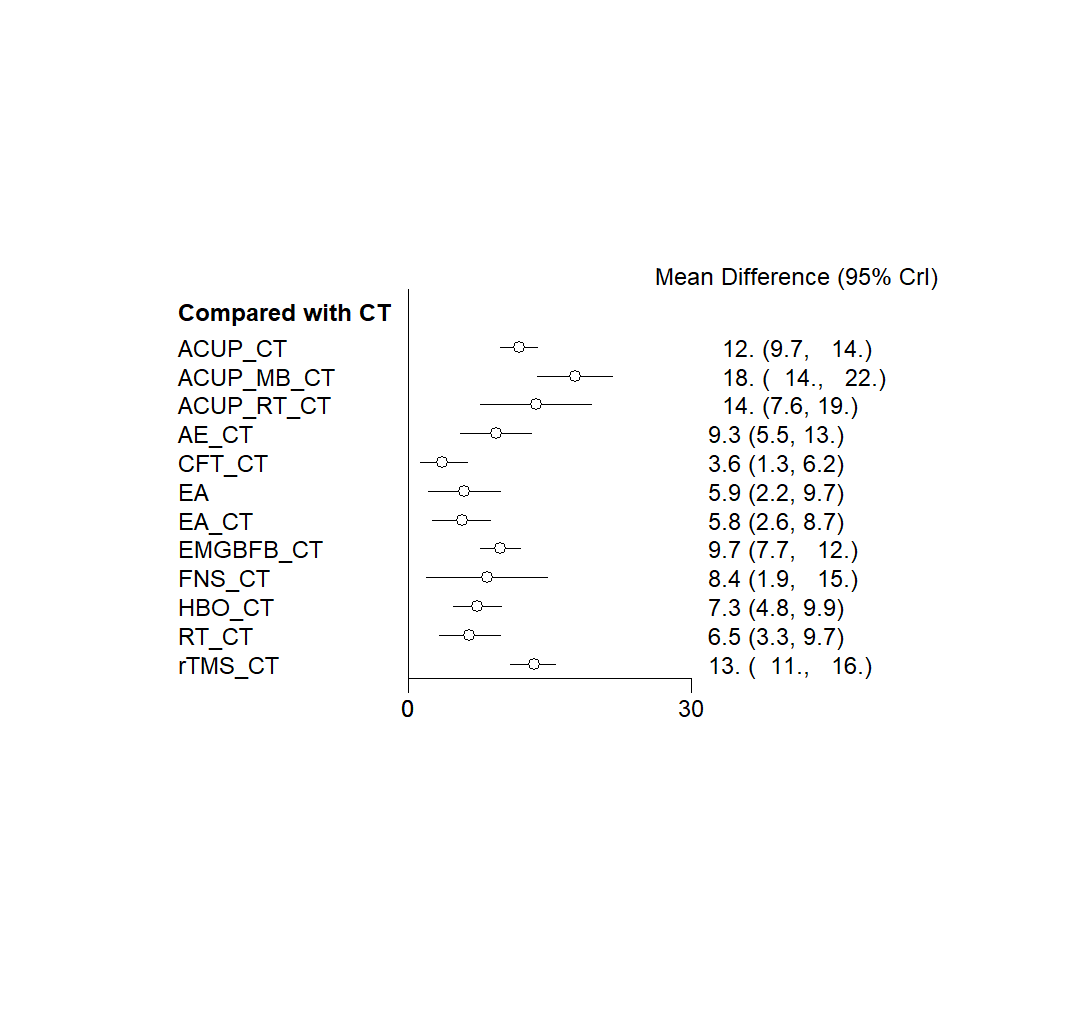


Figure 8.2.8: Forest plot adjusted for Treatment time. ACUP acupuncture, AE Aerobic exercise, CFT Cognitive function training, CT Conventional treatment, EMGBFB electromyographic biofeedback, EA electroacupunctur, FNS Fastigial nucleus stimulation, HBO hyperbaric oxygen therapy, MB moxibustion, RT Rehabilitation training, rTMS Repetitive Transcranial Magnetic Stimulation.

# Appendix 9：Sensitivity analyses

Below we present the results from the changes in heterogeneity in each sensitivity analysis

| **Including only studies with** | **Number of studies included** | **I^2^** | **Change** |
| --- | --- | --- | --- |
| **MMSE** | | | |
| None | 89 | 74.3% | - |
| treatment period 4-16 weeks | 78 | 76.5% | +2.2% |
| Exclude high-risk research. | 75 | 68.7% | -7.8% |
| **ADL** | | | |
| None | 27 | 54.8% | - |
| treatment period 4-16 weeks | 25 | 58.7% | +3.9% |
| Exclude high-risk research. | 22 | 54.8% | 0 |

## 9.1 treatment period 4-16 weeks

9.1.1 MMSE

When the model was adjusted for centering value of treatment period 4-16 weeks, compared with the control group, the MD value of non-pharmacological interventions types did not change significantly, and the hierarchy from the unadjusted model largely retained.


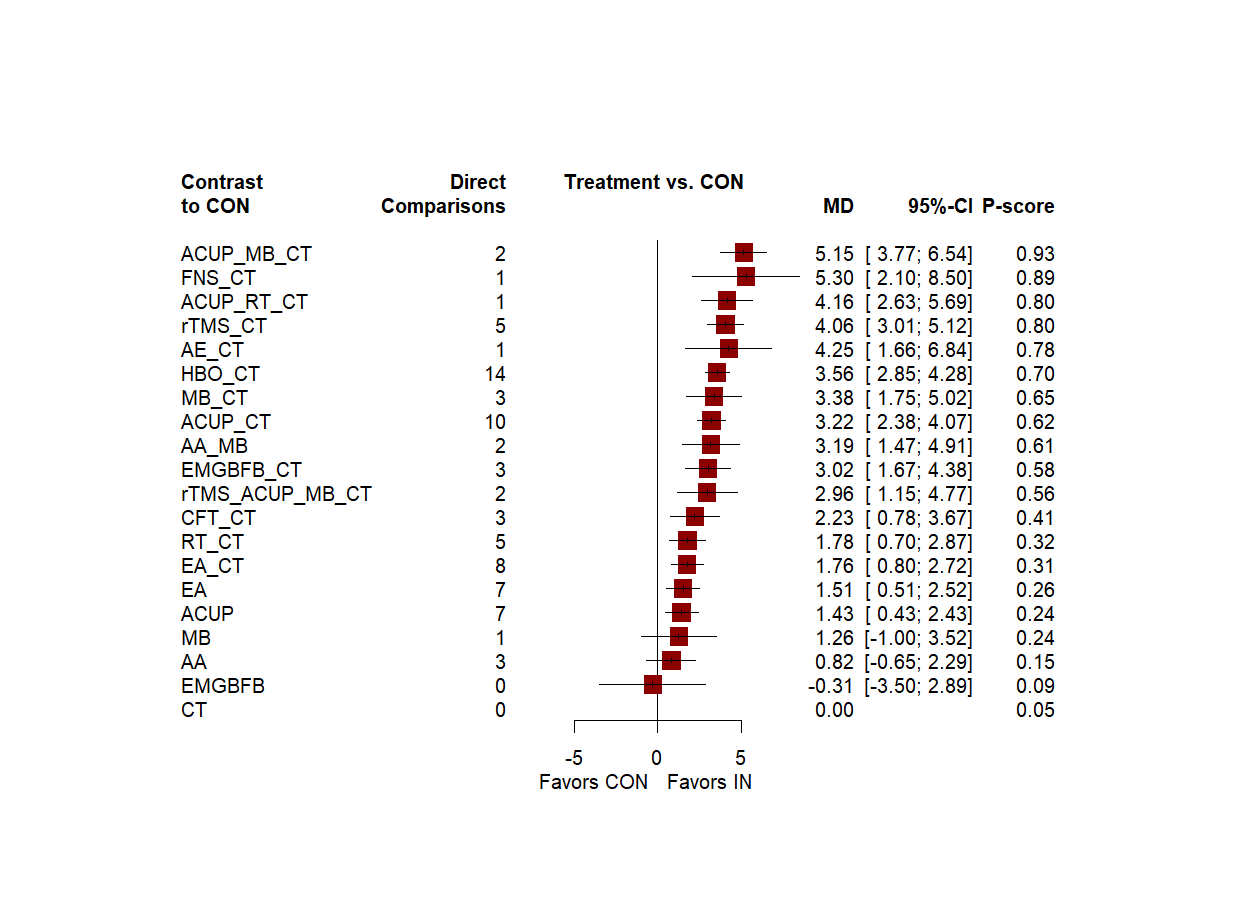


Figure 9.1.1: Forest plot after excluding studies based on the “treatment period 4-16 weeks” standard (MMSE). ACUP acupuncture, MB moxibustion, FNS Fastigial nucleus stimulation, RT Rehabilitation training, rTMS Repetitive Transcranial Magnetic Stimulation, AE Aerobic exercise, HBO hyperbaric oxygen therapy, AA auricular acupuncture, EMGBFB electromyographic biofeedback,CFT Cognitive function training, RT Rehabilitation training, EA electroacupunctur, CT Conventional treatment.

9.1.2 ADL

When the model was adjusted for centering value of treatment period 4-16 weeks, compared with the control group, the MD value of non-pharmacological interventions types did not change significantly, and the hierarchy from the unadjusted model largely retained.


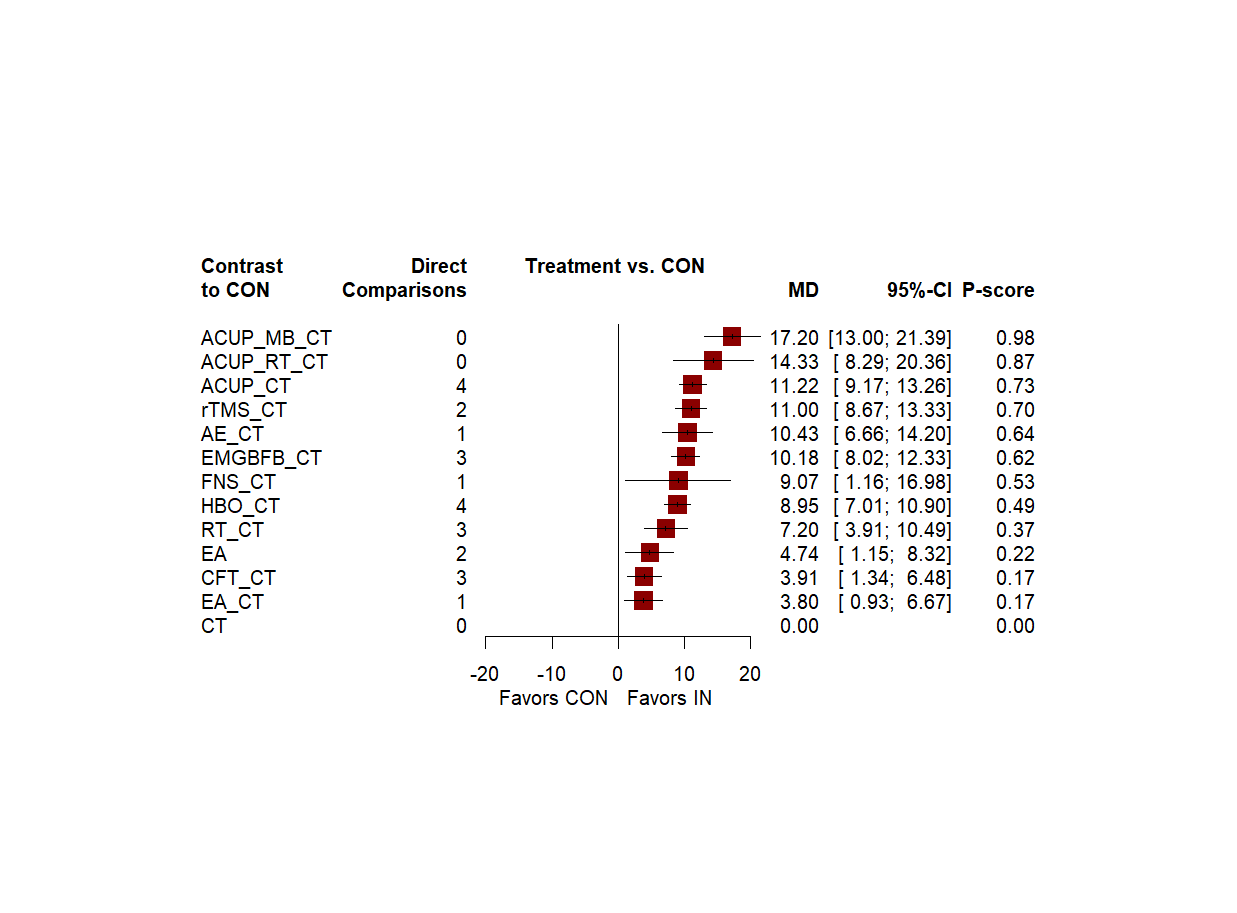


Figure 9.1.2: Forest plot after excluding studies based on the “treatment period 4-16 weeks” standard (ADL). ACUP acupuncture, MB moxibustion, FNS Fastigial nucleus stimulation, RT Rehabilitation training, rTMS Repetitive Transcranial Magnetic Stimulation, AE Aerobic exercise, HBO hyperbaric oxygen therapy, AA auricular acupuncture, EMGBFB electromyographic biofeedback,CFT Cognitive function training, RT Rehabilitation training, EA electroacupunctur, CT Conventional treatment.

## 9.2 Exclude high-risk research.

9.2.1 MMSE

When the model was adjusted for centering value of high-risk research, compared with the control group, the MD value of non-pharmacological interventions types did not change significantly, and the hierarchy from the unadjusted model largely retained.


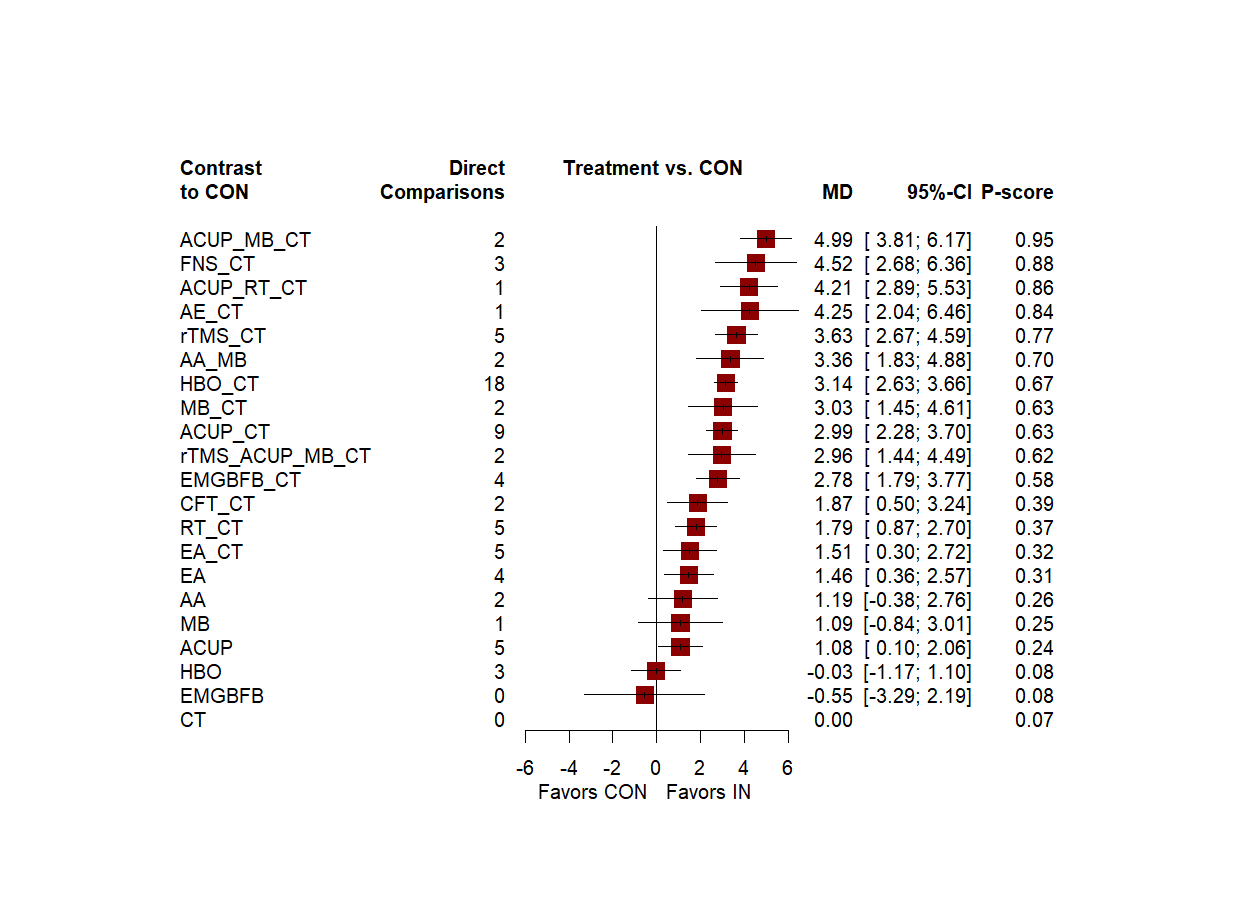


Figure 9.2.1: Forest plot after excluding studies based on the “Exclude high-risk research” standard (MMSE). ACUP acupuncture, MB moxibustion, FNS Fastigial nucleus stimulation, RT Rehabilitation training, rTMS Repetitive Transcranial Magnetic Stimulation, AE Aerobic exercise, HBO hyperbaric oxygen therapy, AA auricular acupuncture, EMGBFB electromyographic biofeedback,CFT Cognitive function training, RT Rehabilitation training, EA electroacupunctur, CT Conventional treatment.

9.2.2 ADL

When the model was adjusted for centering value of high-risk research, compared with the control group, the MD value of non-pharmacological interventions types did not change significantly, and the hierarchy from the unadjusted model largely retained.


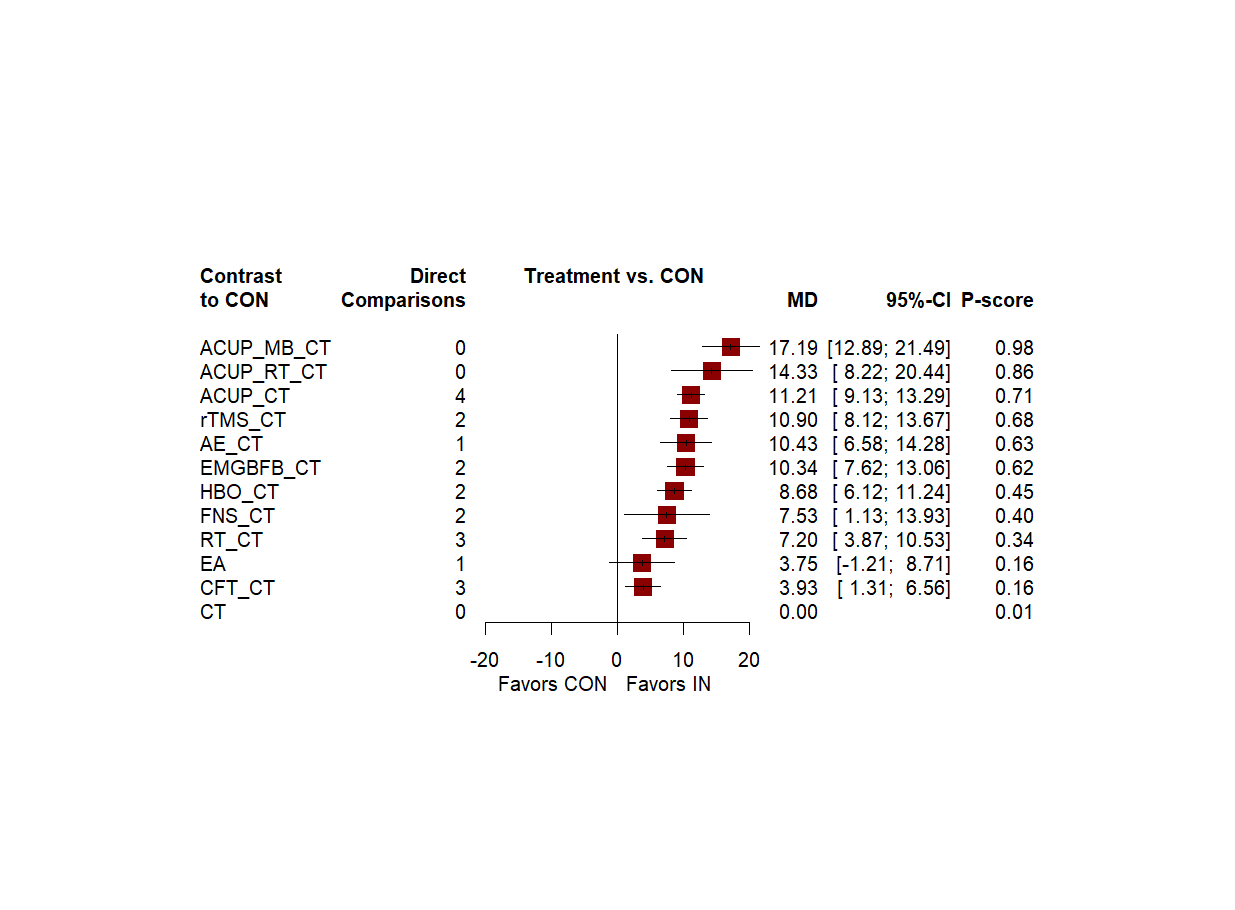


Figure 9.2.2: Forest plot after excluding studies based on the “Exclude high-risk research” standard (ADL). ACUP acupuncture, MB moxibustion, FNS Fastigial nucleus stimulation, RT Rehabilitation training, rTMS Repetitive Transcranial Magnetic Stimulation, AE Aerobic exercise, HBO hyperbaric oxygen therapy, AA auricular acupuncture, EMGBFB electromyographic biofeedback,CFT Cognitive function training, RT Rehabilitation training, EA electroacupunctur, CT Conventional treatment.

# Appendix 10: Grading the evidence for outcomes of the network meta-analysis using CINeMA

## 10.1 MMSE

10.1.1 Summary of study limitations of the included studies

The figure below showed that rTMS_CT, EA_CT, AUCP_CT, AUCP, EA, MB_CT, CFT_CT, HBO_CT and AA had high risk of bias (RoB) studies.


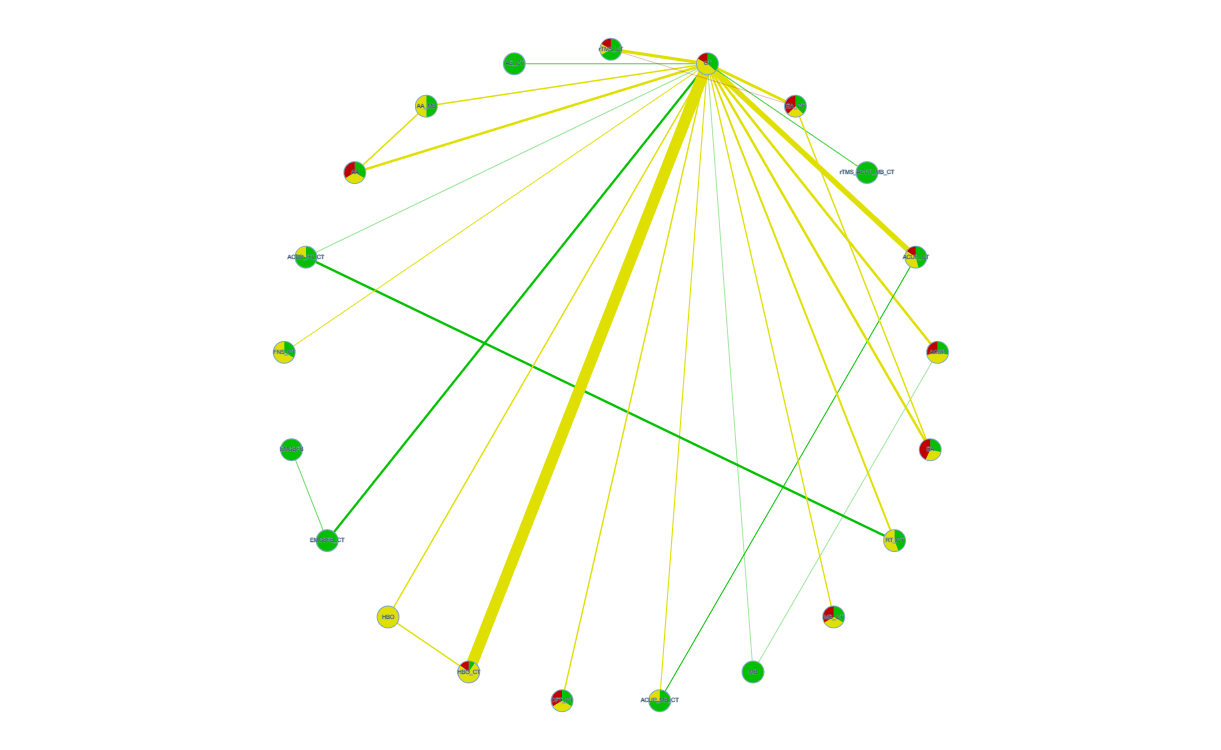


Figure 1 Network plot of study limitations of the included studies (MMSE). Node size by equal size, node color by RoB. The colors in the circles indicate the percentage of low RoB studies (green), moderate RoB studies (yellow), high RoB studies (red) about each physical activity type. Edge width by sample size. Edge color by average RoB. The colors of the lines indicate the summative RoB assessment of each comparison. Low RoB is green, moderate RoB is yellow, high RoB is red.

10.1.2 Contribution percentage of low, moderate, and high RoB comparisons to each network estimate.

Low RoB is green, moderate RoB is yellow, high RoB is red.


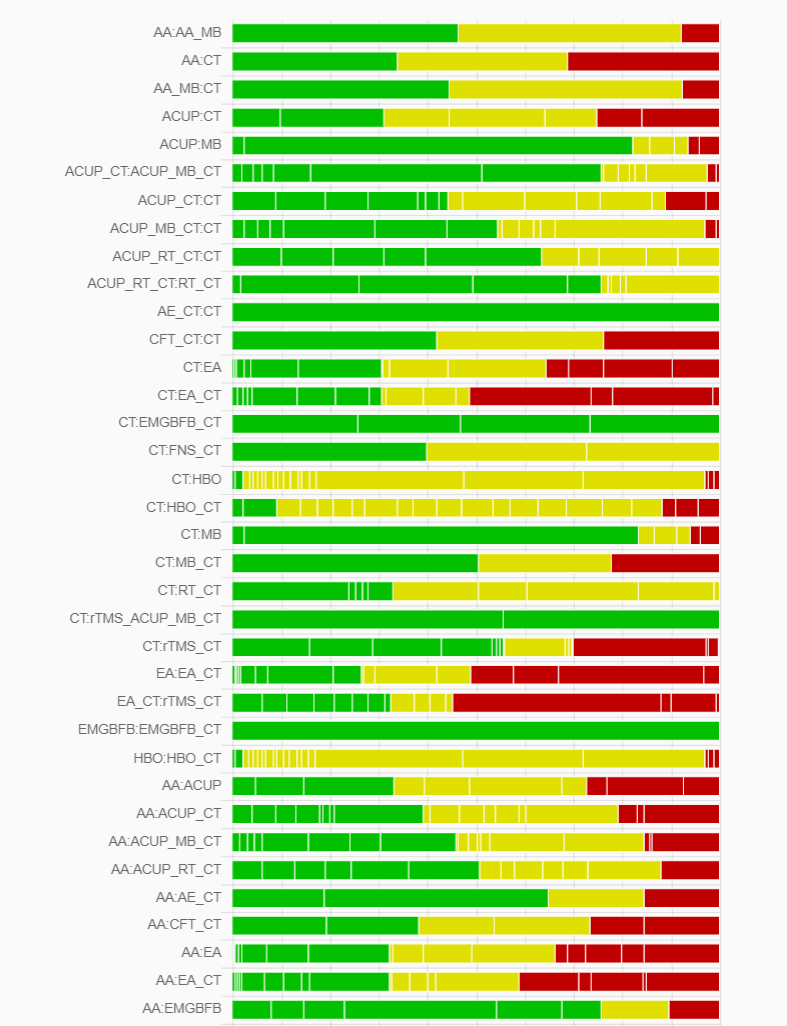


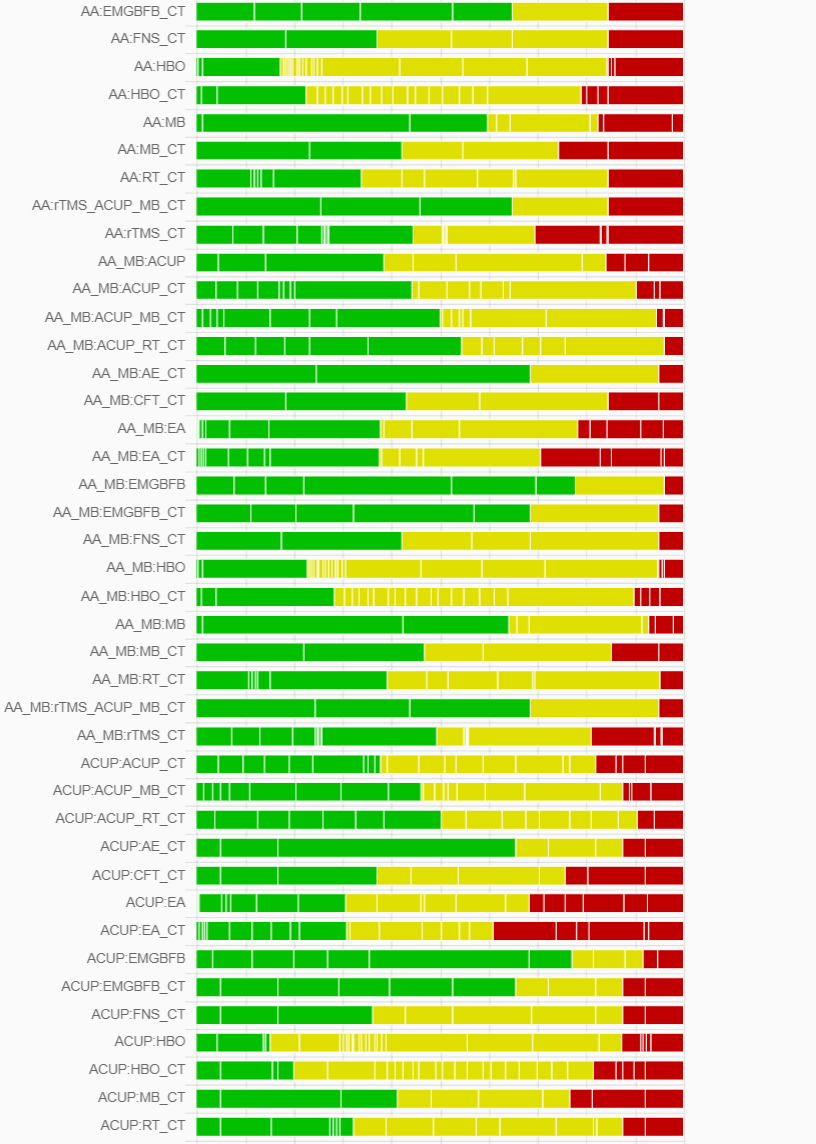


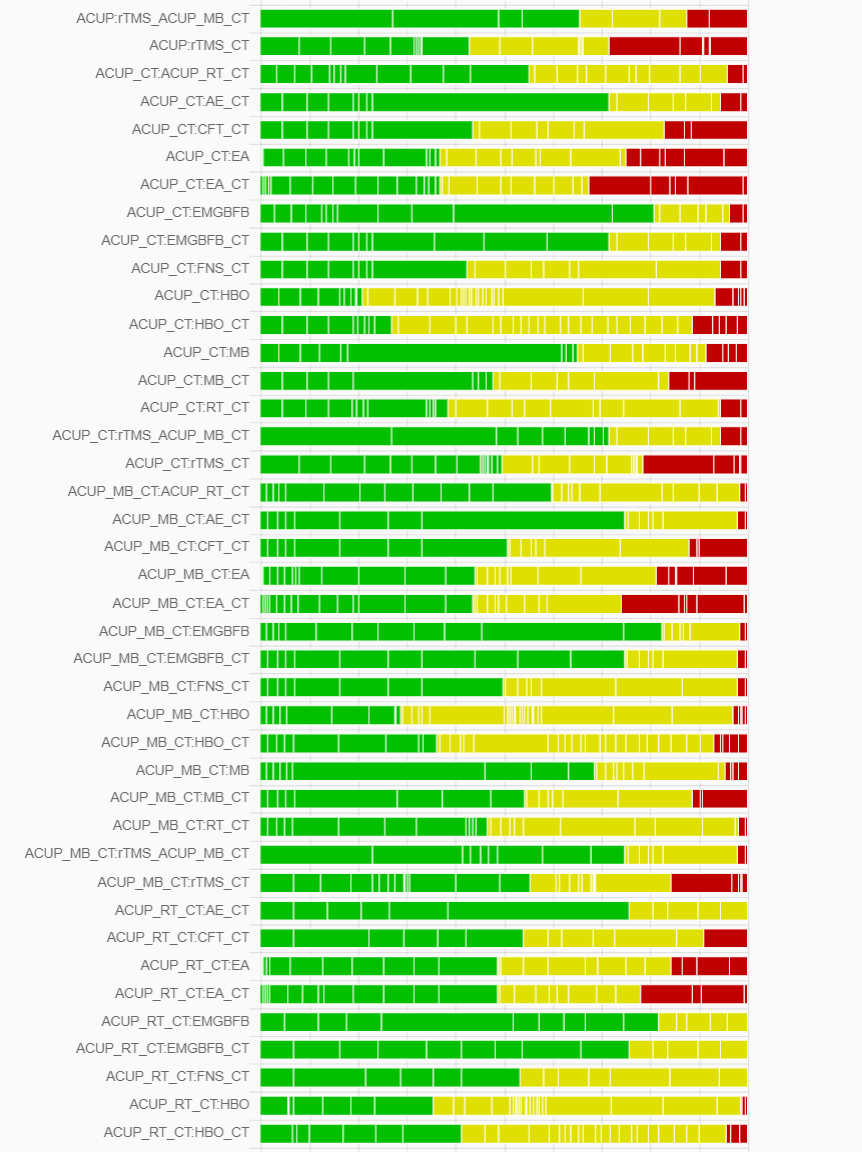


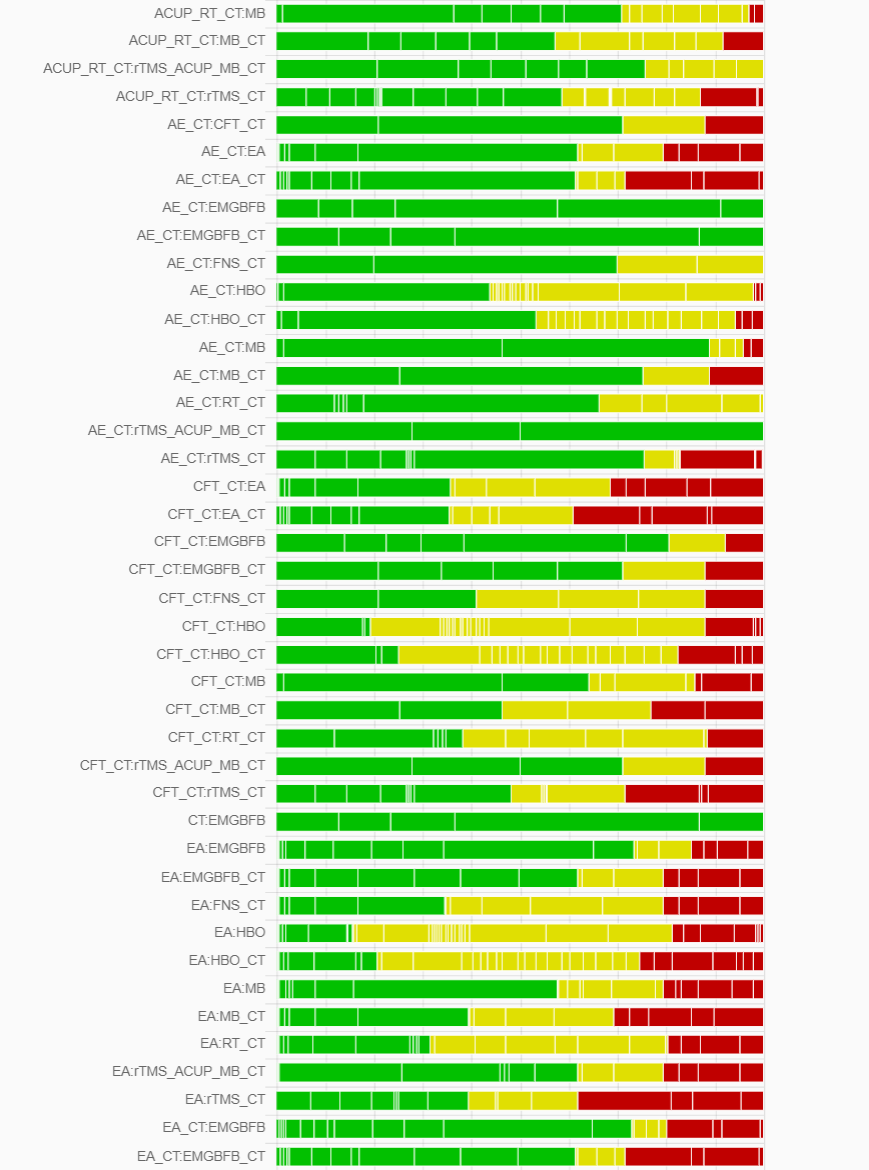

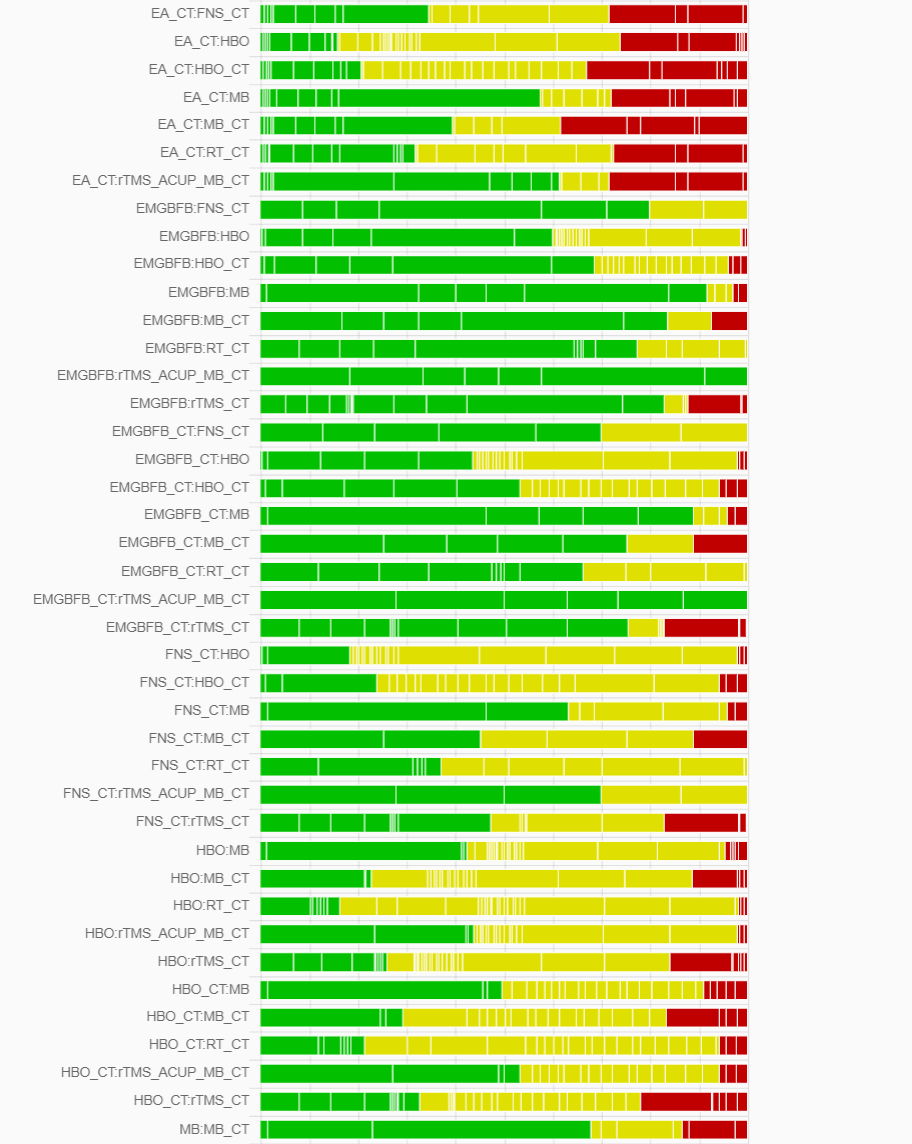

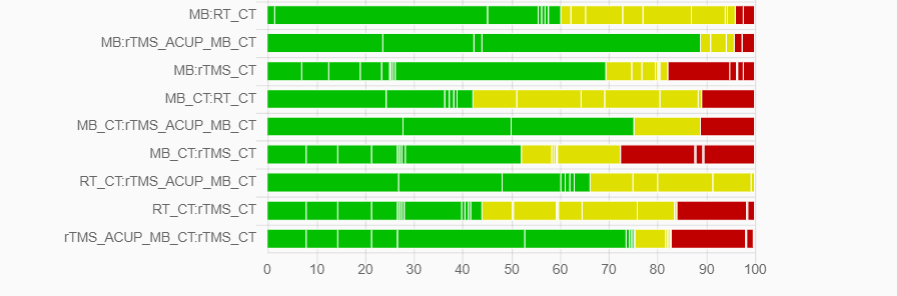


10.1.3 CINeMA for the primary outcome “MMSE”

| **Comparison** | **Number of studies** | **Within-study bias** | **Reporting bias** | **Indirectness** | **Imprecision** | **Heterogeneity** | **Incoherence** | **Confidence rating** |
| --- | --- | --- | --- | --- | --- | --- | --- | --- |
| AA:AA_MB | 2 | Some concerns | Low risk | No concerns | No concerns | Major concerns | No concerns | Very low |
| AA:CT | 3 | Some concerns | Low risk | No concerns | Major concerns | No concerns | Some concerns | Very low |
| AA_MB:CT | 2 | Some concerns | Low risk | No concerns | No concerns | No concerns | No concerns | Moderate |
| ACUP:CT | 7 | Some concerns | Low risk | No concerns | No concerns | Major concerns | Some concerns | Very low |
| ACUP:MB | 1 | No concerns | Low risk | No concerns | Major concerns | No concerns | No concerns | Low |
| ACUP_CT:ACUP_MB_CT | 2 | No concerns | Low risk | No concerns | No concerns | Major concerns | No concerns | Low |
| ACUP_CT:CT | 11 | Some concerns | Low risk | No concerns | No concerns | No concerns | No concerns | Moderate |
| ACUP_MB_CT:CT | 2 | No concerns | Low risk | No concerns | No concerns | No concerns | No concerns | High |
| ACUP_RT_CT:CT | 1 | No concerns | Low risk | No concerns | No concerns | No concerns | No concerns | High |
| ACUP_RT_CT:RT_CT | 4 | No concerns | Low risk | No concerns | No concerns | No concerns | No concerns | High |
| AE_CT:CT | 1 | No concerns | Low risk | No concerns | No concerns | No concerns | Some concerns | Moderate |
| CFT_CT:CT | 3 | Some concerns | Low risk | No concerns | No concerns | Major concerns | Some concerns | Very low |
| CT:EA | 7 | Some concerns | Low risk | No concerns | No concerns | Major concerns | No concerns | Very low |
| CT:EA_CT | 8 | Some concerns | Low risk | No concerns | No concerns | Major concerns | No concerns | Very low |
| CT:EMGBFB_CT | 4 | No concerns | Low risk | No concerns | No concerns | No concerns | Some concerns | Moderate |
| CT:FNS_CT | 3 | Some concerns | Low risk | No concerns | No concerns | No concerns | Some concerns | Low |
| CT:HBO | 3 | Some concerns | Low risk | No concerns | Major concerns | No concerns | No concerns | Very low |
| CT:HBO_CT | 21 | Some concerns | Low risk | No concerns | No concerns | No concerns | Some concerns | Low |
| CT:MB | 1 | No concerns | Low risk | No concerns | Major concerns | No concerns | No concerns | Low |
| CT:MB_CT | 3 | Some concerns | Low risk | No concerns | No concerns | No concerns | Some concerns | Low |
| CT:RT_CT | 5 | Some concerns | Low risk | No concerns | No concerns | Major concerns | No concerns | Very low |
| CT:rTMS_ACUP_MB_CT | 2 | No concerns | Low risk | No concerns | No concerns | No concerns | Some concerns | Moderate |
| CT:rTMS_CT | 6 | Some concerns | Low risk | No concerns | No concerns | No concerns | No concerns | Moderate |
| EA:EA_CT | 4 | Some concerns | Low risk | No concerns | Major concerns | No concerns | No concerns | Very low |
| EA_CT:rTMS_CT | 1 | Some concerns | Low risk | No concerns | No concerns | Major concerns | No concerns | Very low |
| EMGBFB:EMGBFB_CT | 1 | No concerns | Low risk | No concerns | No concerns | Major concerns | Some concerns | Low |
| HBO:HBO_CT | 3 | Some concerns | Low risk | No concerns | No concerns | No concerns | No concerns | Moderate |
| AA:ACUP | 0 | Some concerns | Low risk | No concerns | Major concerns | No concerns | Some concerns | Very low |
| AA:ACUP_CT | 0 | Some concerns | Low risk | No concerns | No concerns | Major concerns | Some concerns | Very low |
| AA:ACUP_MB_CT | 0 | Some concerns | Low risk | No concerns | No concerns | No concerns | Some concerns | Low |
| AA:ACUP_RT_CT | 0 | Some concerns | Low risk | No concerns | No concerns | No concerns | Some concerns | Low |
| AA:AE_CT | 0 | Some concerns | Low risk | No concerns | No concerns | No concerns | Some concerns | Low |
| AA:CFT_CT | 0 | Some concerns | Low risk | No concerns | Major concerns | No concerns | Some concerns | Very low |
| AA:EA | 0 | Some concerns | Low risk | No concerns | Major concerns | No concerns | Some concerns | Very low |
| AA:EA_CT | 0 | Some concerns | Low risk | No concerns | Major concerns | No concerns | Some concerns | Very low |
| AA:EMGBFB | 0 | No concerns | Low risk | No concerns | Major concerns | No concerns | Some concerns | Low |
| AA:EMGBFB_CT | 0 | Some concerns | Low risk | No concerns | No concerns | Major concerns | Some concerns | Very low |
| AA:FNS_CT | 0 | Some concerns | Low risk | No concerns | No concerns | No concerns | Some concerns | Low |
| AA:HBO | 0 | Some concerns | Low risk | No concerns | Major concerns | No concerns | Some concerns | Very low |
| AA:HBO_CT | 0 | Some concerns | Low risk | No concerns | No concerns | Major concerns | Some concerns | Very low |
| AA:MB | 0 | Some concerns | Low risk | No concerns | Major concerns | No concerns | Some concerns | Very low |
| AA:MB_CT | 0 | Some concerns | Low risk | No concerns | No concerns | Major concerns | Some concerns | Very low |
| AA:RT_CT | 0 | Some concerns | Low risk | No concerns | Major concerns | No concerns | Some concerns | Very low |
| AA:rTMS_ACUP_MB_CT | 0 | Some concerns | Low risk | No concerns | No concerns | Major concerns | Some concerns | Very low |
| AA:rTMS_CT | 0 | Some concerns | Low risk | No concerns | No concerns | No concerns | Some concerns | Low |
| AA_MB:ACUP | 0 | Some concerns | Low risk | No concerns | Major concerns | No concerns | Some concerns | Very low |
| AA_MB:ACUP_CT | 0 | Some concerns | Low risk | No concerns | Major concerns | No concerns | Some concerns | Very low |
| AA_MB:ACUP_MB_CT | 0 | Some concerns | Low risk | No concerns | Major concerns | No concerns | Some concerns | Very low |
| AA_MB:ACUP_RT_CT | 0 | No concerns | Low risk | No concerns | Major concerns | No concerns | Some concerns | Low |
| AA_MB:AE_CT | 0 | No concerns | Low risk | No concerns | Major concerns | No concerns | Some concerns | Low |
| AA_MB:CFT_CT | 0 | Some concerns | Low risk | No concerns | Major concerns | No concerns | Some concerns | Very low |
| AA_MB:EA | 0 | Some concerns | Low risk | No concerns | Major concerns | No concerns | Some concerns | Very low |
| AA_MB:EA_CT | 0 | Some concerns | Low risk | No concerns | Major concerns | No concerns | Some concerns | Very low |
| AA_MB:EMGBFB | 0 | No concerns | Low risk | No concerns | No concerns | Major concerns | Some concerns | Low |
| AA_MB:EMGBFB_CT | 0 | No concerns | Low risk | No concerns | Major concerns | No concerns | Some concerns | Low |
| AA_MB:FNS_CT | 0 | Some concerns | Low risk | No concerns | Major concerns | No concerns | Some concerns | Very low |
| AA_MB:HBO | 0 | Some concerns | Low risk | No concerns | No concerns | No concerns | Some concerns | Low |
| AA_MB:HBO_CT | 0 | Some concerns | Low risk | No concerns | Major concerns | No concerns | Some concerns | Very low |
| AA_MB:MB | 0 | No concerns | Low risk | No concerns | Major concerns | No concerns | Some concerns | Low |
| AA_MB:MB_CT | 0 | Some concerns | Low risk | No concerns | Major concerns | No concerns | Some concerns | Very low |
| AA_MB:RT_CT | 0 | Some concerns | Low risk | No concerns | Major concerns | No concerns | Some concerns | Very low |
| AA_MB:rTMS_ACUP_MB_CT | 0 | No concerns | Low risk | No concerns | Major concerns | No concerns | Some concerns | Low |
| AA_MB:rTMS_CT | 0 | Some concerns | Low risk | No concerns | Major concerns | No concerns | Some concerns | Very low |
| ACUP:ACUP_CT | 0 | Some concerns | Low risk | No concerns | No concerns | Major concerns | Some concerns | Very low |
| ACUP:ACUP_MB_CT | 0 | Some concerns | Low risk | No concerns | No concerns | No concerns | Some concerns | Low |
| ACUP:ACUP_RT_CT | 0 | Some concerns | Low risk | No concerns | No concerns | No concerns | Some concerns | Low |
| ACUP:AE_CT | 0 | No concerns | Low risk | No concerns | No concerns | Major concerns | Some concerns | Low |
| ACUP:CFT_CT | 0 | Some concerns | Low risk | No concerns | Major concerns | No concerns | Some concerns | Very low |
| ACUP:EA | 0 | Some concerns | Low risk | No concerns | Major concerns | No concerns | Some concerns | Very low |
| ACUP:EA_CT | 0 | Some concerns | Low risk | No concerns | Major concerns | No concerns | Some concerns | Very low |
| ACUP:EMGBFB | 0 | No concerns | Low risk | No concerns | Major concerns | No concerns | Some concerns | Low |
| ACUP:EMGBFB_CT | 0 | No concerns | Low risk | No concerns | Major concerns | No concerns | Some concerns | Low |
| ACUP:FNS_CT | 0 | Some concerns | Low risk | No concerns | No concerns | No concerns | Some concerns | Low |
| ACUP:HBO | 0 | Some concerns | Low risk | No concerns | Major concerns | No concerns | Some concerns | Very low |
| ACUP:HBO_CT | 0 | Some concerns | Low risk | No concerns | No concerns | Major concerns | Some concerns | Very low |
| ACUP:MB_CT | 0 | Some concerns | Low risk | No concerns | No concerns | Major concerns | Some concerns | Very low |
| ACUP:RT_CT | 0 | Some concerns | Low risk | No concerns | Major concerns | No concerns | Some concerns | Very low |
| ACUP:rTMS_ACUP_MB_CT | 0 | No concerns | Low risk | No concerns | Major concerns | No concerns | Some concerns | Low |
| ACUP:rTMS_CT | 0 | Some concerns | Low risk | No concerns | No concerns | No concerns | Some concerns | Low |
| ACUP_CT:ACUP_RT_CT | 0 | No concerns | Low risk | No concerns | Major concerns | No concerns | Some concerns | Low |
| ACUP_CT:AE_CT | 0 | No concerns | Low risk | No concerns | Major concerns | No concerns | Some concerns | Low |
| ACUP_CT:CFT_CT | 0 | Some concerns | Low risk | No concerns | Major concerns | No concerns | Some concerns | Very low |
| ACUP_CT:EA | 0 | Some concerns | Low risk | No concerns | No concerns | Major concerns | Some concerns | Very low |
| ACUP_CT:EA_CT | 0 | Some concerns | Low risk | No concerns | No concerns | Major concerns | Some concerns | Very low |
| ACUP_CT:EMGBFB | 0 | No concerns | Low risk | No concerns | No concerns | No concerns | Some concerns | Moderate |
| ACUP_CT:EMGBFB_CT | 0 | No concerns | Low risk | No concerns | Major concerns | No concerns | Some concerns | Low |
| ACUP_CT:FNS_CT | 0 | Some concerns | Low risk | No concerns | Major concerns | No concerns | Some concerns | Very low |
| ACUP_CT:HBO | 0 | Some concerns | Low risk | No concerns | No concerns | No concerns | Some concerns | Low |
| ACUP_CT:HBO_CT | 0 | Some concerns | Low risk | No concerns | Major concerns | No concerns | Some concerns | Very low |
| ACUP_CT:MB | 0 | No concerns | Low risk | No concerns | Major concerns | No concerns | Some concerns | Low |
| ACUP_CT:MB_CT | 0 | Some concerns | Low risk | No concerns | Major concerns | No concerns | Some concerns | Very low |
| ACUP_CT:RT_CT | 0 | Some concerns | Low risk | No concerns | No concerns | Major concerns | Some concerns | Very low |
| ACUP_CT:rTMS_ACUP_MB_CT | 0 | No concerns | Low risk | No concerns | Major concerns | No concerns | Some concerns | Low |
| ACUP_CT:rTMS_CT | 0 | Some concerns | Low risk | No concerns | Major concerns | No concerns | Some concerns | Very low |
| ACUP_MB_CT:ACUP_RT_CT | 0 | No concerns | Low risk | No concerns | Major concerns | No concerns | Some concerns | Low |
| ACUP_MB_CT:AE_CT | 0 | No concerns | Low risk | No concerns | Major concerns | No concerns | Some concerns | Low |
| ACUP_MB_CT:CFT_CT | 0 | Some concerns | Low risk | No concerns | No concerns | No concerns | Some concerns | Low |
| ACUP_MB_CT:EA | 0 | Some concerns | Low risk | No concerns | No concerns | No concerns | Some concerns | Low |
| ACUP_MB_CT:EA_CT | 0 | Some concerns | Low risk | No concerns | No concerns | No concerns | Some concerns | Low |
| ACUP_MB_CT:EMGBFB | 0 | No concerns | Low risk | No concerns | No concerns | No concerns | Some concerns | Moderate |
| ACUP_MB_CT:EMGBFB_CT | 0 | No concerns | Low risk | No concerns | No concerns | Major concerns | Some concerns | Low |
| ACUP_MB_CT:FNS_CT | 0 | Some concerns | Low risk | No concerns | Major concerns | No concerns | Some concerns | Very low |
| ACUP_MB_CT:HBO | 0 | Some concerns | Low risk | No concerns | No concerns | No concerns | Some concerns | Low |
| ACUP_MB_CT:HBO_CT | 0 | Some concerns | Low risk | No concerns | No concerns | Major concerns | Some concerns | Very low |
| ACUP_MB_CT:MB | 0 | No concerns | Low risk | No concerns | No concerns | No concerns | Some concerns | Moderate |
| ACUP_MB_CT:MB_CT | 0 | Some concerns | Low risk | No concerns | Major concerns | No concerns | Some concerns | Very low |
| ACUP_MB_CT:RT_CT | 0 | Some concerns | Low risk | No concerns | No concerns | No concerns | Some concerns | Low |
| ACUP_MB_CT:rTMS_ACUP_MB_CT | 0 | No concerns | Low risk | No concerns | No concerns | Major concerns | Some concerns | Low |
| ACUP_MB_CT:rTMS_CT | 0 | Some concerns | Low risk | No concerns | Major concerns | No concerns | Some concerns | Very low |
| ACUP_RT_CT:AE_CT | 0 | No concerns | Low risk | No concerns | Major concerns | No concerns | Some concerns | Low |
| ACUP_RT_CT:CFT_CT | 0 | Some concerns | Low risk | No concerns | No concerns | Major concerns | Some concerns | Very low |
| ACUP_RT_CT:EA | 0 | Some concerns | Low risk | No concerns | No concerns | No concerns | Some concerns | Low |
| ACUP_RT_CT:EA_CT | 0 | Some concerns | Low risk | No concerns | No concerns | Major concerns | Some concerns | Very low |
| ACUP_RT_CT:EMGBFB | 0 | No concerns | Low risk | No concerns | No concerns | No concerns | Some concerns | Moderate |
| ACUP_RT_CT:EMGBFB_CT | 0 | No concerns | Low risk | No concerns | Major concerns | No concerns | Some concerns | Low |
| ACUP_RT_CT:FNS_CT | 0 | No concerns | Low risk | No concerns | Major concerns | No concerns | Some concerns | Low |
| ACUP_RT_CT:HBO | 0 | Some concerns | Low risk | No concerns | No concerns | No concerns | Some concerns | Low |
| ACUP_RT_CT:HBO_CT | 0 | Some concerns | Low risk | No concerns | Major concerns | No concerns | Some concerns | Very low |
| ACUP_RT_CT:MB | 0 | No concerns | Low risk | No concerns | No concerns | Major concerns | Some concerns | Low |
| ACUP_RT_CT:MB_CT | 0 | Some concerns | Low risk | No concerns | Major concerns | No concerns | Some concerns | Very low |
| ACUP_RT_CT:rTMS_ACUP_MB_CT | 0 | No concerns | Low risk | No concerns | Major concerns | No concerns | Some concerns | Low |
| ACUP_RT_CT:rTMS_CT | 0 | Some concerns | Low risk | No concerns | Major concerns | No concerns | Some concerns | Very low |
| AE_CT:CFT_CT | 0 | No concerns | Low risk | No concerns | Major concerns | No concerns | Some concerns | Low |
| AE_CT:EA | 0 | Some concerns | Low risk | No concerns | No concerns | Major concerns | Some concerns | Very low |
| AE_CT:EA_CT | 0 | Some concerns | Low risk | No concerns | Major concerns | No concerns | Some concerns | Very low |
| AE_CT:EMGBFB | 0 | No concerns | Low risk | No concerns | No concerns | No concerns | Some concerns | Moderate |
| AE_CT:EMGBFB_CT | 0 | No concerns | Low risk | No concerns | Major concerns | No concerns | Some concerns | Low |
| AE_CT:FNS_CT | 0 | No concerns | Low risk | No concerns | Major concerns | No concerns | Some concerns | Low |
| AE_CT:HBO | 0 | Some concerns | Low risk | No concerns | No concerns | No concerns | Some concerns | Low |
| AE_CT:HBO_CT | 0 | Some concerns | Low risk | No concerns | Major concerns | No concerns | Some concerns | Very low |
| AE_CT:MB | 0 | No concerns | Low risk | No concerns | Major concerns | No concerns | Some concerns | Low |
| AE_CT:MB_CT | 0 | No concerns | Low risk | No concerns | Major concerns | No concerns | Some concerns | Low |
| AE_CT:RT_CT | 0 | No concerns | Low risk | No concerns | Major concerns | No concerns | Some concerns | Low |
| AE_CT:rTMS_ACUP_MB_CT | 0 | No concerns | Low risk | No concerns | Major concerns | No concerns | Some concerns | Low |
| AE_CT:rTMS_CT | 0 | No concerns | Low risk | No concerns | Major concerns | No concerns | Some concerns | Low |
| CFT_CT:EA | 0 | Some concerns | Low risk | No concerns | Major concerns | No concerns | Some concerns | Very low |
| CFT_CT:EA_CT | 0 | Some concerns | Low risk | No concerns | Major concerns | No concerns | Some concerns | Very low |
| CFT_CT:EMGBFB | 0 | No concerns | Low risk | No concerns | Major concerns | No concerns | Some concerns | Low |
| CFT_CT:EMGBFB_CT | 0 | No concerns | Low risk | No concerns | Major concerns | No concerns | Some concerns | Low |
| CFT_CT:FNS_CT | 0 | Some concerns | Low risk | No concerns | Major concerns | No concerns | Some concerns | Very low |
| CFT_CT:HBO | 0 | Some concerns | Low risk | No concerns | No concerns | Major concerns | Some concerns | Very low |
| CFT_CT:HBO_CT | 0 | Some concerns | Low risk | No concerns | Major concerns | No concerns | Some concerns | Very low |
| CFT_CT:MB | 0 | Some concerns | Low risk | No concerns | Major concerns | No concerns | Some concerns | Very low |
| CFT_CT:MB_CT | 0 | Some concerns | Low risk | No concerns | Major concerns | No concerns | Some concerns | Very low |
| CFT_CT:RT_CT | 0 | Some concerns | Low risk | No concerns | Major concerns | No concerns | Some concerns | Very low |
| CFT_CT:rTMS_ACUP_MB_CT | 0 | No concerns | Low risk | No concerns | Major concerns | No concerns | Some concerns | Low |
| CFT_CT:rTMS_CT | 0 | Some concerns | Low risk | No concerns | No concerns | Major concerns | Some concerns | Very low |
| CT:EMGBFB | 0 | No concerns | Low risk | No concerns | Major concerns | No concerns | Some concerns | Low |
| EA:EMGBFB | 0 | No concerns | Low risk | No concerns | Major concerns | No concerns | Some concerns | Low |
| EA:EMGBFB_CT | 0 | Some concerns | Low risk | No concerns | Major concerns | No concerns | Some concerns | Very low |
| EA:FNS_CT | 0 | Some concerns | Low risk | No concerns | No concerns | No concerns | Some concerns | Low |
| EA:HBO | 0 | Some concerns | Low risk | No concerns | Major concerns | No concerns | Some concerns | Very low |
| EA:HBO_CT | 0 | Some concerns | Low risk | No concerns | No concerns | Major concerns | Some concerns | Very low |
| EA:MB | 0 | Some concerns | Low risk | No concerns | Major concerns | No concerns | Some concerns | Very low |
| EA:MB_CT | 0 | Some concerns | Low risk | No concerns | No concerns | Major concerns | Some concerns | Very low |
| EA:RT_CT | 0 | Some concerns | Low risk | No concerns | Major concerns | No concerns | Some concerns | Very low |
| EA:rTMS_ACUP_MB_CT | 0 | Some concerns | Low risk | No concerns | Major concerns | No concerns | Some concerns | Very low |
| EA:rTMS_CT | 0 | Some concerns | Low risk | No concerns | No concerns | No concerns | Some concerns | Low |
| EA_CT:EMGBFB | 0 | No concerns | Low risk | No concerns | Major concerns | No concerns | Some concerns | Low |
| EA_CT:EMGBFB_CT | 0 | Some concerns | Low risk | No concerns | Major concerns | No concerns | Some concerns | Very low |
| EA_CT:FNS_CT | 0 | Some concerns | Low risk | No concerns | No concerns | Major concerns | Some concerns | Very low |
| EA_CT:HBO | 0 | Some concerns | Low risk | No concerns | No concerns | Major concerns | Some concerns | Very low |
| EA_CT:HBO_CT | 0 | Some concerns | Low risk | No concerns | No concerns | Major concerns | Some concerns | Very low |
| EA_CT:MB | 0 | Some concerns | Low risk | No concerns | Major concerns | No concerns | Some concerns | Very low |
| EA_CT:MB_CT | 0 | Some concerns | Low risk | No concerns | Major concerns | No concerns | Some concerns | Very low |
| EA_CT:RT_CT | 0 | Some concerns | Low risk | No concerns | Major concerns | No concerns | Some concerns | Very low |
| EA_CT:rTMS_ACUP_MB_CT | 0 | Some concerns | Low risk | No concerns | Major concerns | No concerns | Some concerns | Very low |
| EMGBFB:FNS_CT | 0 | No concerns | Low risk | No concerns | No concerns | No concerns | Some concerns | Moderate |
| EMGBFB:HBO | 0 | No concerns | Low risk | No concerns | Major concerns | No concerns | Some concerns | Low |
| EMGBFB:HBO_CT | 0 | No concerns | Low risk | No concerns | No concerns | No concerns | Some concerns | Moderate |
| EMGBFB:MB | 0 | No concerns | Low risk | No concerns | Major concerns | No concerns | Some concerns | Low |
| EMGBFB:MB_CT | 0 | No concerns | Low risk | No concerns | No concerns | Major concerns | Some concerns | Low |
| EMGBFB:RT_CT | 0 | No concerns | Low risk | No concerns | Major concerns | No concerns | Some concerns | Low |
| EMGBFB:rTMS_ACUP_MB_CT | 0 | No concerns | Low risk | No concerns | No concerns | Major concerns | Some concerns | Low |
| EMGBFB:rTMS_CT | 0 | No concerns | Low risk | No concerns | No concerns | No concerns | Some concerns | High |
| EMGBFB_CT:FNS_CT | 0 | No concerns | Low risk | No concerns | Major concerns | No concerns | Some concerns | Low |
| EMGBFB_CT:HBO | 0 | Some concerns | Low risk | No concerns | No concerns | No concerns | Some concerns | Low |
| EMGBFB_CT:HBO_CT | 0 | Some concerns | Low risk | No concerns | Major concerns | No concerns | Some concerns | Very low |
| EMGBFB_CT:MB | 0 | No concerns | Low risk | No concerns | Major concerns | No concerns | Some concerns | Low |
| EMGBFB_CT:MB_CT | 0 | No concerns | Low risk | No concerns | Major concerns | No concerns | Some concerns | Low |
| EMGBFB_CT:RT_CT | 0 | No concerns | Low risk | No concerns | Major concerns | No concerns | Some concerns | Low |
| EMGBFB_CT:rTMS_ACUP_MB_CT | 0 | No concerns | Low risk | No concerns | Major concerns | No concerns | Some concerns | Low |
| EMGBFB_CT:rTMS_CT | 0 | No concerns | Low risk | No concerns | Major concerns | No concerns | Some concerns | Low |
| FNS_CT:HBO | 0 | Some concerns | Low risk | No concerns | No concerns | No concerns | Some concerns | Low |
| FNS_CT:HBO_CT | 0 | Some concerns | Low risk | No concerns | Major concerns | No concerns | Some concerns | Very low |
| FNS_CT:MB | 0 | No concerns | Low risk | No concerns | No concerns | Major concerns | Some concerns | Low |
| FNS_CT:MB_CT | 0 | Some concerns | Low risk | No concerns | Major concerns | No concerns | Some concerns | Very low |
| FNS_CT:RT_CT | 0 | Some concerns | Low risk | No concerns | No concerns | Major concerns | Some concerns | Very low |
| FNS_CT:rTMS_ACUP_MB_CT | 0 | No concerns | Low risk | No concerns | Major concerns | No concerns | Some concerns | Low |
| FNS_CT:rTMS_CT | 0 | Some concerns | Low risk | No concerns | Major concerns | No concerns | Some concerns | Very low |
| HBO:MB | 0 | Some concerns | Low risk | No concerns | Major concerns | No concerns | Some concerns | Very low |
| HBO:MB_CT | 0 | Some concerns | Low risk | No concerns | No concerns | No concerns | Some concerns | Low |
| HBO:RT_CT | 0 | Some concerns | Low risk | No concerns | No concerns | Major concerns | Some concerns | Very low |
| HBO:rTMS_ACUP_MB_CT | 0 | Some concerns | Low risk | No concerns | No concerns | No concerns | Some concerns | Low |
| HBO:rTMS_CT | 0 | Some concerns | Low risk | No concerns | No concerns | No concerns | Some concerns | Low |
| HBO_CT:MB | 0 | Some concerns | Low risk | No concerns | Major concerns | No concerns | Some concerns | Very low |
| HBO_CT:MB_CT | 0 | Some concerns | Low risk | No concerns | Major concerns | No concerns | Some concerns | Very low |
| HBO_CT:RT_CT | 0 | Some concerns | Low risk | No concerns | No concerns | Major concerns | Some concerns | Very low |
| HBO_CT:rTMS_ACUP_MB_CT | 0 | Some concerns | Low risk | No concerns | Major concerns | No concerns | Some concerns | Very low |
| HBO_CT:rTMS_CT | 0 | Some concerns | Low risk | No concerns | Major concerns | No concerns | Some concerns | Very low |
| MB:MB_CT | 0 | No concerns | Low risk | No concerns | Major concerns | No concerns | Some concerns | Low |
| MB:RT_CT | 0 | No concerns | Low risk | No concerns | Major concerns | No concerns | Some concerns | Low |
| MB:rTMS_ACUP_MB_CT | 0 | No concerns | Low risk | No concerns | Major concerns | No concerns | Some concerns | Low |
| MB:rTMS_CT | 0 | No concerns | Low risk | No concerns | No concerns | Major concerns | Some concerns | Low |
| MB_CT:RT_CT | 0 | Some concerns | Low risk | No concerns | Major concerns | No concerns | Some concerns | Very low |
| MB_CT:rTMS_ACUP_MB_CT | 0 | No concerns | Low risk | No concerns | Major concerns | No concerns | Some concerns | Low |
| MB_CT:rTMS_CT | 0 | Some concerns | Low risk | No concerns | Major concerns | No concerns | Some concerns | Very low |
| RT_CT:rTMS_ACUP_MB_CT | 0 | No concerns | Low risk | No concerns | Major concerns | No concerns | Some concerns | Low |
| RT_CT:rTMS_CT | 0 | Some concerns | Low risk | No concerns | No concerns | Major concerns | Some concerns | Very low |
| rTMS_ACUP_MB_CT:rTMS_CT | 0 | No concerns | Low risk | No concerns | Major concerns | No concerns | Some concerns | Low |

## 10.2 ADL

10.2.1 Summary of study limitations of the included studies

The figure below showed that rTMS_CT, EA_CT, EA, HBO_CT and EMGBFB_CT had high risk of bias (RoB) studies.


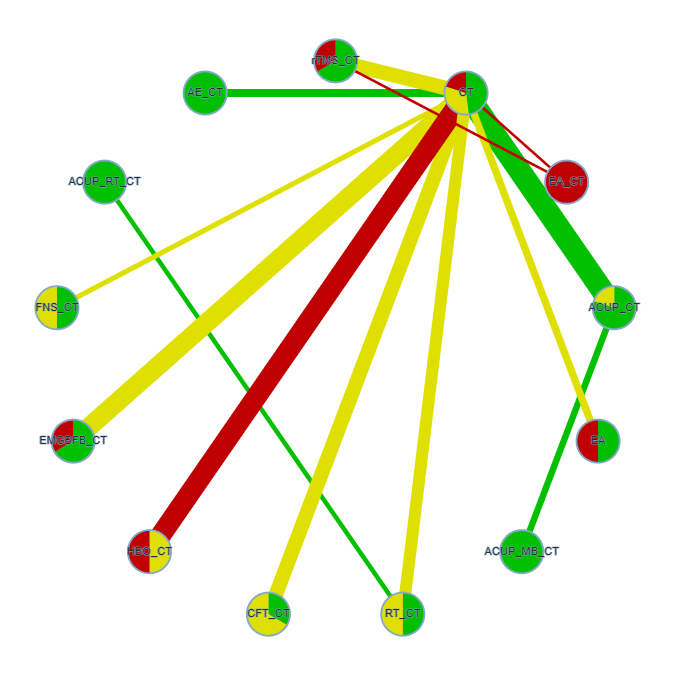


Figure 10.2.1 Network plot of study limitations of the included studies (ADL). Node size by equal size, node color by RoB. The colors in the circles indicate the percentage of low RoB studies (green), moderate RoB studies (yellow), high RoB studies (red) about each physical activity type. Edge width by sample size. Edge color by average RoB. The colors of the lines indicate the summative RoB assessment of each comparison. Low RoB is green, moderate RoB is yellow, high RoB is red.

10.2.2 Contribution percentage of low, moderate, and high RoB comparisons to each network estimate

Low RoB is green, moderate RoB is yellow, high RoB is red.


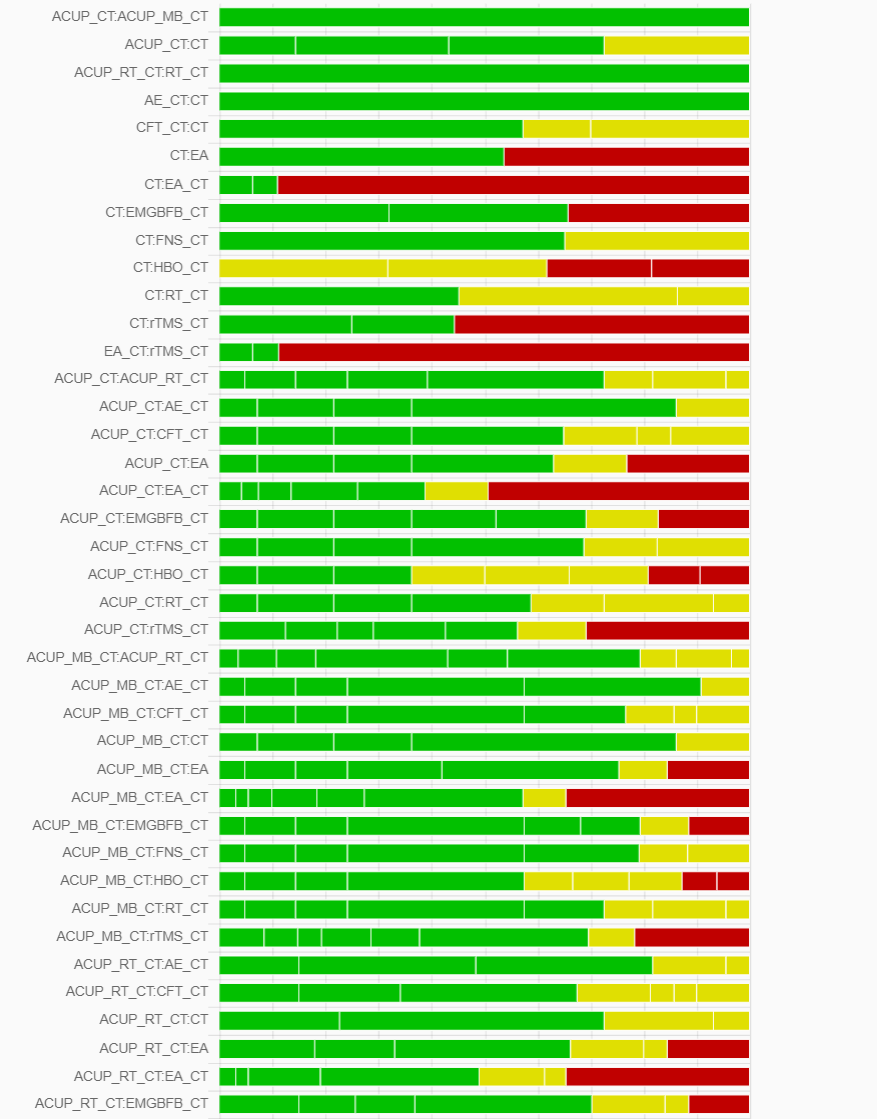


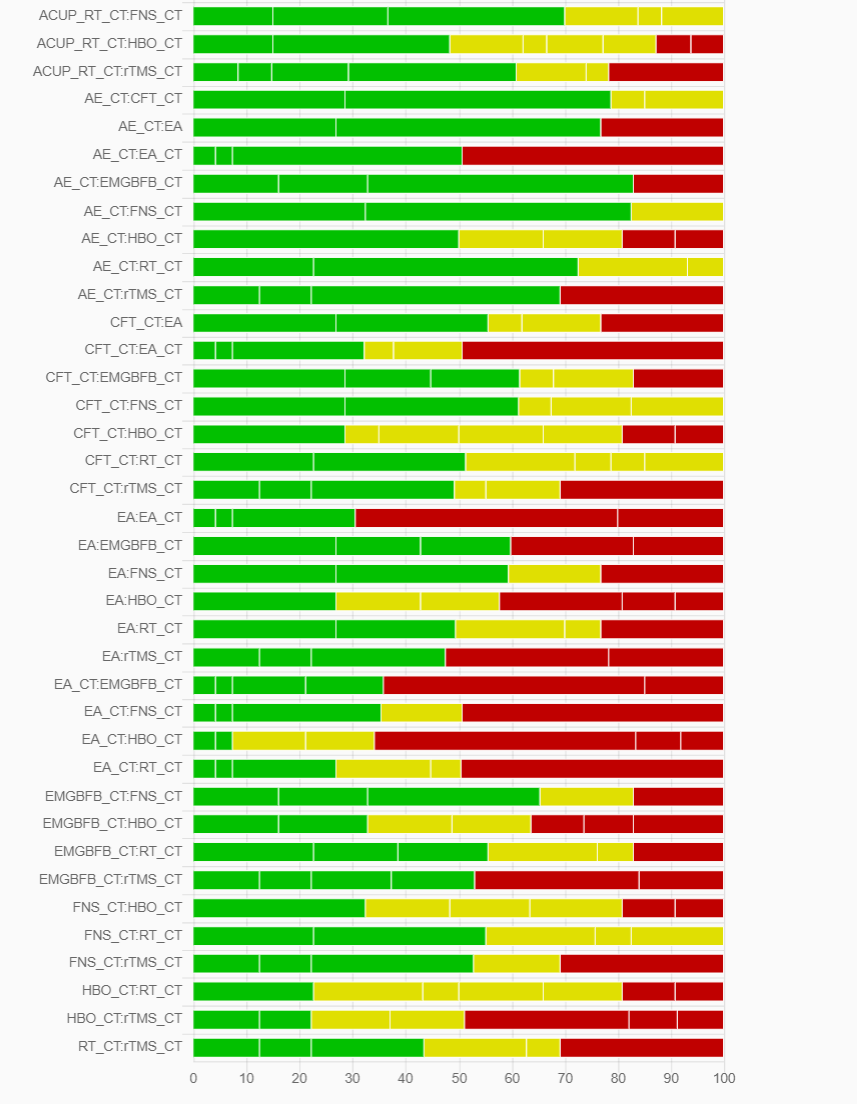


10.2.3 CINeMA for the primary outcome “ADL”

| Comparison | Number of studies | Within-study bias | Reporting bias | Indirectness | Imprecision | Heterogeneity | Incoherence | Confidence rating |
| --- | --- | --- | --- | --- | --- | --- | --- | --- |
| ACUP_CT:ACUP_MB_CT | 1 | No concerns | Low risk | No concerns | No concerns | No concerns | No concerns | High |
| ACUP_CT:CT | 4 | No concerns | Low risk | No concerns | No concerns | No concerns | No concerns | High |
| ACUP_RT_CT:RT_CT | 1 | No concerns | Low risk | No concerns | No concerns | No concerns | No concerns | High |
| AE_CT:CT | 1 | No concerns | Low risk | No concerns | No concerns | No concerns | No concerns | High |
| CFT_CT:CT | 3 | No concerns | Low risk | No concerns | No concerns | Major concerns | No concerns | Low |
| CT:EA | 2 | Some concerns | Low risk | No concerns | No concerns | Major concerns | No concerns | Very low |
| CT:EA_CT | 1 | Major concerns | Low risk | No concerns | No concerns | No concerns | No concerns | Low |
| CT:EMGBFB_CT | 3 | Some concerns | Low risk | No concerns | No concerns | No concerns | No concerns | Moderate |
| CT:FNS_CT | 2 | No concerns | Low risk | No concerns | No concerns | No concerns | No concerns | High |
| CT:HBO_CT | 4 | Some concerns | Low risk | No concerns | No concerns | No concerns | No concerns | Moderate |
| CT:RT_CT | 3 | Some concerns | Low risk | No concerns | No concerns | No concerns | No concerns | Moderate |
| CT:rTMS_CT | 3 | Some concerns | Low risk | No concerns | No concerns | No concerns | No concerns | Moderate |
| EA_CT:rTMS_CT | 1 | Major concerns | Low risk | No concerns | No concerns | No concerns | No concerns | Low |
| ACUP_CT:ACUP_RT_CT | 0 | No concerns | Low risk | No concerns | Major concerns | No concerns | No concerns | Low |
| ACUP_CT:AE_CT | 0 | No concerns | Low risk | No concerns | Major concerns | No concerns | No concerns | Low |
| ACUP_CT:CFT_CT | 0 | No concerns | Low risk | No concerns | No concerns | No concerns | No concerns | High |
| ACUP_CT:EA | 0 | Some concerns | Low risk | No concerns | No concerns | No concerns | No concerns | Moderate |
| ACUP_CT:EA_CT | 0 | Some concerns | Low risk | No concerns | No concerns | No concerns | No concerns | Moderate |
| ACUP_CT:EMGBFB_CT | 0 | No concerns | Low risk | No concerns | Major concerns | No concerns | No concerns | Low |
| ACUP_CT:FNS_CT | 0 | No concerns | Low risk | No concerns | Major concerns | No concerns | No concerns | Low |
| ACUP_CT:HBO_CT | 0 | Some concerns | Low risk | No concerns | Major concerns | No concerns | No concerns | Very low |
| ACUP_CT:RT_CT | 0 | No concerns | Low risk | No concerns | No concerns | Major concerns | No concerns | Low |
| ACUP_CT:rTMS_CT | 0 | Some concerns | Low risk | No concerns | Major concerns | No concerns | No concerns | Very low |
| ACUP_MB_CT:ACUP_RT_CT | 0 | No concerns | Low risk | No concerns | Major concerns | No concerns | No concerns | Low |
| ACUP_MB_CT:AE_CT | 0 | No concerns | Low risk | No concerns | No concerns | No concerns | No concerns | High |
| ACUP_MB_CT:CFT_CT | 0 | No concerns | Low risk | No concerns | No concerns | No concerns | No concerns | High |
| ACUP_MB_CT:CT | 0 | No concerns | Low risk | No concerns | No concerns | No concerns | No concerns | High |
| ACUP_MB_CT:EA | 0 | No concerns | Low risk | No concerns | No concerns | No concerns | No concerns | High |
| ACUP_MB_CT:EA_CT | 0 | Some concerns | Low risk | No concerns | No concerns | No concerns | No concerns | Moderate |
| ACUP_MB_CT:EMGBFB_CT | 0 | No concerns | Low risk | No concerns | No concerns | No concerns | No concerns | High |
| ACUP_MB_CT:FNS_CT | 0 | No concerns | Low risk | No concerns | No concerns | No concerns | No concerns | High |
| ACUP_MB_CT:HBO_CT | 0 | Some concerns | Low risk | No concerns | No concerns | No concerns | No concerns | Moderate |
| ACUP_MB_CT:RT_CT | 0 | No concerns | Low risk | No concerns | No concerns | No concerns | No concerns | High |
| ACUP_MB_CT:rTMS_CT | 0 | Some concerns | Low risk | No concerns | No concerns | Major concerns | No concerns | Very low |
| ACUP_RT_CT:AE_CT | 0 | No concerns | Low risk | No concerns | Major concerns | No concerns | No concerns | Low |
| ACUP_RT_CT:CFT_CT | 0 | No concerns | Low risk | No concerns | No concerns | No concerns | No concerns | High |
| ACUP_RT_CT:CT | 0 | No concerns | Low risk | No concerns | No concerns | No concerns | No concerns | High |
| ACUP_RT_CT:EA | 0 | No concerns | Low risk | No concerns | No concerns | No concerns | No concerns | High |
| ACUP_RT_CT:EA_CT | 0 | Some concerns | Low risk | No concerns | No concerns | No concerns | No concerns | Moderate |
| ACUP_RT_CT:EMGBFB_CT | 0 | No concerns | Low risk | No concerns | Major concerns | No concerns | No concerns | Low |
| ACUP_RT_CT:FNS_CT | 0 | No concerns | Low risk | No concerns | Major concerns | No concerns | No concerns | Low |
| ACUP_RT_CT:HBO_CT | 0 | Some concerns | Low risk | No concerns | Major concerns | No concerns | No concerns | Very low |
| ACUP_RT_CT:rTMS_CT | 0 | Some concerns | Low risk | No concerns | Major concerns | No concerns | No concerns | Very low |
| AE_CT:CFT_CT | 0 | No concerns | Low risk | No concerns | No concerns | No concerns | No concerns | High |
| AE_CT:EA | 0 | No concerns | Low risk | No concerns | No concerns | Major concerns | No concerns | Low |
| AE_CT:EA_CT | 0 | Some concerns | Low risk | No concerns | No concerns | No concerns | No concerns | Moderate |
| AE_CT:EMGBFB_CT | 0 | No concerns | Low risk | No concerns | Major concerns | No concerns | No concerns | Low |
| AE_CT:FNS_CT | 0 | No concerns | Low risk | No concerns | Major concerns | No concerns | No concerns | Low |
| AE_CT:HBO_CT | 0 | Some concerns | Low risk | No concerns | Major concerns | No concerns | No concerns | Very low |
| AE_CT:RT_CT | 0 | No concerns | Low risk | No concerns | Major concerns | No concerns | No concerns | Low |
| AE_CT:rTMS_CT | 0 | Some concerns | Low risk | No concerns | Major concerns | No concerns | No concerns | Very low |
| CFT_CT:EA | 0 | Some concerns | Low risk | No concerns | Major concerns | No concerns | No concerns | Very low |
| CFT_CT:EA_CT | 0 | Some concerns | Low risk | No concerns | Major concerns | No concerns | No concerns | Very low |
| CFT_CT:EMGBFB_CT | 0 | Some concerns | Low risk | No concerns | No concerns | No concerns | No concerns | Moderate |
| CFT_CT:FNS_CT | 0 | No concerns | Low risk | No concerns | Major concerns | No concerns | No concerns | Low |
| CFT_CT:HBO_CT | 0 | Some concerns | Low risk | No concerns | No concerns | No concerns | No concerns | Moderate |
| CFT_CT:RT_CT | 0 | No concerns | Low risk | No concerns | Major concerns | No concerns | No concerns | Low |
| CFT_CT:rTMS_CT | 0 | Some concerns | Low risk | No concerns | No concerns | No concerns | No concerns | Moderate |
| EA:EA_CT | 0 | Some concerns | Low risk | No concerns | Major concerns | No concerns | No concerns | Very low |
| EA:EMGBFB_CT | 0 | Some concerns | Low risk | No concerns | No concerns | No concerns | No concerns | Moderate |
| EA:FNS_CT | 0 | Some concerns | Low risk | No concerns | Major concerns | No concerns | No concerns | Very low |
| EA:HBO_CT | 0 | Some concerns | Low risk | No concerns | No concerns | Major concerns | No concerns | Very low |
| EA:RT_CT | 0 | Some concerns | Low risk | No concerns | Major concerns | No concerns | No concerns | Very low |
| EA:rTMS_CT | 0 | Some concerns | Low risk | No concerns | No concerns | No concerns | No concerns | Moderate |
| EA_CT:EMGBFB_CT | 0 | Some concerns | Low risk | No concerns | No concerns | No concerns | No concerns | Moderate |
| EA_CT:FNS_CT | 0 | Some concerns | Low risk | No concerns | Major concerns | No concerns | No concerns | Very low |
| EA_CT:HBO_CT | 0 | Major concerns | Low risk | No concerns | No concerns | No concerns | No concerns | Moderate |
| EA_CT:RT_CT | 0 | Some concerns | Low risk | No concerns | Major concerns | No concerns | No concerns | Very low |
| EMGBFB_CT:FNS_CT | 0 | Some concerns | Low risk | No concerns | Major concerns | No concerns | No concerns | Very low |
| EMGBFB_CT:HBO_CT | 0 | Some concerns | Low risk | No concerns | Major concerns | No concerns | No concerns | Very low |
| EMGBFB_CT:RT_CT | 0 | Some concerns | Low risk | No concerns | Major concerns | No concerns | No concerns | Very low |
| EMGBFB_CT:rTMS_CT | 0 | Some concerns | Low risk | No concerns | Major concerns | No concerns | No concerns | Very low |
| FNS_CT:HBO_CT | 0 | Some concerns | Low risk | No concerns | Major concerns | No concerns | No concerns | Very low |
| FNS_CT:RT_CT | 0 | No concerns | Low risk | No concerns | Major concerns | No concerns | No concerns | Low |
| FNS_CT:rTMS_CT | 0 | Some concerns | Low risk | No concerns | Major concerns | No concerns | No concerns | Very low |
| HBO_CT:RT_CT | 0 | Some concerns | Low risk | No concerns | Major concerns | No concerns | No concerns | Very low |
| HBO_CT:rTMS_CT | 0 | Some concerns | Low risk | No concerns | No concerns | Major concerns | No concerns | Very low |
| RT_CT:rTMS_CT | 0 | Some concerns | Low risk | No concerns | No concerns | Major concerns | No concerns | Very low |

# Appendix 11: PRISMA guideline

| **Section and Topic** | **Item #** | **Checklist item** | **Location where item is reported** |
| --- | --- | --- | --- |
| **TITLE** | | |  |
| Title | 1 | Identify the report as a systematic review. | No.1 |
| **ABSTRACT** | | |  |
| Abstract | 2 | See the PRISMA 2020 for Abstracts checklist. | No.1-2 |
| **INTRODUCTION** | | |  |
| Rationale | 3 | Describe the rationale for the review in the context of existing knowledge. | No.2-3 (1) |
| Objectives | 4 | Provide an explicit statement of the objective(s) or question(s) the review addresses. | No.2-3 (1) |
| **METHODS** | | |  |
| Eligibility criteria | 5 | Specify the inclusion and exclusion criteria for the review and how studies were grouped for the syntheses. | No.3 (2.2) |
| Information sources | 6 | Specify all databases, registers, websites, organisations, reference lists and other sources searched or consulted to identify studies. Specify the date when each source was last searched or consulted. | No.3 (2.1) |
| Search strategy | 7 | Present the full search strategies for all databases, registers and websites, including any filters and limits used. | No.3 (2.1) |
| Selection process | 8 | Specify the methods used to decide whether a study met the inclusion criteria of the review, including how many reviewers screened each record and each report retrieved, whether they worked independently, and if applicable, details of automation tools used in the process. | No.4 (2.4) |
| Data collection process | 9 | Specify the methods used to collect data from reports, including how many reviewers collected data from each report, whether they worked independently, any processes for obtaining or confirming data from study investigators, and if applicable, details of automation tools used in the process. | No.4 (2.4) |
| Data items | 10a | List and define all outcomes for which data were sought. Specify whether all results that were compatible with each outcome domain in each study were sought (e.g. for all measures, time points, analyses), and if not, the methods used to decide which results to collect. | No.3-4 (2.3) |
|  | 10b | List and define all other variables for which data were sought (e.g. participant and intervention characteristics, funding sources). Describe any assumptions made about any missing or unclear information. | No.4 (2.4) |
| Study risk of bias assessment | 11 | Specify the methods used to assess risk of bias in the included studies, including details of the tool(s) used, how many reviewers assessed each study and whether they worked independently, and if applicable, details of automation tools used in the process. | No.4 (2.5) |
| Effect measures | 12 | Specify for each outcome the effect measure(s) (e.g. risk ratio, mean difference) used in the synthesis or presentation of results. | No.5 (2.6) |
| Synthesis methods | 13a | Describe the processes used to decide which studies were eligible for each synthesis (e.g. tabulating the study intervention characteristics and comparing against the planned groups for each synthesis (item #5)). | No.5 (3.1) |
|  | 13b | Describe any methods required to prepare the data for presentation or synthesis, such as handling of missing summary statistics, or data conversions. | No.4-5 (2.6) |
|  | 13c | Describe any methods used to tabulate or visually display results of individual studies and syntheses. | No.4-5 (2.6) |
|  | 13d | Describe any methods used to synthesize results and provide a rationale for the choice(s). If meta-analysis was performed, describe the model(s), method(s) to identify the presence and extent of statistical heterogeneity, and software package(s) used. | No.4-5 (2.6) |
|  | 13e | Describe any methods used to explore possible causes of heterogeneity among study results (e.g. subgroup analysis, meta-regression). | No.4-5 (2.6) |
|  | 13f | Describe any sensitivity analyses conducted to assess robustness of the synthesized results. | No.4-5 (2.6) |
| Reporting bias assessment | 14 | Describe any methods used to assess risk of bias due to missing results in a synthesis (arising from reporting biases). | No.4 (2.5) |
| Certainty assessment | 15 | Describe any methods used to assess certainty (or confidence) in the body of evidence for an outcome. | No.4 (2.5) |
| **RESULTS** | | |  |
| Study selection | 16a | Describe the results of the search and selection process, from the number of records identified in the search to the number of studies included in the review, ideally using a flow diagram. | No.5 (3.1) |
|  | 16b | Cite studies that might appear to meet the inclusion criteria, but which were excluded, and explain why they were excluded. | No.4 (3.1)  Figure 1 |
| Study characteristics | 17 | Cite each included study and present its characteristics. | Table 1  Supplementary file 1: Appendix 4 |
| Risk of bias in studies | 18 | Present assessments of risk of bias for each included study. | No.4 (2.5)  Figure 2  Supplementary File 1: Appendix 5 |
| Results of individual studies | 19 | For all outcomes, present, for each study: (a) summary statistics for each group (where appropriate) and (b) an effect estimate and its precision (e.g. confidence/credible interval), ideally using structured tables or plots. | Table 2-3 |
| Results of syntheses | 20a | For each synthesis, briefly summarise the characteristics and risk of bias among contributing studies. | No.5-6 (3.2)  Supplementary File 1: Appendix 5 |
|  | 20b | Present results of all statistical syntheses conducted. If meta-analysis was done, present for each the summary estimate and its precision (e.g. confidence/credible interval) and measures of statistical heterogeneity. If comparing groups, describe the direction of the effect. | Table 2-4 |
|  | 20c | Present results of all investigations of possible causes of heterogeneity among study results. | No.7(3.8)  Table 5  Supplementary File 1: Appendix 8 |
|  | 20d | Present results of all sensitivity analyses conducted to assess the robustness of the synthesized results. | No.7(3.8)  Supplementary File 1: Appendix 8-9 |
| Reporting biases | 21 | Present assessments of risk of bias due to missing results (arising from reporting biases) for each synthesis assessed. | NO.5 (3.2)  Figure.2 |
| Certainty of evidence | 22 | Present assessments of certainty (or confidence) in the body of evidence for each outcome assessed. | NO.7 (3.7)  Supplementary File 1: Appendix 10 |
| **DISCUSSION** | | |  |
| Discussion | 23a | Provide a general interpretation of the results in the context of other evidence. | N0.7-9 (4) |
|  | 23b | Discuss any limitations of the evidence included in the review. | N0.7-9 (4) |
|  | 23c | Discuss any limitations of the review processes used. | N0.7-9 (4) |
|  | 23d | Discuss implications of the results for practice, policy, and future research. | N0.7-9 (4) |
| **OTHER INFORMATION** | | |  |
| Registration and protocol | 24a | Provide registration information for the review, including register name and registration number, or state that the review was not registered. | No.2 (2) |
|  | 24b | Indicate where the review protocol can be accessed, or state that a protocol was not prepared. | No.2(2) |
|  | 24c | Describe and explain any amendments to information provided at registration or in the protocol. | NA |
| Support | 25 | Describe sources of financial or non-financial support for the review, and the role of the funders or sponsors in the review. | No.10 |
| Competing interests | 26 | Declare any competing interests of review authors. | No.10 |
| Availability of data, code and other materials | 27 | Report which of the following are publicly available and where they can be found: template data collection forms; data extracted from included studies; data used for all analyses; analytic code; any other materials used in the review. | No.10 |
